# Constructing high-density active sites on hollow covalent organic polymers for efficient oxygen electrocatalysis

**DOI:** 10.1038/s41467-026-73508-z

**Published:** 2026-06-09

**Authors:** Shiyuan Fei, Shuai Yang, Zejin He, Ji Li, Bingbao Mei, Qing Xu, Xiaosong Liu, Zheng Jiang

**Affiliations:** 1 National Synchrotron Radiation Laboratory, University of Science and Technology of China, Hefei, China; 2 Tianmu Lake Institute of Advanced Energy Storage Technologies, Liyang, China; 3 School of Physical Science and Technology, ShanghaiTech University, Shanghai, China; 4 Shanghai Synchrotron Radiation Facility, Shanghai Advanced Research Institute, Chinese Academy of Sciences, Shanghai, China; 5 Advanced Separation & Conversion on Engineered Nanopore Dynamics Laboratory, Shanghai Advanced Research Institute, Chinese Academy of Sciences, Shanghai, China

**Keywords:** Conjugated polymers, Catalytic mechanisms

## Abstract

Creating efficient catalytic sites on covalent organic polymers (COPs) is a promising approach for green energy conversion and industrial catalysis. Although the post-synthetic modification of COPs has made progress in constructing single-atom sites and metal nanoparticles, this method remains limited in terms of the types, quantities, and overall performance of the constructing sites. To address these limitations, we develop a template-source construction strategy for catalytic site establishment. This strategy successfully yields hollow COPs with a high content of Co-O active species (H-COP-Co), demonstrating high activity as oxygen electrocatalysts. Comparing to traditional post-synthetically modified COPs with single Co sites (S-COP-Co), H-COP-Co demonstrates an increase in active metal content, from 2.96% to 56.86%, with enhanced catalytic activity for oxygen evolution reaction (OER). Unlike single Co sites that operate via the adsorbate evolution mechanism (AEM), comprehensive spectroscopic characterization and theoretical calculations reveal that the initial and reconstructed Co oxide nanoparticles in COPs operate via the oxide path mechanism, serving as the origin of the superior OER performance. These findings provide valuable insights into the design of multifunctional composite COP materials, underscoring the importance of intrinsically structural designing and modulating reaction mechanisms to enhance energy conversion efficiency.

## Abstract (web summary)

The authors employed a template-source construction strategy for a covalent organic polymer with high-density active sites for oxygen evolution reaction.

## Introduction

Covalent organic polymers (COPs) are an emerging class of polymers created by assembling molecular organic building blocks via dynamic covalent bonds. By leveraging designable monomers, these polymers demonstrate porous structures, customizable structural configurations, thermal and chemical stability, and high surface areas^1–5^. These characteristics make COPs viable for several frontier applications, such as gas, liquid, and ion adsorption and separation, energy storage and conversion, life sciences, photocatalysis, and electrocatalysis^6–11^. Building upon these applications, composite COPs have gradually emerged as a new generation of polymers, offering significant advantages of extraordinary performance over single-component COPs^12–16^. Despite these advancements, the composited COPs were usually introduced with active sites by post-modification with limited types and amounts of active sites^17,18^. Consequently, developing strategies to construct novel active units with high density remains a critical challenge and an area of particular interest.

The oxygen evolution reaction (OER) is a key process in water splitting and rechargeable metal-ion batteries, involving the coupling of multiple protons, electrons, and oxygen-containing intermediates^19–25^. Among the catalytic pathways for the OER, the adsorbate evolution mechanism (AEM) is widely acknowledged as an efficient route that proceeds through the adsorption of intermediates, including *OH, *O, and *OOH^26–29^. This pathway is commonly facilitated by single-metal sites, which induce the monodentate adsorption of intermediates and thereby favor the AEM^30–33^. However, theoretical predictions indicate that the AEM is hindered by a high overpotential of ~370 ± 100 mV^34,35^. In contrast, the oxide path mechanism (OPM) represents an ideal catalytic pathway for the OER, as it prevents the generation of lattice defects and additional *OOH intermediates, resulting in a lower overpotential^36,37^ (Supplementary Fig. 1). However, COPs, known for their modifiable groups and ability to capture metal ions, are often employed to construct single-atom catalysts favoring single-site adsorption through the AEM pathway^38–41^. Establishing the COPs with high-density sites for a suitable adsorption structure induced the OPM pathway, which was anticipated to change the status quo in catalytic OER functional COPs design.

In this study, we utilized ZIF-67, a typical metal‒organic framework (MOF), as a substrate to construct a core‒shell (COP@ZIF) structure via a mild covalent organic condensation reaction of monomers. Subsequent warm hydrolysis of the ZIF-67 core while maintaining the COP shell intact led to Co capture by the constituent functional groups, resulting in the formation of cobalt oxide aggregates (collectively termed as H-COP-Co). The traditional post-synthetically modified COPs with single Co sites (collectively termed as S-COP-Co) have previously demonstrated high potential in the catalytic field^17,42^. Differently, the H-COP-Co exhibited a controlled morphology and high density of CoO_x_ clusters, which served as reactive sites for the OER. Furthermore, comprehensive in situ observations revealed that H-COP-Co tends to aggregate into cobalt oxides and facilitates OER via the OPM pathway, demonstrating superior performance compared to the AEM pathway adopted by S-COP-Co.

## Results

### Design and characterization of the COPs

**Fig. 1:**
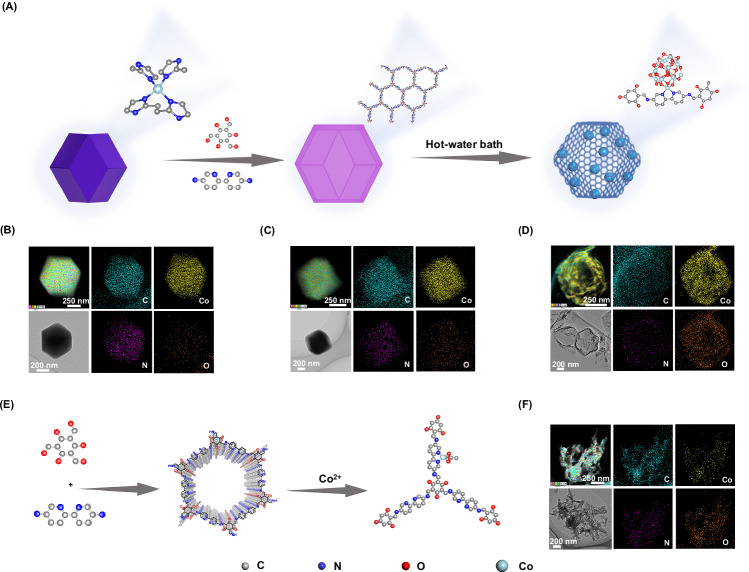
Synthesis process and morphology images of the catalysts. **A** Schematic of the synthesis process for the H-COP-Co catalyst. **B**–**D** Transmission electron microscopy (TEM) and energy-dispersive X-ray spectroscopy (EDS) mapping images of ZIF-67 (**B**), COP@ZIF (**C**), and H-COP-Co (**D**). **E** Schematic of the synthesis process for the S-COP-Co catalyst. **F** TEM and EDS mapping images of S-COP-Co.

First, ZIF-67 was synthesized via a typical solvothermal reaction using cobalt(II) nitrate hexahydrate and 2-methylimidazole. COP@ZIF was then synthesized via a mild polyreaction involving the condensation of 1,3,5-benzenetricarboxaldehyde-2,4,6-trihydroxy with [2,2’-bipyridine]−5,5’-diamine on the ZIF-67 substrate. This process resulted in a core‒shell COP@ZIF structure, with ZIF-67 acting as the core and COP layers forming the shell. To further process the material, COP@ZIF powders were mixed in water and heated for several minutes. During this step, the thermal attack of water molecules broke the metal‒organic bonds between Co ions and 2-methylimidazole, leaving the COP structure intact. The high-density hydrated Co ions subsequently aggregated into oxide clusters. The abundant dipyridyl groups in the COP facilitated the capture of both Co atoms and oxide clusters, leading to the formation of hollow COPs enriched with Co-O active species (H-COP-Co) (Fig. 1A). For comparison, we also employed the traditional method to synthesize single-atom catalysts. In this approach, COPs were synthesized first and then post-synthetically modified with Co ions to yield COPs containing single Co sites (S-COP-Co) (Fig. 1E).

**Fig. 2:**
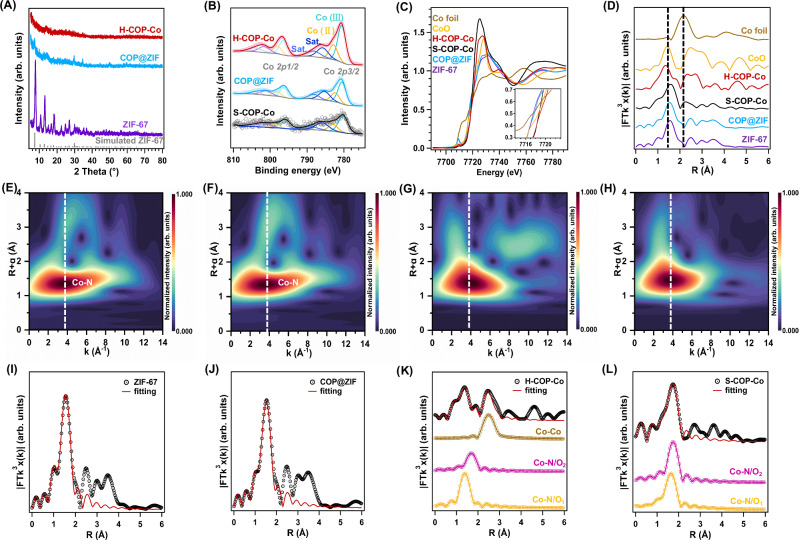
Structural characterization of the catalysts. **A** PXRD patterns of ZIF-67, COP@ZIF, and H-COP-Co. **B** Co 2*p* spectra of S-COP-Co, COP@ZIF, and H-COP-Co. **C** XANES spectra and **D** corresponding k^3^-weighted Fourier transformed (FT) spectra (brown: Co foil, purple: ZIF-67, blue: COP@ZIF, black: S-COP-Co, red: H-COP-Co, orange: CoO, *k* = 2.5‒10.5 Å^−1^). Wavelet transform contour spectra derived from the Co K-edge k^2^-weighted EXAFS data of **E** ZIF-67, **F** COP@ZIF, **G** H-COP-Co, and **H** S-COP-Co. Co K-edge k^3^-weighted FT spectra derived from the EXAFS data of **I** ZIF-67, **J** COP@ZIF, **K** H-COP-Co, and **L** S-COP-Co (black dots: experimental data; red: fitting results).

Powder X-ray diffraction (PXRD) measurements were conducted to monitor the evolution of the crystal structure during the synthesis process (Fig. 2A). The diffraction pattern of ZIF-67 agreed well with previously reported data, displaying intense peaks at 7.8°, 10.8°, 13.1°, and 18.2°, which were assigned to the (011), (002), (112), and (222) facets, respectively^43–45^. However, after coating COPs on the surfaces of ZIF crystals, the intensities of these diffraction peaks decreased owing to the low crystallinity of the COPs. Further changes were observed in the XRD pattern of H-COP-Co, which displayed the typical characteristics of amorphous polymers, with no identifiable peaks. This is likely attributed to the destruction of the core framework during the synthesis process. In contrast, S-COP-Co and the COP exhibited similar peaks from crystalline polymers, indicating the single Co sites on the S-COP-Co (Supplementary Fig. 2).

The morphological evolution of the samples was tracked using TEM and EDS analyses. The TEM images of ZIF-67 revealed typical cubic structures with sizes ranging from ~500 to ~700 nm (Fig. 1B). The corresponding EDS analysis confirmed that Co, N, and C elements were uniformly distributed within these ZIF-67 cubes. The TEM images of COP@ZIF, synthesized via the covalent organic reaction, also revealed similar cubic structures but with rough surfaces (Fig. 1C). The corresponding EDS results indicated that all elements were clustered within the core of the cubes, while C and O elements were also distributed on the shell and extended beyond it, providing evidence of the COP coating on the ZIF-67 structure. For the same reason, the Fourier transform infrared spectroscopy (FTIR) measurements also demonstrated the COP coating on the ZIF-67 (Supplementary Fig. 3). Thermogravimetric analysis (TGA) displayed the retain weight at 1000 °C result showed the value of COP@ZIF between COP and ZIF, close to that of ZIF-67 since ZIF-67 occupied the main component (Supplementary Fig. 4A). And the differential scanning calorimetry (DSC) curves also demonstrated the COP coating on the ZIF-67 in COP@ZIF (Supplementary Fig. 4B). Subsequent hydrolysis transformed COP@ZIF into H-COP-Co, which displayed several hollow cubic structures that could be identified as COP shells with 10 ~ 30 nm thickness (Fig. 1D, Supplementary Fig. 5). Aggregated metal atoms were observed on the shells. EDS mapping revealed that the C, N, O, and Co elements were primarily located on the shells, although the N content was lower compared to that in COP@ZIF (Fig. 1D). This reduction in N content was attributed to the loss of 2-methylimidazole, while the observed N species originated from the COP skeleton. The O content was high, attributed to the formation of CoO_x_ species. We also find the lattice fringe belongs to Co oxides in the high-resolution TEM images (Fig. 1D, Supplementary Fig. 5). Moreover, the scanning electron microscopy (SEM) images also displayed the H-COP-Co, also supporting the material evolution from COP@ZIF (Supplementary Fig. 6 and 7). To gain further insights, the individual species and local structures were investigated using additional characterization techniques. An analysis of the morphology of S-COP-Co revealed the localization of Co, N, and O species within the same region, indicating potential bonding interactions (Fig. 1F).

X-ray photoelectron spectroscopy (XPS) was performed to analyze the valence states and chemical composition of COP@ZIF and H-COP-Co. The C, N, O, and Co contents of COP@ZIF were estimated to be 55.1 wt.%, 13.21 wt.%, 15.1 wt.%, and 16.59 wt.%, respectively. In contrast, the C, N, O, and Co contents of H-COP-Co were 15.56 wt.%, 1.84 wt.%, 32.96 wt.%, and 49.64 wt.%, respectively (Supplementary Table 1), indicating a dramatic increase in the Co and O contents of H-COP-Co. This increase was consistent with the thermogravimetric curve of H-COP-Co, where high weight retention suggested higher Co content (Supplementary Fig. 8). Furthermore, the high-resolution Co spectra of COP@ZIF, H-COP-Co, and S-COP-Co displayed a similar outline, which could be deconvoluted into peaks corresponding to the composition of +2 and +3 oxidation states’ peaks and other satellite features^46,47^ (Fig. 2B and Supplementary Fig. 9). It is noticed that the COP@ZIF also contains a lot of Co, but displays a lower peak intensity in XPS, since the covered COP decreased the signal. To confirm these findings and quantify the Co content more accurately, inductively coupled plasma-atomic emission spectrometry (ICP-AES) measurements were performed. The Co contents determined by ICP-AES were 56.86 wt.% for H-COP-Co and 25.43 wt.% for COP@ZIF (Supplementary Table 2). The differences in Co/O contents between COP@ZIF and H-COP-Co were attributed to the disintegration of the ZIF-67 core during the synthesis process. This disintegration led to substantial C and N loss from 2-methylimidazole, while Co atoms were captured by the COPs, where they were stored and subsequently formed oxide species (Supplementary Fig. 10).

X-ray absorption spectroscopy (XAS) is a powerful tool for elucidating the coordination environments and electronic states of specific elements^48^. Accordingly, we collected the X-ray absorption near-edge structure (XANES) and extended X-ray absorption fine structure (EXAFS) spectra of H-COP-Co and S-COP-Co. The Co K-edge XANES spectra correspond to the transition of electrons from the core 1 *s* orbital to unoccupied 4*p* orbitals, providing insights into the oxidation states of Co atoms^49^. To estimate these valence states, the XANES spectra of ZIF-67, COP@ZIF, H-COP-Co (+2.23), and S-COP-Co (+2.09) were compared to those of standard samples of Co_2_O_3_ (+3) and CoO (+2) (Supplementary Fig. 11). ZIF-67 was selected as a reference sample owing to its high crystallinity and precise local structure, where Co atoms are tetrahedrally coordinated with four N atoms from 2-methylimidazole. The XANES spectrum is sensitive to the structural symmetry of the coordination ligands surrounding Co atoms^50^. A comparison between the XANES spectra of ZIF-67 and COP@ZIF revealed similar profiles, suggesting that ZIF-67 retained its coordination configuration even after coating with COPs (Fig. 2C). To investigate the structural features in more detail, Fourier-transformed (FT) k^3^-weighted EXAFS spectra of the samples were analyzed. In the corresponding spectra of both ZIF-67 and COP@ZIF, the primary peak at 1.53 Å was attributed to the Co‒N scattering path from organic ligands (Fig. 2D). Additionally, wavelet transform EXAFS analysis identified backscattering atoms from signals in both k-space and radial distance^51^. The k-coordinate positions of the centers were associated with the backscattering atom type, and for ZIF-67 and COP@ZIF, these positions were located at ~3.95 Å^−1^ (Fig. 2E, F). Notably, these positions were close to the Co-O location in CoO (Supplementary Fig. 12), further confirming Co-N coordination in ZIF-67 and COP@ZIF. In contrast, H-COP-Co displayed a significantly different XANES spectrum compared to ZIF-67, suggesting that the hydrolysis process caused substantial structural changes. The pre-edge peaks are attributed to the transition of 1 *s* to the 4*p* component in *d*-*p* hybridized orbital and depend on the coordination symmetry of the center metal^52,53^. Therefore, the pre-edge peaks were fitted to analyze the local structural differences between the samples. Compared to ZIF-67, which exhibits tetrahedral coordination with a high peak area, H-COP-Co and S-COP-Co generated the lower peak areas (Supplementary Fig. 13). The high symmetry usually indicates a low pre-edge peak area; this indicates the Co atoms in H-COP-Co and S-COP-Co have a possible higher symmetry configuration different from the tetrahedral coordination. Next, the FT k^3^-weighted EXAFS spectra of the samples were analyzed to get more accurate structural information. Both ZIF-67 and COP@ZIF showed similar results, with a single Co‒N bonding path forming the first coordination shell. The coordination number (C.N.) was determined to be 4.0, and the bond length was 2.01 Å (Fig. 2I, J). In contrast, the fitting results for H-COP-Co revealed a more complex structure, comprising two Co-N/O paths (Co-N/O_1_ and Co-N/O_2_) and a Co-Co path. Since the similar atomic distances of N and O, we have merged the Co-N and Co-O as the Co-N/O paths^54^. The C.N. values for Co-N/O_1_, Co-N/O_2_, and Co-Co were 2.8, 1.2, and 1.0, with bond lengths of 2.06 Å, 2.12 Å, and 2.85 Å, respectively (Fig. 2K and Supplementary Table 3). Combined with other characterization results, the two Co-N/O paths contain the Co-N and Co-O from the single Co sites, and most of them are contributed by the CoO_x_ clusters^55^. The low average Co-Co C.N. is attributed to the nanometer size effects, as C.N. is not as large as normal bulk Co oxides, and the single Co sites in H-COP-Co also decrease the average C.N. of Co-Co. The FT k^3^-weighted EXAFS spectra of S-COP-Co agreed with previous reports and featured patterns corresponding to Co-N coordination from dipyridyl units and Co-O coordination from acetate. Fitting results indicated that the C.N. values for Co-N/O_1_ and Co-N/O_2_ were 2.5 and 2.0, with bond lengths of 1.97 Å and 2.13 Å, respectively (Fig. 2L and Supplementary Table 3).

### Electrocatalytic performance

**Fig. 3:**
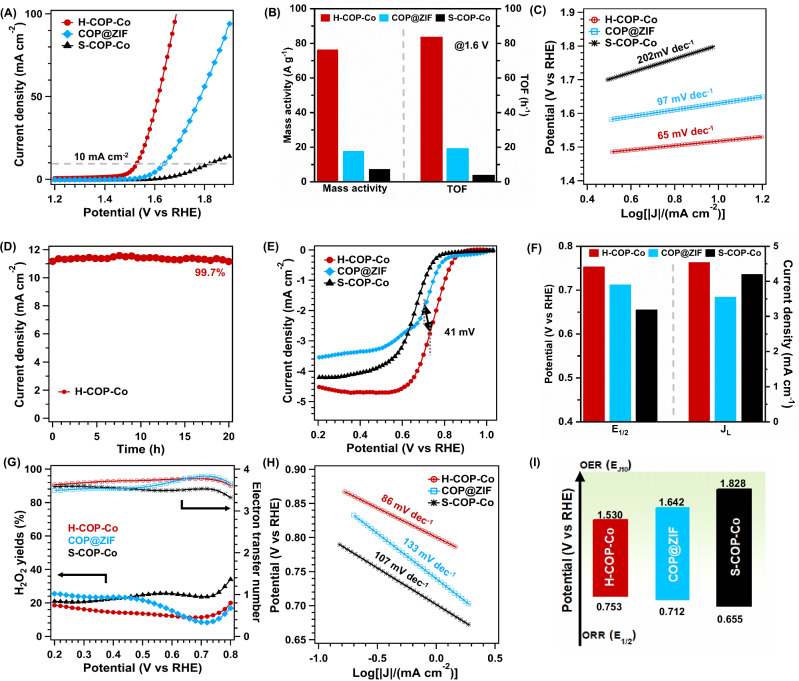
Catalytic performance of the catalysts in the OER and ORR. Catalytic performance of H-COP-Co during the OER and ORR. **A** LSV curves for H-COP-Co, COP@ZIF, and S-COP-Co during the OER in 1 M KOH without iR correction, estimated the resistance of 9.58 Ω, 10.14 Ω, and 13.47 Ω, respectively. **B** Mass activity and TOF values of the samples for the OER at 1.6 V. **C** Tafel plots derived from the iR corrected OER LSV curves. **D** Durability test of chronoamperometry curves for H-COP-Co. **E** LSV curves of H-COP-Co, COP@ZIF, and S-COP-Co during the ORR in an O_2_-saturated 0.1 M KOH solution at 1600 rpm. **F** Half-wave potential (E_1/2_) and limited current density (J_L_) of the catalyst samples. **G** H_2_O_2_ yield and electron transfer number determined from rotating ring disk electrode (RRDE) measurements for the ORR over H-COP-Co, COP@ZIF, and S-COP-Co. **H** Tafel plots derived from the iR corrected ORR LSV curves. **I** Comparison of the bifunctional catalytic performance of H-COP-Co, COP@ZIF, and S-COP-Co.

To evaluate the effectiveness of the template-source construction strategy compared to the traditional post-synthesis method in heterogeneous catalysis, the electrocatalytic OER performance of each sample was assessed in an alkaline aqueous electrolyte (1 M KOH) using a three-electrode system. To prepare the working electrodes (WEs), the catalysts were dispersed into inks, which were subsequently deposited onto glassy carbon rotating disk electrodes. Linear scan voltammetry (LSV) curves were recorded under N_2_-saturated conditions at a WE rotation speed of 1600 rpm and without iR compensation. The LSV curves in Fig. 3A reveal oxidation currents of all samples at potentials exceeding 1.23 V vs. RHE. The calculated overpotentials followed the trend H-COP-Co <COP@ZIF < S-COP-Co, with values of 300 mV, 412 mV, and 598 mV, respectively. In addition to overpotential, the mass activities of the samples were also calculated. H-COP-Co exhibited the highest mass activity of 76.30 A g^−1^, which was 10.4 times higher than that of S-COP-Co. Considering the involvement of different OER mechanisms (OPM and AEM) utilizing varying numbers of Co atoms at active sites, the turnover frequencies (TOFs) of H-COP-Co and S-COP-Co were calculated to be 83.88 h^−1^ and 4.02 h^−1^, respectively (Fig. 3B). The distinct mechanisms of these samples will be explored in subsequent sections. To assess the kinetics of the OER process, the Tafel slopes of the three samples were determined, revealing values of 65 mV dec^−1^, 97 mV dec^−1^, and 202 mV dec^−1^ for H-COP-Co, COP@ZIF, and S-COP-Co, respectively (Fig. 3C). The lower Tafel slope of H-COP-Co indicates faster reaction kinetics compared to S-COP-Co, further supporting the distinct intrinsic OER mechanisms suggested by the TOF results. As shown in electrochemical impedance spectroscopy (EIS) Nyquist plotting, the H-COP-Co displayed the smallest charge transfer resistance of these samples, which implied its fastest charge transfer (Supplementary Fig. 14). The electrochemically active surface areas of the samples were also evaluated using double-layer capacitance (C_dl_) values derived from cyclic voltammetry (CV) tests. Calculations revealed that the C_dl_ values of H-COP-Co, COP@ZIF, and S-COP-Co, obtained from half the slopes of the CV plots, were 8.6, 5.9, and 0.9 mF cm^−2^, respectively. Among the three samples, the highest C_dl_ value of H-COP-Co highlights its superior surface exposure for electrochemical reactions (Supplementary Fig. 15). Finally, the stability of H-COP-Co was tested through chronoamperometry at 1.6 V vs. RHE. H-COP-Co demonstrated nice durability, maintaining 99.7% of its initial current after 20 h. (Fig. 3D).

The electrocatalytic performance of each sample in the reverse reaction, the ORR, was evaluated in an O_2_-saturated 0.1 M KOH aqueous electrolyte using a three-electrode system. CV was first performed to verify the oxygen reduction activity of the samples by introducing different gases (N_2_ and O_2_) into the electrolyte. Closed CV curves were observed for all samples under both gas atmospheres. Notably, a single reduction peak was detected under O_2_ conditions, while no peaks were observed under N_2_ conditions, confirming that the reduction current originated from the ORR rather than other reactions (Supplementary Fig. 16). To further compare the ORR activity of the samples, LSV tests were conducted. The half-wave potential (E_1/2_) of H-COP-Co was 0.753 V, which was higher than those of COP@ZIF (0.712 V) and S-COP-Co (0.655 V) (Fig. 3E, F). The reaction selectivity was then assessed using rotating ring-disk electrode (RRDE) tests. H-COP-Co demonstrated a high proportion of the four-electron process during the ORR, with an electron transfer number of 3.60–3.77, compared to 3.32–3.66 for S-COP-Co (Fig. 3G). Additionally, the Tafel slope for the ORR was calculated to evaluate the reaction kinetics. The results revealed the following trend: H-COP-Co (86 mV dec^−1^) < S-COP-Co (107 mV dec^−1^) < COP@ZIF (133 mV dec^−1^), indicating the superior kinetic performance of H-COP-Co (Fig. 3H). To assess the kinetic current density (J_k_) of the samples, LSV curves were recorded at different rotation speeds and analyzed using the Koutecky–Levich equation. H-COP-Co exhibited the highest J_k_ value among the three samples across the Faradaic potential range. Specifically, at 0.6 V vs. RHE, the J_k_ value of H-COP-Co reached 16.9 mA cm^−2^, which was 2.4 times that of S-COP-Co (Supplementary Fig. 17). Finally, the durability of H-COP-Co was evaluated, revealing excellent stability (Supplementary Fig. 18). Overall, the electrocatalysis performance of H-COP-Co in both the ORR and OER surpasses that of S-COP-Co and COP@ZIF and is comparable to that of some reported polymer-based electrocatalysts (Fig. 3I and Supplementary Table 4).

### Mechanistic insights into the electrocatalytic activity of the samples

**Fig. 4:**
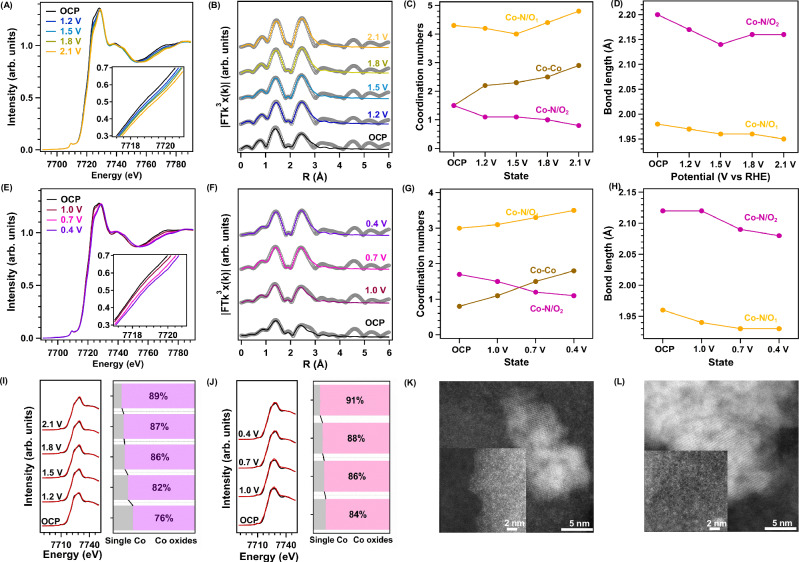
In situ XAFS measurements and scanning transmission electron microscopy images. **A** In situ Co K-edge XANES spectra of H-COP-Co during the OER. **B** Fitted Co K-edge k^3^-weighted FT EXAFS spectra of H-COP-Co at different applied voltages during the OER. **C** Statistical chart of EXAFS fitting results for the C.N. values of Co-N/O_1_ (green), Co-N/O_2_ (magenta), and Co-Co (brown) during the OER. **D** Statistical chart of EXAFS fitting results for the bond lengths of Co-N/O_1_ (green) and Co-N/O_2_ (magenta) during the OER. **E** In situ Co K-edge XANES spectra of H-COP-Co during the ORR. **F** Fitted Co K-edge k^3^-weighted FT EXAFS spectra of H-COP-Co at different applied voltages during the ORR. **G** Statistical chart of EXAFS fitting results for the C.N. values of Co-N/O_1_ (green), Co-N/O_2_ (magenta), and Co-Co (brown) during the ORR. **H** Statistical chart of EXAFS fitting results for the bond lengths of Co-N/O_1_ (green) and Co-N/O_2_ (magenta) during the ORR. **I** Left: normalized XANES spectra (black) and corresponding LCF results (red); right: species content analysis based on LCF results at different applied voltages during the OER. **J** Left: normalized XANES spectra (black) and corresponding LCF results (red); right: species content analysis based on LCF results at different applied voltages during the ORR. High-angle annular dark-field scanning transmission electron microscopy (HAADF-STEM) images of H-COP-Co **K** before and **L** after reaction (scale bar: 5 nm), showing the single atom sites (insert image, scale bar: 2 nm) and nanoparticle areas.

To examine the significant gap in the oxygen electrocatalysis performance of H-COP-Co and S-COP-Co, the underlying reaction mechanisms were explored using advanced in situ spectroscopic techniques (Supplementary Fig. 19). As mentioned previously, XAS is a powerful tool for elucidating the local structures and electronic states of metal atoms^56–59^. Accordingly, in situ XAS experiments were conducted to study the structural and electronic evolution of H-COP-Co under OER and ORR conditions. The first set of analyses focused on the in situ XANES spectra of H-COP-Co, collected at various OER states, including the open-circuit potential (OCP), 1.2 V, 1.5 V, 1.8 V, and 2.1 V (Fig. 4A). While the overall outline of the XANES spectra remained largely consistent, slight changes were observed with increasing potential. The oxidation states of Co elements at these applied potentials were evaluated based on shifts in the absorption edge positions^60^. These shifts revealed that as the potential increased from non-Faradaic to reaction conditions, the average valence states of Co atoms in the COP framework also increased, indicating gradual oxidation of the Co species at higher oxidation potentials. The structural evolution of the samples was examined using EXAFS. As depicted in Fig. 4B, the k^3^-weighted EXAFS spectra exhibited consistent profiles with two prominent peaks, among which the intensity of the peak at 2.45 Å increased with the applied oxidation potential. This peak, attributed to the Co-Co scattering path in oxides, suggests the growth of oxide particles as the reaction progressed. For a more detailed understanding of these structural changes, EXAFS fitting was performed. This analysis considered three scattering paths: two Co-N/O paths and a longer Co-Co path (Supplementary Table 5). To differentiate between the two Co-N/O paths, the shorter path was designated as Co-N/O_1_, while the longer path was defined as Co-N/O_2_. Based on previous analyses, Co-N/O_1_ was attributed to Co‒O bonds in cobalt oxides, whereas Co-N/O_2_ corresponded to Co-N coordination at the edges of cobalt oxide clusters or single Co atoms. Line charts were plotted to illustrate the evolution of parameters, such as C.N. values and bond lengths, under applied potentials within the OER range. The C.N. of Co-N/O_2_ decreased from 1.5 to 0.8, while that of Co-N/O_1_ increased from 4.3 to 4.8. Additionally, the Co-Co C.N. increased from 1.5 to 2.9 (Fig. 4C). Regarding bond lengths, Co-N/O_1_ and Co-N/O_2_ bonds contracted with increasing potential, whereas Co‒Co bond lengths remained constant (Fig. 4D). These changes in coordination suggest reconstruction of Co sites in H-COP-Co during the OER. Specifically, the increase in Co-Co C.N. indicates the growth of cobalt oxide structures, while the decrease in Co-N/O_2_ C.N. suggests a reduction in the number of single Co sites. This phenomenon implies a transformation of isolated Co atoms into cobalt oxides. To validate this observation, the linear combination fitting (LCF) method was employed to analyze component evolution using in situ XANES curves^61^. As XANES spectra are sensitive to the spatial structures and symmetry of target atoms, the S-COP-Co XANES curve was used to represent the single Co site component, while standard cobalt oxide XANES curves were used to represent the oxide component. The analysis revealed that under OCP conditions, the single Co site content was 24%, while cobalt oxides accounted for 76%. As the applied potentials increased to 1.2 V, 1.5 V, 1.8 V, and 2.1 V, the single Co site content decreased progressively to 18%, 14%, 13%, and 11%, respectively, while the cobalt oxide content increased to 82%, 86%, 87%, and 89% (Fig. 4I). These results confirm that Co sites tend to form more cobalt oxides under OER potentials. The average oxidation states of Co in H-COP-Co demonstrated an increasing trend at applied potentials close to the Faradaic range during the OER.

In situ XAFS measurements of H-COP-Co were also conducted under ORR conditions. According to the results, the structural evolution of Co atoms resembled that observed under OER conditions (Supplementary Table 6). Similar to the OER analysis, key parameters such as oxidation states, C.N. values, bond lengths, and LCF components were assessed using XANES and EXAFS fitting (Fig. 4E, F). Consistent with the trends observed under OER conditions, the C.N. of Co-N/O_2_ decreased from 1.7 to 1.1, while that of Co-N/O_1_ increased from 3.0 to 3.5, and that of Co-Co increased from 0.8 to 1.8 (Fig. 4G). In terms of bond lengths, both Co-N/O_1_ and Co-N/O_2_ bonds contracted, whereas the Co‒Co bond length remained constant (Fig. 4H). To further validate these findings, LCF analysis was performed under ORR conditions. The results revealed a similar component evolution to that observed under OER conditions, characterized by a decrease in single Co sites and an increase in cobalt oxide content (Fig. 4J). Co oxidation states were also evaluated using Co K-edge XANES analysis, which confirmed that both OER and ORR processes increased the average valence state of Co atoms in H-COP-Co (Supplementary Fig. 20). Additionally, atomic-resolution HAADF-STEM provided further evidence of structural changes. The initially highly dispersed Co atoms were observed to aggregate after the reaction, confirming the gradual formation or increased size of cobalt oxide clusters under electrocatalytic conditions (Fig. 4K, L). The content of the Co atoms in H-COP-Co is decreased (Supplementary Table 2 and 7). The PXRD and XPS of after-reacted H-COP-Co demonstrated that the phase and chemical properties of H-COP-Co are maintained, while the morphology is not kept as the initial, which may be caused by the local Co sites reconstruction and migration, inducing the evolution of bulk COP substrates (Supplementary Figs. 21–23). Thus, the structural evolution of H-COP-Co during both OER and ORR electrocatalysis followed a consistent trend toward the formation of cobalt oxides. This transformation enhances its bifunctional activity for air electrodes and ensures stability in zinc-air batteries (ZABs).

**Fig. 5:**
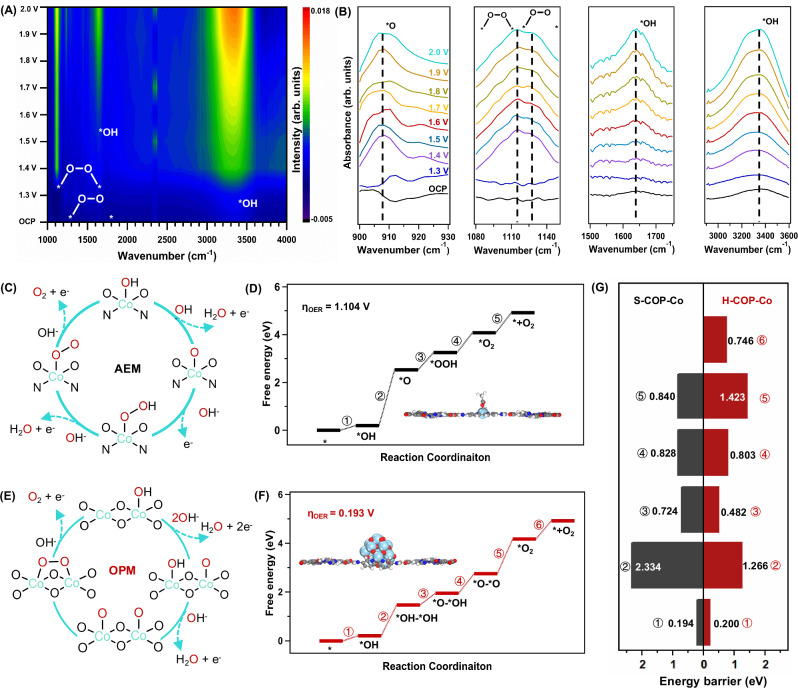
In situ attenuated total reflectance Fourier transform infrared (ATR-FTIR) spectroscopy measurements and DFT calculations. **A** In situ ATR-FTIR spectra of H-COP-Co during the electrocatalytic OER (modified the baseline by offset of 0.007 to the values of 1.4 V ~ 2.0 V for better observing the signs). **B** Differential infrared signals in the regions of 900–930 cm^−1^, 1000–1140 cm^−1^, 1500–1750 cm^−1^, and 2900–3600 cm^−1^. **C** Schematic representation of the AEM pathways on S-COP-Co. **D** Free energy diagram of the AEM pathway at 1.23 V versus RHE. **E** Schematic representation of the OPM pathways on H-COP-Co. **F** Free energy diagram of the OPM pathway at 1.23 V versus RHE. **G** Calculated free energy changes for intermediates of S-COP-Co and H-COP-Co.

The OER reaction mechanism of H-COP-Co was investigated using in situ attenuated total reflectance Fourier transform infrared (ATR-FTIR) spectroscopy (Supplementary Fig. 24). FTIR spectra were collected under OCP conditions and at applied potentials ranging from 1.3 V to 2.0 V (Fig. 5A). As depicted in Fig. 5B, distinct peaks were observed at approximately bands of ~907 cm^−1^, ~1114 cm^−1^, ~1639 cm^−1^, and ~3340 cm^−1^. These wavenumber regions correspond to oxygen intermediates involved in the OER process^62^. The increasing intensity of these peaks indicates enhanced adsorption of intermediates, which correlates with rising current during the reaction. As the oxidation potential was gradually applied, signals corresponding to adsorbed small molecules progressively intensified. A detailed analysis of the spectra revealed that the bands at ~1639 and ~3340 cm^−1^ correspond to the bending vibration and stretching vibration of *O‒H, respectively. Notably, the coupling oxygen signal at 1080‒1140 cm^−1^ exhibited overlapping peaks, which were identified as *‒O‒O‒* (~1114 cm^−1^) and *‒O‒O‒ (~1126 cm^−1^)^63^. These observations suggest that oxygen atom coupling occurs prior to the oxidation of *O to *OOH, deviating from the typical AEM pathway that progresses through *OH, *O, *OOH, and finally *O_2_^64^ (Fig. 5C). The in situ ATR-FTIR analysis indicates that H-COP-Co instead follows the OPM pathway (Fig. 5E).

Density functional theory (DFT) calculations can be regarded as a reference that was performed to investigate the catalytic activities of the AEM and OPM pathways for the OER on single Co sites and Co oxide components in COPs^65^. Two COP models were constructed, one containing single Co sites and the other featuring Co oxide clusters attached to dipyridyl groups (Supplementary Data 1). The Bader charge and charge difference density result showed that the CoO_x_ model comprises many accessible Co atoms for the OER (Supplementary Figs. 25–27). The projected density of states (PDOS) displayed the different d band of the single Co site model and the CoO_x_ model. The higher d band center for single Co sites indicated the stronger adsorption to intermediates, which may indicate the high energy barrier for intermediates oxidation and desorption (Supplementary Fig. 28). The single Co sites in the COP were studied from the perspective of the AEM pathway. The same pathway was used to evaluate the free energy profile of the four-electron transfer process. The rate-determining step (RDS) was the transition from *OH to *O, characterized by a large energy gap of 2.334 eV. This resulted in a theoretical overpotential of 1.104 V (Supplementary Figs. 29–34). In contrast, the Co oxide cluster model exhibited lower free energy changes throughout the OER process, following the OPM pathway (Supplementary Figs. 35–40). For this model, the RDS corresponded to the transition from*O‒*O to *O_2_, with a free energy change of 1.423 eV (Fig. 5D). This lower energy barrier translated to a reduced theoretical overpotential of 0.193 V (Fig. 5F). These calculations inferred that the Co oxide clusters in COPs, operating through the OPM pathway, enhance the OER process compared to single Co sites, operating through the AEM pathway (Fig. 5G).

### ZAB performance

**Fig. 6:**
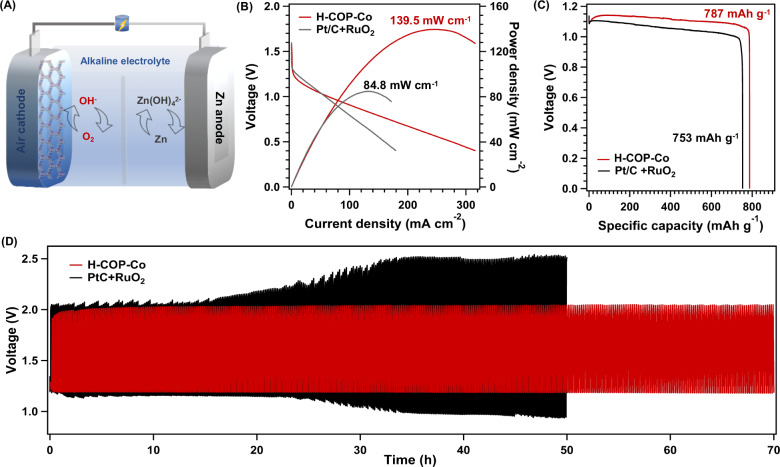
Performance comparison of ZABs based on H-COP-Co and Pt/C + RuO_2_. **A** Schematic configuration of the custom-built ZABs. **B** Discharge polarization and corresponding power density curves of ZABs with H-COP-Co and Pt/C + RuO_2_ cathodes. **C** Zn-mass-normalized specific capacities of the ZABs at a constant current density of 10 mA cm^−2^. **D** Results of galvanostatic charge/discharge cycling tests for the ZABs at a current density of 5 mA cm^−2^.

Building on the promising electrocatalytic performance and stability of H-COP-Co, we assembled a ZAB to evaluate its performance as a cathode material for OER/ORR during charge‒discharge cycles (Fig. 6A). Compared to a ZAB with a commercial Pt/C + RuO_2_ electrode, the ZAB with the H-COP-Co air cathode demonstrated a comparable open-circuit voltage (1.31 V; Supplementary Fig. 41), higher maximum peak power density (139.5 mW cm^−2^ versus 84.8 mW cm^−2^), and greater capacity (787 mAh g^−1^ versus 753 mAh g^−1^) (Fig. 6B, C). To further illustrate the practical potential of H-COP-Co, a light-emitting diode scroller was successfully powered by the ZAB, showcasing its applicability in energy storage devices (Supplementary Fig. 42). Notably, the excellent oxygen electrocatalysis stability of H-COP-Co contributed to the device’s long-term durability during galvanostatic cycling. Specifically, the ZAB with the H-COP-Co cathode operated for 70 h with negligible performance decay, outperforming the ZAB with a Pt/C + RuO_2_ cathode, which maintained operation for only 15 h (Fig. 6D). Moreover, we have compared with many advanced polymer-based ZAB performances and found that the H-COP-Co was excellent in them (Supplementary Table 8).

## Discussion

In summary, we demonstrated a template-source strategy for creating catalytic sites in COPs, successfully synthesizing nanometer-scale COPs with hollow structures containing high-density active sites. Physical characterization elucidated the formation mechanism of H-COP-Co and identified the electronic states and local structure of Co atoms. H-COP-Co exhibited superior oxygen electrocatalytic performance compared to S-COP-Co. Comprehensive in situ spectroscopic analysis revealed that the high density of Co atoms tended to aggregate into Co oxides. These findings underscore the potential of well-designed composite COP materials to manipulate intrinsic reaction mechanisms, offering a transformative approach to revolutionizing catalytic processes.

## Methods

### Materials and characterization

2-Methylimidazole (≥99.5%, Aladdin), 1,3,5-Benzenetricarboxaldehyde,2,4,6-trihydroxy (TP) (>98%, Tensos Biotech), [2,2’- bipyridine]-5,5’-diamine (BPY) (>98%, Tensos Biotech), cobalt nitrate hexahydrate (AR), methanol (MeOH, AR), tetrahydrofuran (THF, AR), cobalt acetate (≥99.5%), ethanol (EtOH, AR), 5 wt.% Nafion, and potassium hydroxide (>90%, KOH, ACS) were from Tansoole.

PXRD data were recorded on an Ultima IV diffractometer with Cu Kα radiation by depositing powder on a glass substrate, from 2θ = ~ 5° up to ~50° or higher angle with a 4° increment. XPS measurements were carried out on a Thermo Scientific K-Alpha XPS spectrometer using an Al Kα X-ray source for radiation. High-resolution transmission electron microscope images were obtained by Transmission electron microscopy (TEM, FEI Tecnai G2) installed with an energy dispersive spectrometer (EDS, Oxford). The metal content in samples was determined by ICP-AES (Jarrel Ash model 955).

### Catalyst preparation

#### Synthesis of ZIF-67

A mixture of Co(NO_3_)_2_·6H_2_O (1.0275 g, 3.5 mmol), 2-Methylimidazole (4.59 g, 0.056 mol), and 50 mL methanol was added and stirred. Subject the mixture to a high-pressure reactor at 110 °C for 24 hours. The ZIF-67 solid was washed with MeOH and THF in sequence and then dried in a vacuum.

#### Synthesis of COP@ZIF

Fetch 100 mg of ZIF-67 and 5.759 mg of BPY (0.03 mmol), and dissolve them in 67 mL of THF. Prepare 4.289 mg of TP (0.02 mmol)and 5 mL of THF mixture to stir with the previous solution for 12 h. The COP@ZIF was obtained after vacuum filtration and drying.

#### Synthesis of H-COP-Co

30 mg COP@ZIF dissolving in 40 mL deionized water, react for 30 min in an 80 °C hot water bath, ~10 mg of H-COP-Co can be obtained after filtration and drying.

#### Synthesis of S-COP-Co

The BPY (57.59 mg, 0.3 mmol) and TP (42.89 mg, 0.2 mmol) were stirred in THF solution for 12 h to obtain Tpbpy. Fetch 90 mg of Tpbpy and 60 mg of cobalt acetate, and dissolve them in 60 mL of MeOH for 4 h. The S-COP-Co solid was washed with MeOH and THF in sequence and then dried in a vacuum.

### XAFS measurements and analysis

#### XAFS measurements

The main XAFS data were collected at the BL20U1 in Shanghai Synchrotron Radiation Facility (SSRF, 3.5 GeV, maximum current of 200 mA)^66^. The beamline is equipped with a fixed-exit Si (111) double-crystal monochromator for metal K-edge collection. The fluorescence data collection uses a Lytle detector. The energy calibration of samples uses the metal foil. In situ cobalt K-edge XAFS experiments were performed with catalyst-coated carbon paper as the WE, reference electrode (RE, Ag/AgCl), and a counter electrode (CE, Pt wire) in an electrochemical cell (Supplementary Fig. 19).

#### Analysis of XAFS data

The collected EXAFS data were analyzed using a standard procedure with the ATHENA module of the IFEFFIT software packages^67^. To determine the valence states of the Co atoms in the samples, the normalized absorption edge energies of reference Co compounds were recorded. A calibration curve was generated by linearly fitting the normalized edge jumps of the reference compounds at an absorbance of 0.5. The Hanning window (*dk* = 1.0 Å⁻¹) was applied to isolate the EXAFS contributions from the distinct coordination shells. Then, quantitative curve fitting was performed in R-space, using the FT k-space range, with the ARTEMIS module of the IFEFFIT package. Regarding the fitting procedure, the amplitude reduction factor S_0_^2^ was fixed to the optimal value derived from fitting the metal foil data. The structural parameters could be either freely varied or fixed.

### In situ attenuated total reflection-infrared spectroscopy

ATR-IR was carried out on a Bruker VERTEX 80 v FT-IR spectrometer (BL06B in SSRF) equipped with an MCT detector cooled with liquid nitrogen. First, the Ge prism was polished with a slurry of 0.3 μm Al_2_O_3_ and sonicated in deionized water. After polishing, the Ge prism was soaked in a piranha solution (3:1 volumetric ratio of 98% H_2_SO_4_ and H_2_O_2_) for 60 min in order to clean the prism of organic contaminants. Then the reflecting surface was immersed in a mixture of the Au plating solution at 60 °C for 10 min. Homogeneous catalyst ink was prepared as in the electrocatalytic tests. Then, the catalyst ink was cast onto the Au film modified Ge prism reflecting surface. In situ ATR-IR spectra were recorded during different potentials applied to the WE.

### Computational methods

All DFT calculations were carried out by the Vienna ab initio Simulation Package^68–70^. The frozen-core projector augmented-wave potentials were used to describe the core and valence electronic interactions^71^. The exchange-correlation interaction was described by the generalized gradient approximation (GGA) exchange-correlation functional, treated with the Perdew–Burke–Erzenhoff parametrization of the GGA functional^72–74^. According to the active sites, the models were constructed for theoretical calculations. The lattice length in z direction was set to be 25 Å to ensure adequate vacuum thickness between the slabs. The cutoff energy of the plane wave was set as 400 eV. In addition, the DFT-D3 method was selected to evaluate van der Waals^75^. The first Brillouin zone was sampled using the Gamma k-point mesh of 2 × 2 × 1. The criterion of energy convergence was 1 × 10^−4^ eV. The internal coordinates of each system were fully optimized until the residual Hellmann−Feynman forces were smaller than 0.01 eV/Å per atom. The reaction Gibbs free energy (ΔG) can be obtained by following the equation:

$$\Delta {{\rm{G}}}=\Delta {{\rm{E}}}+{\Delta {{\rm{E}}}}_{{\rm{ZPE}}}-{{\rm{T}}}\Delta {{\rm{S}}}$$

where E and $\,{{\mbox{E}}}_{{\mbox{ZPE}}}$ were the electronic potential energy and the zero-point energy, respectively. T is 298.15 K, and S is the entropy.

### OER measurements

An electrochemical workstation of CHI-760E connected with a typical three-electrode cell to execute the electrochemical measurements at room temperature. In which an Ag/AgCl (3 M KCl) is used as RE, and the Pt wire is used as CE. To prepare an ink, a 5 mg sample was dispersed in an ethanol (0.25 wt.% Nafion, 500 μL) solution over 0.5 h using ultrasonics. The uniform ink (15 μL) was dropped and dried onto a 5.00 mm diameter glassy carbon electrode (Pine Research Instrumentation, USA) with a loading amount of 0.77 mg cm^–2^. The prepared electrode was used as WE to test the OER performance in N_2_-saturated 1 M KOH (the solution was prepared when using, pH = 13.60 ± 0.11). All Co atoms in the samples were assumed to act as active sites. Based on the Co mass content (determined by ICP-AES), the mass activity and TOF were calculated as follows:

$${{\rm{Mass}}}\; {{\rm{activity}}}=\frac{J * A}{m}$$

$${{\rm{TOF}}}=\frac{J * A}{4 * M * F}$$

Where *J* denotes the current density, *A* represents the WE surface area, m is the mass of the active metal loaded, 4 stands for the electrons transferer number during the ORR or OER, *M* refers to the moles of catalytic sites, and *F* means the Faraday constant (96,485 C mol^−1^).

The convert potentials from the Ag/AgCl scale to the RHE:

$${{\rm{E}}}({{\rm{vs}}}.{{\rm{RHE}}})={{\rm{E}}}({{\rm{vs}}}.{{\rm{Ag}}}/{{\rm{AgCl}}})+0.197+0.059\times {{\rm{pH}}}$$

The pH value is used for the theoretical value under ORR and OER conditions.

CV curves were recorded within the non‑Faradaic potential range under an inert gas atmosphere. The electric double‑layer capacitance (*C*_dl_) was determined from half the current density difference (Δ*J*/2) at the midpoint of the potential window for various scan rates.

Chronoamperometry long-term measurements were performed for catalysts with a loading of 1 mg, using a piece of carbon paper (*S* = 1 cm^2^).

### ORR measurements

The WE for assessing the ORR performance of various catalysts was based on a RRDE (produced by Pine Research Instrumentation (USA)), featuring a platinum ring and glassy carbon disk. Electrochemical measurements were performed in an oxygen-saturated 0.1 M KOH aqueous solution (freshly prepared, pH = 12.86 ± 0.02) for the ORR. The RRDE tests were carried out at 1600 rpm rotating speed with a sweep rate of 10 mV s^−1^. Based on the currents recorded at the ring and disk, the electron-transfer number (*n*) and the selectivity of the catalysts (H₂O₂ yield, H₂O₂ (%)) were obtained as follows:

$${{\rm{n}}}=\frac{4 * {{\rm{I}}}_{D}}{({{\rm{I}}}_{R}/{{\rm{N}}})+{{\rm{I}}}_{D}}$$

$${{\rm{H}}}_{2}{{\rm{O}}}_{2}(\%)=\frac{200 * \left({{\rm{I}}}_{R}/{{\rm{N}}}\right)}{\left({{\rm{I}}}_{R}/{{\rm{N}}}\right)+{{\rm{I}}}_{D}}$$

where the ring collection efficiency is given by *N* = 0.37, ${{{\rm{I}}}}_{D}$ and ${{{\rm{I}}}}_{R}$ are the disk and ring currents, respectively.

Based on the Tafel equation (*η* = b $*$ log j + a, where j denotes the current density and b represents the Tafel slope), the Tafel slope was obtained by performing a linear fit on the polarization curves.

Using a rotating disk electrode (RDE, Taizhou Deyi Analysis Instrument Co., Ltd., *d* = 4.00 mm, *S* = 0.125 cm^2^), the long-term chronoamperometric measurements for the catalysts were performed with the same loading amount as in the previous tests.

### ZAB measurements

Homemade zinc–air batteries (ZABs) were fabricated using polished zinc foil as the anode and an aqueous electrolyte consisting of 6 M KOH and 0.2 M zinc acetate (freshly prepared prior to use, pH = 14.86 ± 0.03). The air cathode was either H-COP-Co or 20 wt.% Pt/C, dropped onto carbon paper with a loading of 1.0 mg cm^−^^2^. Discharge and charge linear sweep curves were recorded using a CHI-760E workstation. The galvanostatic cycling stability of the ZABs was evaluated at a constant current density (5 mA cm^−^^2^), with alternating 5‑minute discharge and 5‑minute charge cycles under ambient conditions. The specific capacity was also calculated as follows:

$${{\rm{Specific}}}\,{{\rm{capacity}}}=\frac{{{\rm{Current}}}\times {{\rm{Time}}}}{{{\rm{Mass}}}\; {{\rm{of}}}\; {{\rm{consumed}}}\; {{\rm{zinc}}}}$$

## Supplementary information

Supplementary Information

Description of Additional Supplementary Files

Supplementary Data 1

Transparent Peer Review file

## Source data

Source data

## Data Availability

The authors declare that all the data supporting the findings of this study are available within the article. The Supplementary Information, Source Data, and full image dataset are also available from the corresponding author upon request. Source data are provided with this paper.
