# Supplementary material for: Constructing high-density active sites on hollow covalent organic polymers for efficient oxygen electrocatalysis
Source: Nat Commun. 2026 Jun 9;17:7346. doi: 10.1038/s41467-026-73508-z (PMC13402350; doi:10.1038/s41467-026-73508-z)
Supplement: Supplementary file 1 — Supplementary Information [file 41467_2026_73508_MOESM1_ESM.pdf]

Supplementary Information

Constructing High-Density Active Sites on Hollow Covalent Organic Polymers for Efficient Oxygen Electrocatalysis

Fei et al.

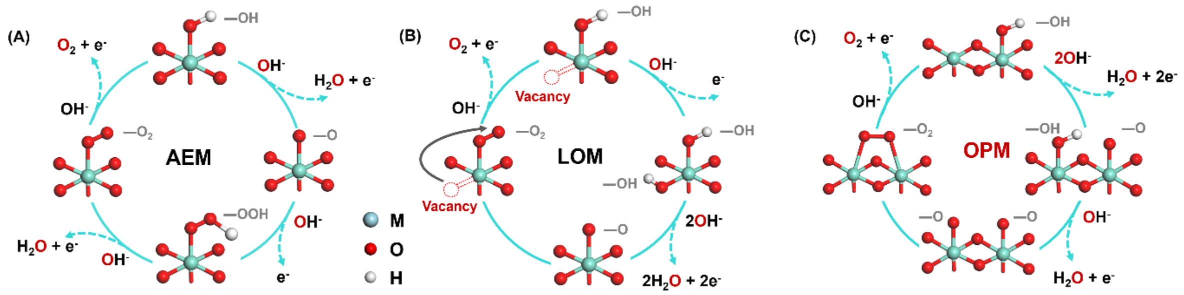

Supplementary Fig. 1. Schematic illustration of simplified OER mechanisms. (A) the AEM, (B) the LOM, and (C) the OPM.

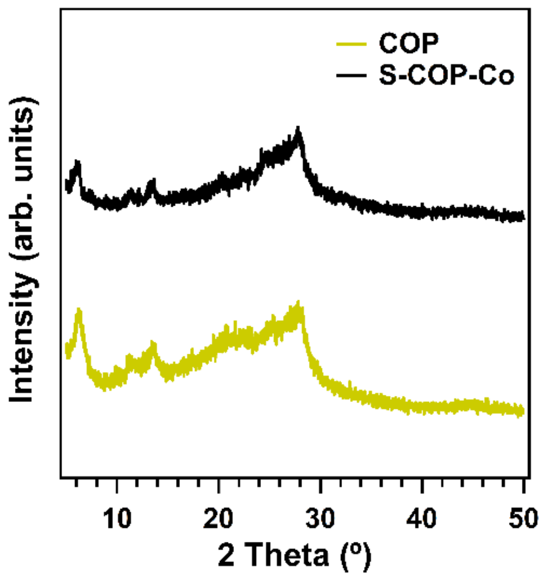

Supplementary Fig. 2. PXRD patterns of COP and S-COP-Co.

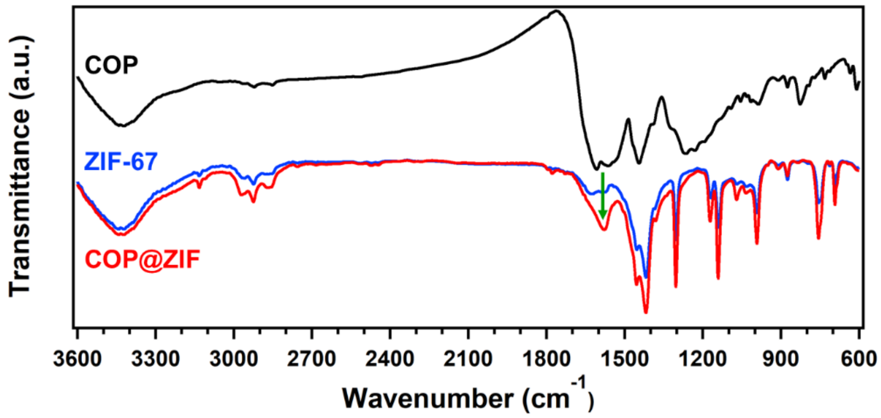

Supplementary Fig. 3. FTIR curves of the COP (black curve), ZIF-67 (blue curve), and COP@ZIF (red curve).

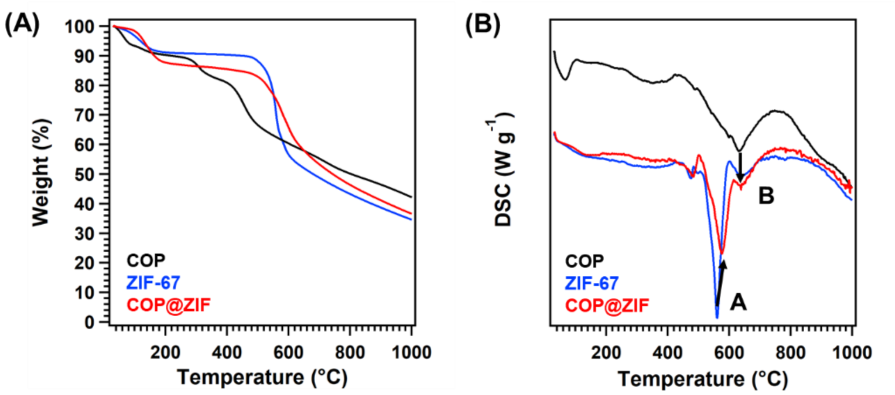

Supplementary Fig. 4. Thermogravimetric analysis. (A) TGA profiles for COP (black curve), ZIF-67 (blue curve), and COP@ZIF (red curve) from room temperature to 1000 °C under N_2. (B) corresponding DSC curve to (A), the COP curve is up-shifted to compare easily.

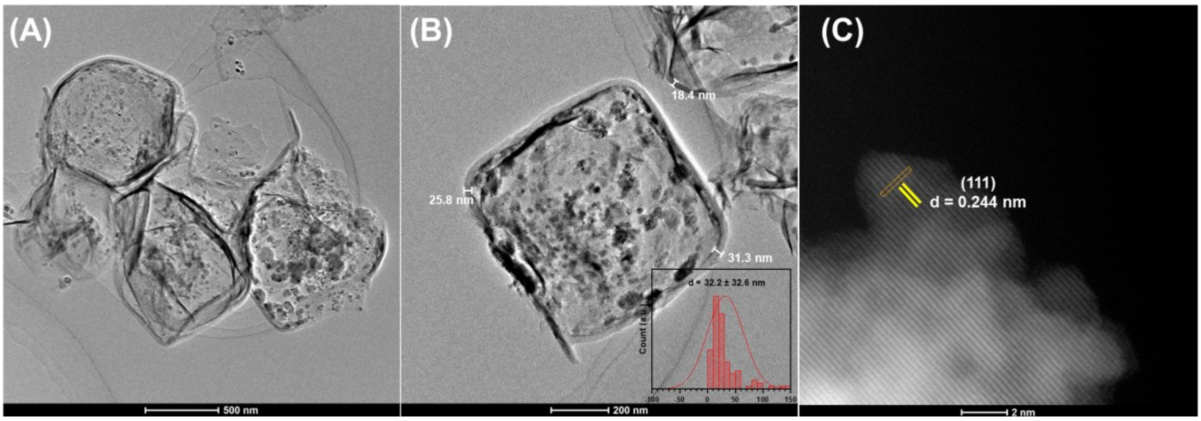

Supplementary Fig. 5. (A), (B) The TEM images of H-COP-Co. Insert pattern in (B): the statistics of the cobalt oxide size. (C) The high resolution TEM image of the H-COP-Co.

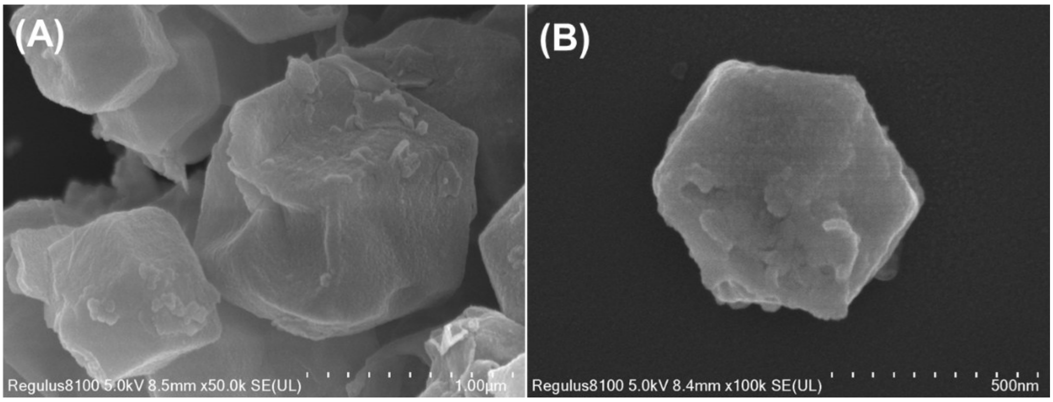

Supplementary Fig 6. SEM images of the H-COP-Co. (A) the scale bar of 1.00 μm, (B) the scale bar of 500 nm.

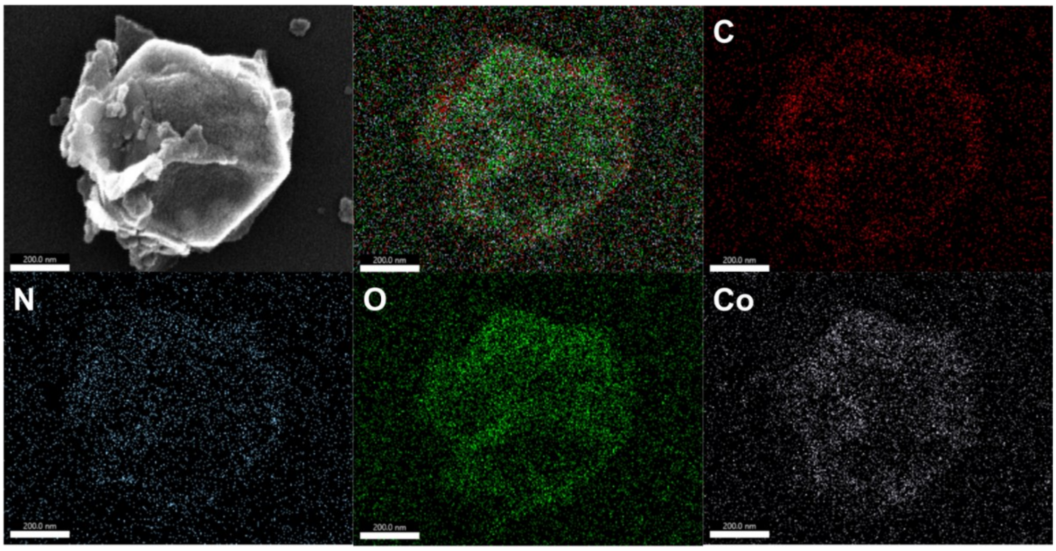

Supplementary Fig. 7. SEM energy dispersive X-ray spectroscopy (EDS) mapping of H-COP-Co.

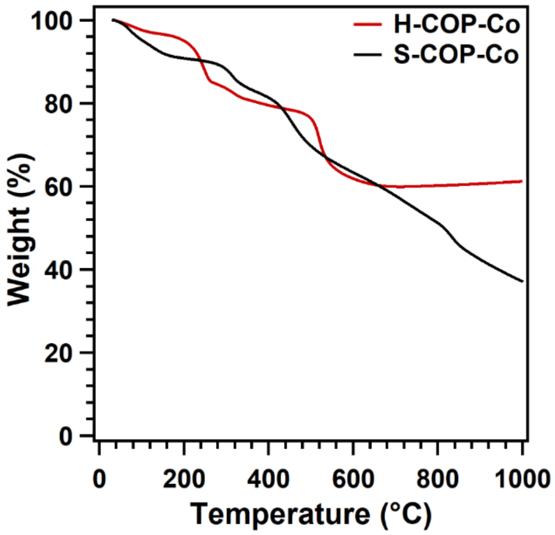

Supplementary Fig. 8. TGA profiles for H-COP-Co (red curve) and S-COP-Co (black curve) from room temperature to 1000 °C under N_2.

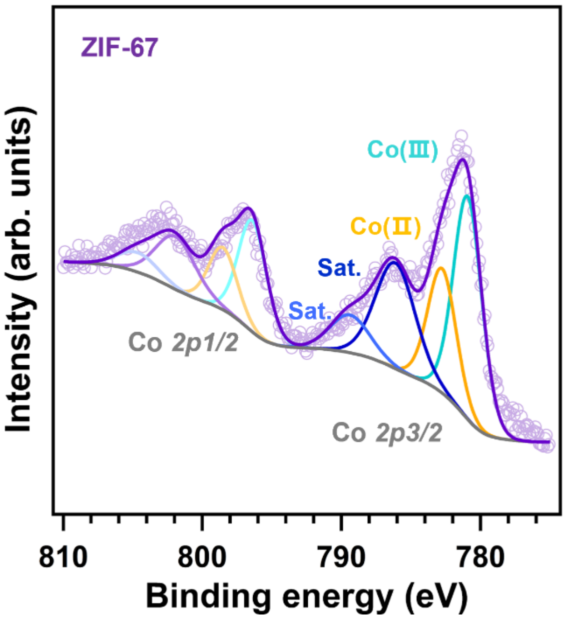

Supplementary Fig. 9. XPS Spectra of Co 2p for ZIF-67.

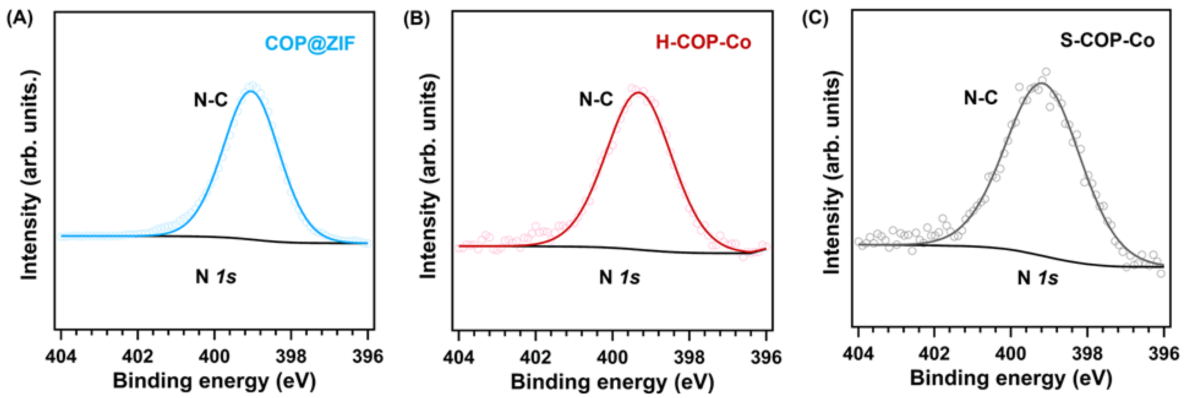

Supplementary Fig. 10. XPS spectra of N 1s. (A) COP@ZIF, (B) H-COP-Co, and (C) S-COP-Co.

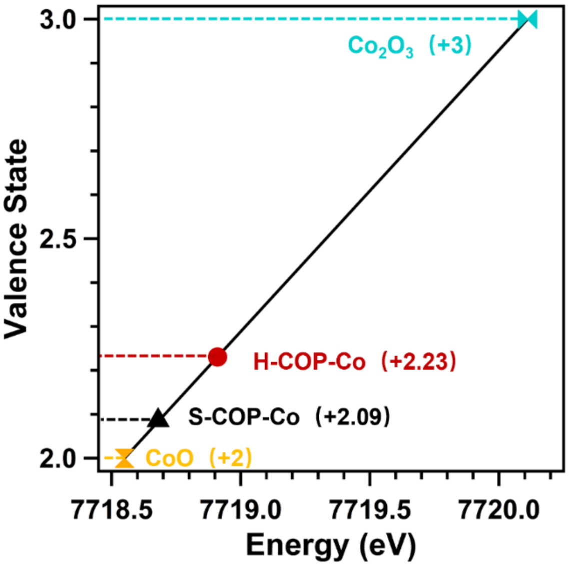

Supplementary Fig. 11. Valence states analysis of Co in different samples.

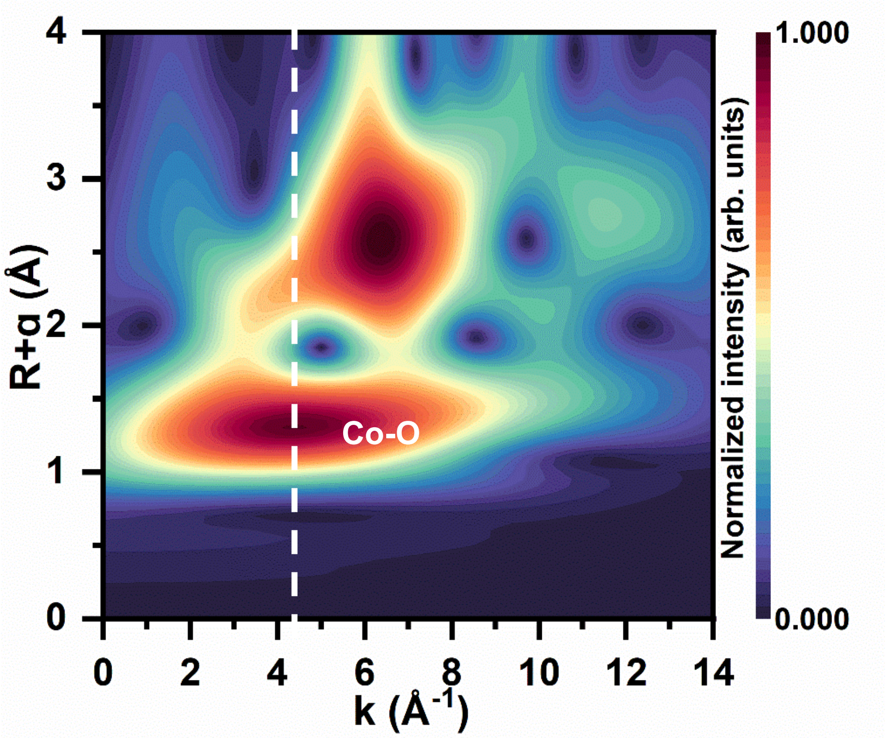

Supplementary Fig. 12. Wavelet transforms contour Co K-edge k^2-weighted EXAFS data spectra for the CoO .

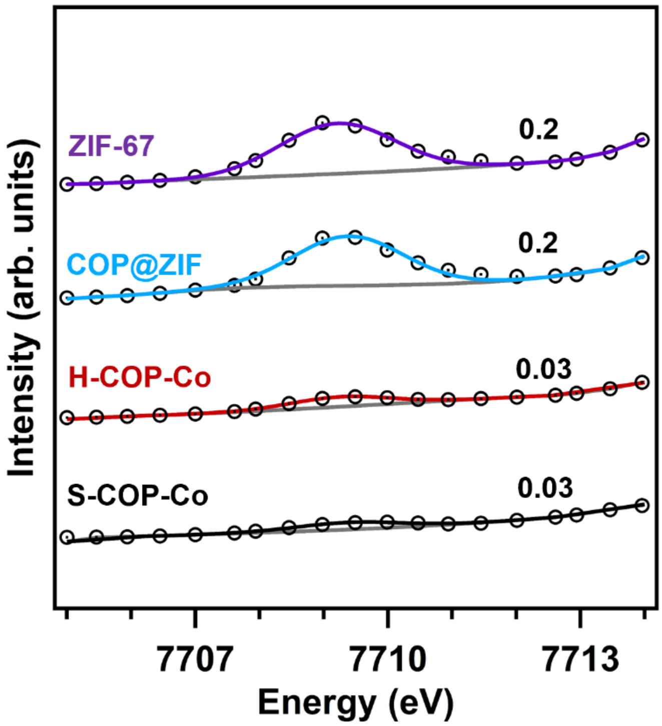

Supplementary Fig. 13. Comparison of pre-edge peaks fitting for different samples.

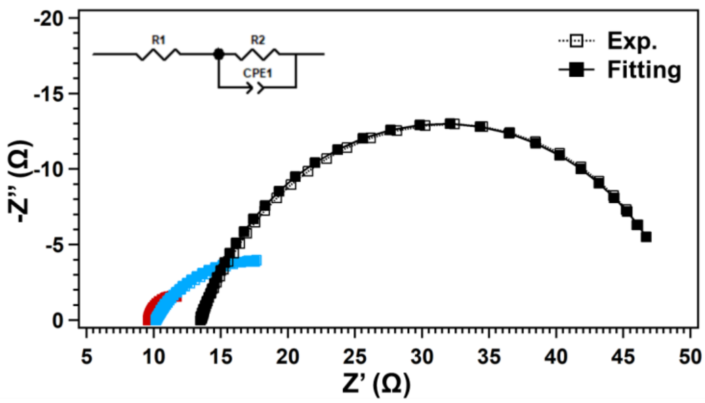

Supplementary Fig. 14. Electrochemical impedance spectroscopy of the H-COP-Co, COP@ZIF, and S-COP-Co catalysts (Inset: equivalent fitted circuit diagram).

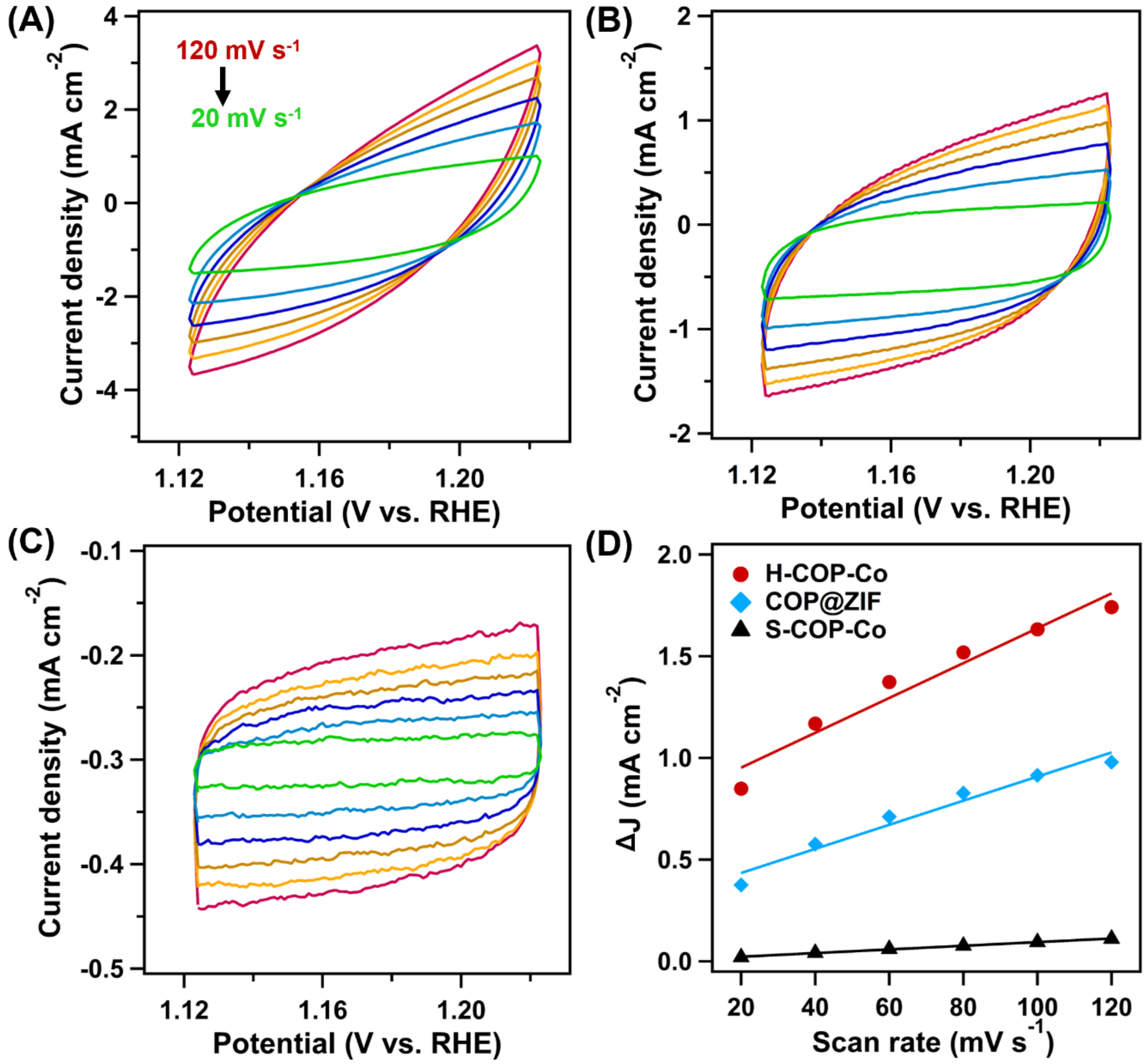

Supplementary Fig. 15. Double-layer capacitance analysis. The CVs of (A) H-COP-Co, (B) COP@ZIF and (C) S-COP-Co in 1 M KOH solution at different scan rates (20, 40, 60, 80, 100 and 120 mV s^{-1}). (D) C_{dl} values for H-COP-Co, COP@ZIF and S-COP-Co.

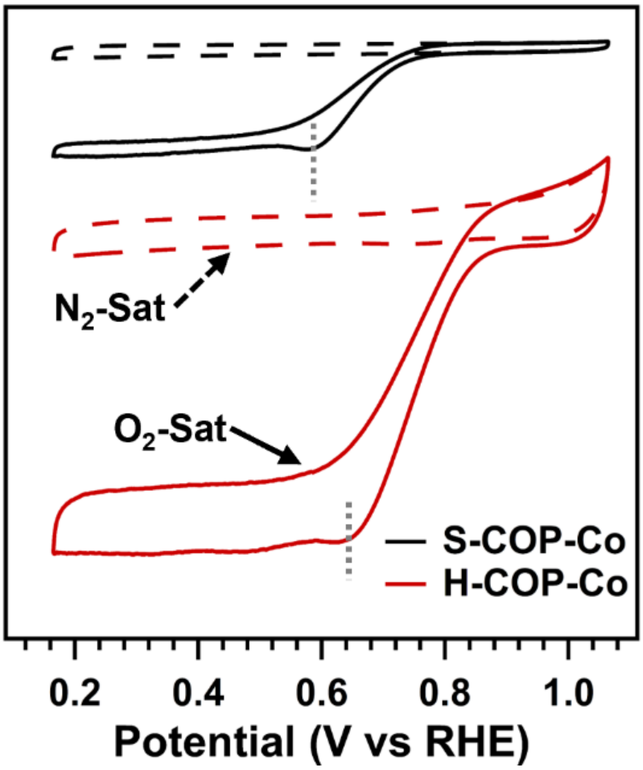

Supplementary Fig. 16. The CV curves of H-COP-Co and S-COP-Co were acquired under O_2-saturated (solid lines) and N_2-saturated (dashed lines) conditions.

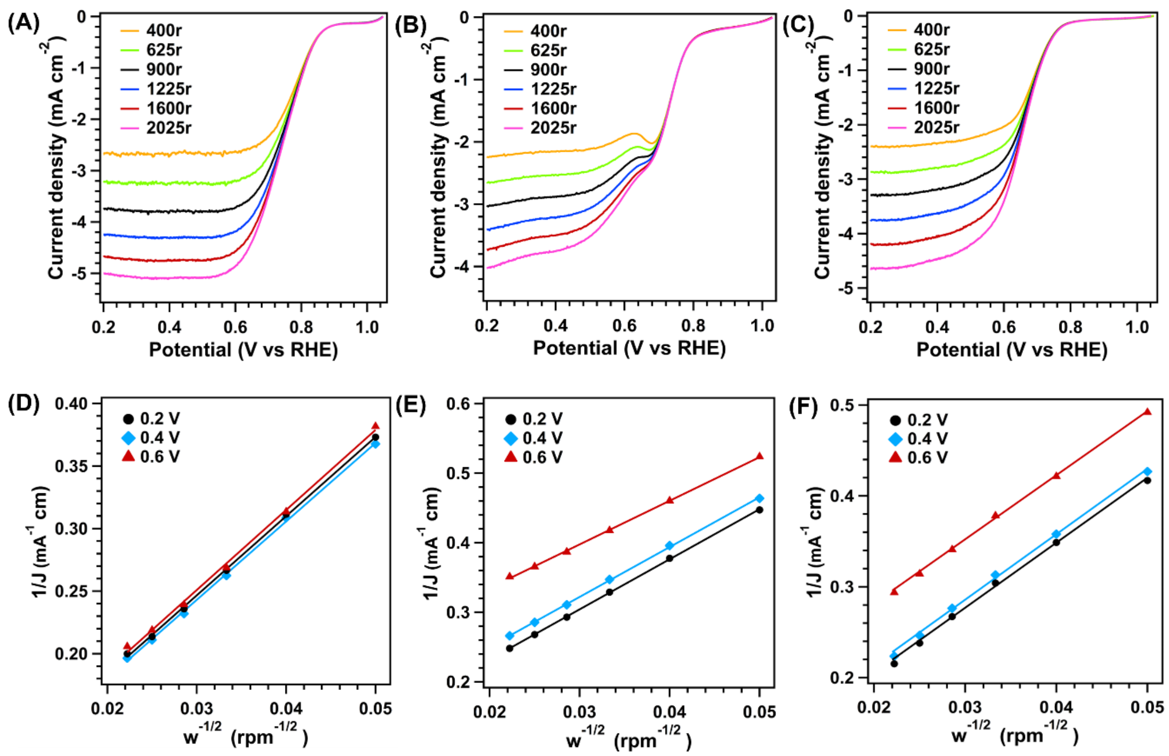

Supplementary Fig. 17. The LSV curves at various rotation speeds (400 - 2025 rpm) of the (A) H-COP-Co, (B) COP@ZIF and (C) S-COP-Co. The K-L plots for (D) H-COP-Co, (E) COP@ZIF and (F) S-COP-Co at 0.6, 0.4 and 0.2 V vs RHE.

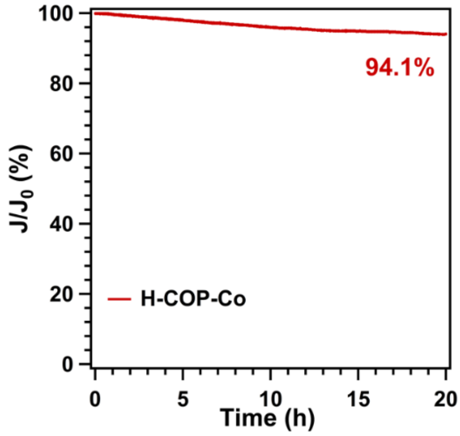

Supplementary Fig. 18. Long-term stability for 20 h of H-COP-Co at 0.2 V versus RHE with 1600 rpm in 0.1 M KOH solution.

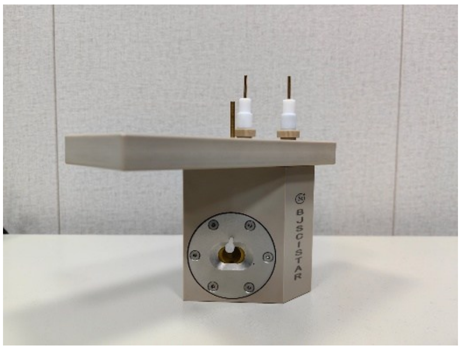

Supplementary Fig. 19. The picture of the in situ XAFS electrochemistry cell.

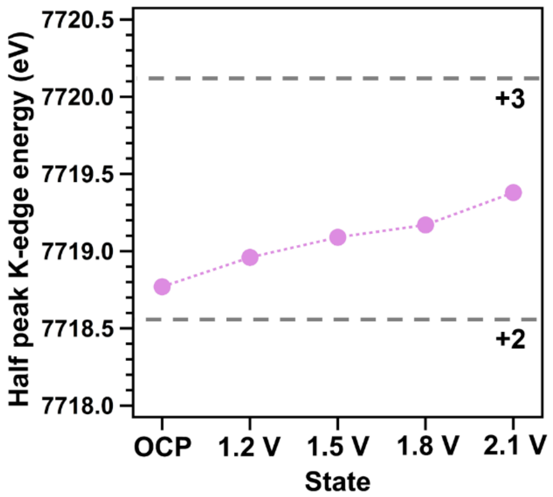

Supplementary Fig. 20. Co valence states analysis of H-COP-Co in OER different states.

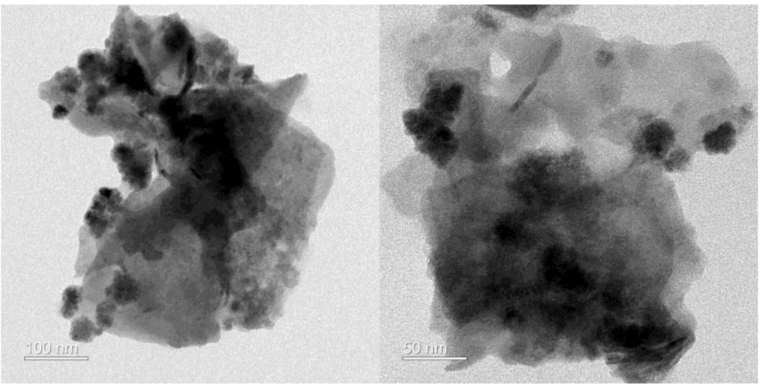

Supplementary Fig. 21. TEM images of the after reaction H-COP-Co.

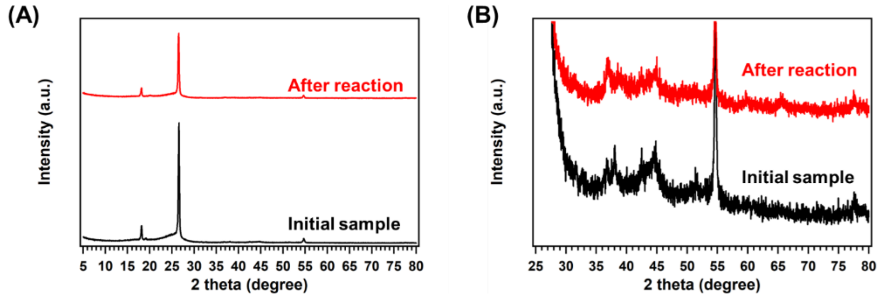

Supplementary Fig. 22. (A) PXRD patterns of the initial (black curve) and after reaction (red curve) sample loaded on the carbon paper. (B) Local enlarged pattern of the (A).

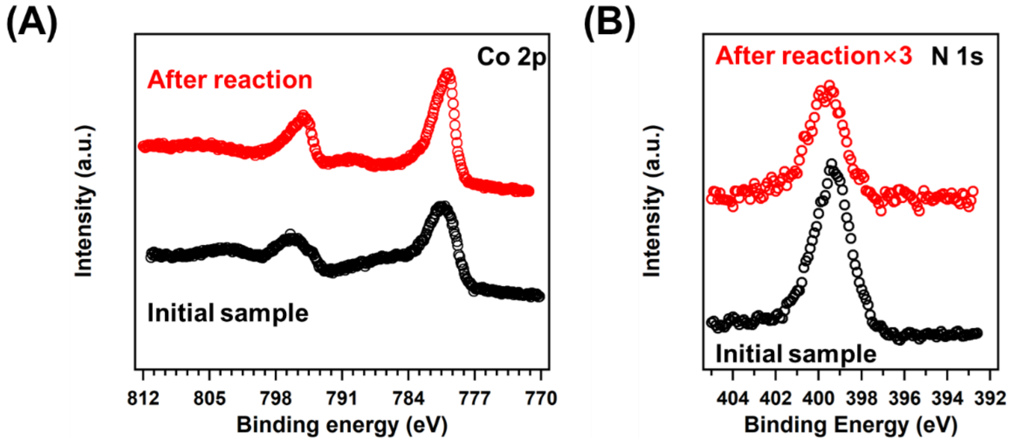

Supplementary Fig. 23. (A) Co 2p XPS spectra of the initial (black curve) and after reaction (red curve) sample. (B) N 1s XPS spectra of the initial (black curve) and after reaction (red curve) sample.

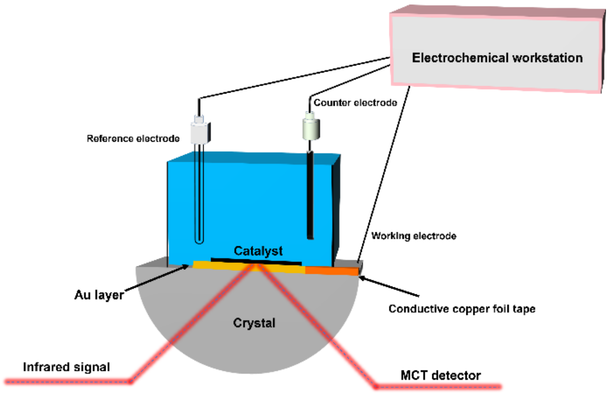

Supplementary Fig. 24. The schematic graph of the in situ electrochemical ATR-FTIR.

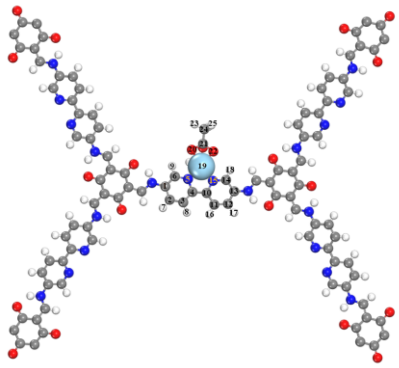

Atomic number	Bader |e|	Atomic number	Bader |e|
1	0.396	14	0.493
2	-0.103	15	-1.131
3	-0.006	16	0.040
4	0.424	17	0.072
5	-1.141	18	0.151
6	0.488	19	1.093
7	0.144	20	-1.073
8	0.105	21	1.484
9	0.139	22	-1.077
10	0.381	23	0.063
11	0.065	24	-0.062
12	0.059	25	0.049
13	0.367		

Supplementary Fig. 25. Charge distribution by Bader charge analysis of the catalytic models for S-COP-Co.

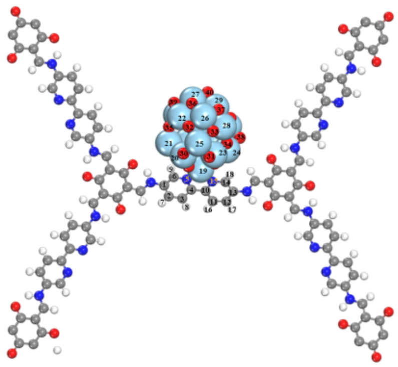

Atomic number	Bader |e|	Atomic number	Bader |e|	Atomic number	Bader |e|
1	0.354	17	0.100	33	-0.846
2	0.045	18	0.177	34	-0.820
3	-0.067	19	1.229	35	-0.874
4	0.428	20	0.992	36	-0.795
5	-1.149	21	0.802	37	-0.797
6	0.504	22	0.986	38	-0.657
7	0.124	23	1.208	39	-0.787
8	0.090	24	1.064	40	-0.653
9	0.209	25	1.299		
10	0.454	26	1.054		
11	0.021	27	1.047		
12	-0.052	28	1.076		
13	0.435	29	1.058		
14	0.508	30	-0.728		
15	-1.121	31	-0.711		
16	0.075	32	-0.878		

Supplementary Fig. 26. Charge distribution by Bader charge analysis of the catalytic models for H-COP-Co.

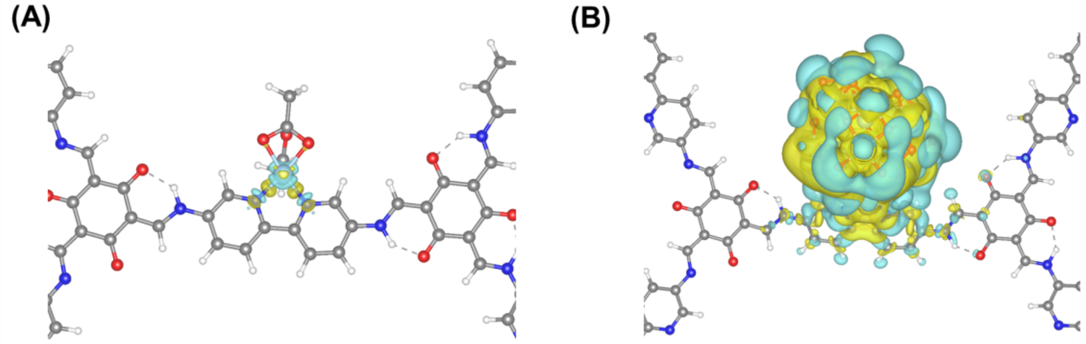

Supplementary Fig. 27. The charge difference density of the catalytic models for (A) S-COP-Co and (B) H-COP-Co, respectively. Pink and green isosurfaces (0.013 $|e|/Å^3$) denote regions of electron accumulation and depletion, respectively. Yellow and blue isosurfaces (0.013 $|e|/Å^3$) denote regions of electron accumulation and depletion, respectively.

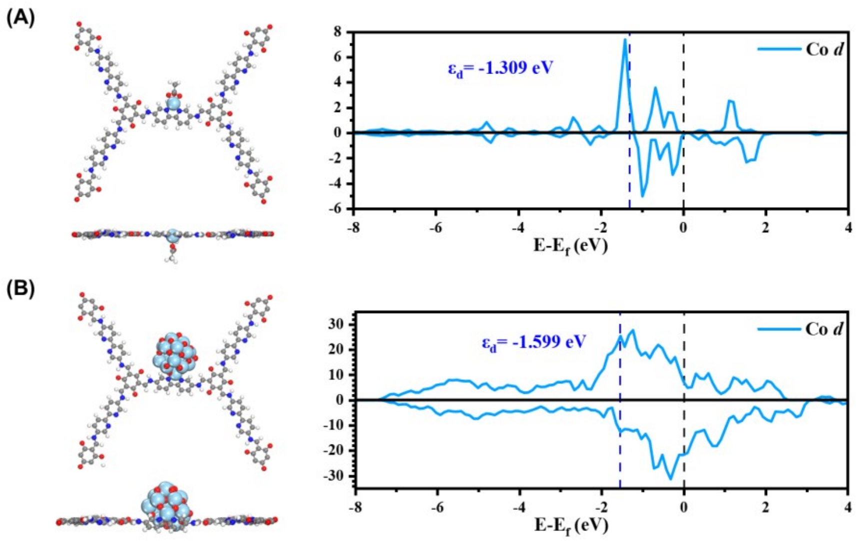

Supplementary Fig. 28. Projected density of states (PDOS) of d orbitals for Co in the catalytic models for (A) S-COP-Co and (B) H-COP-Co, respectively. The Fermi level (E_f) is aligned at 0.00 eV. The d-band center positions are marked by the vertical dashed line labeled by ε_d.

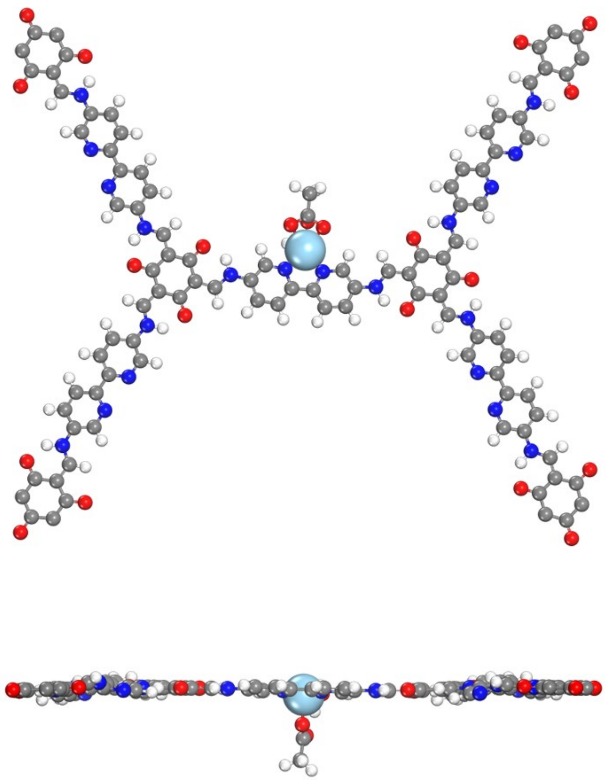

Supplementary Fig. 29. The optimized configuration of S-COP-Co.

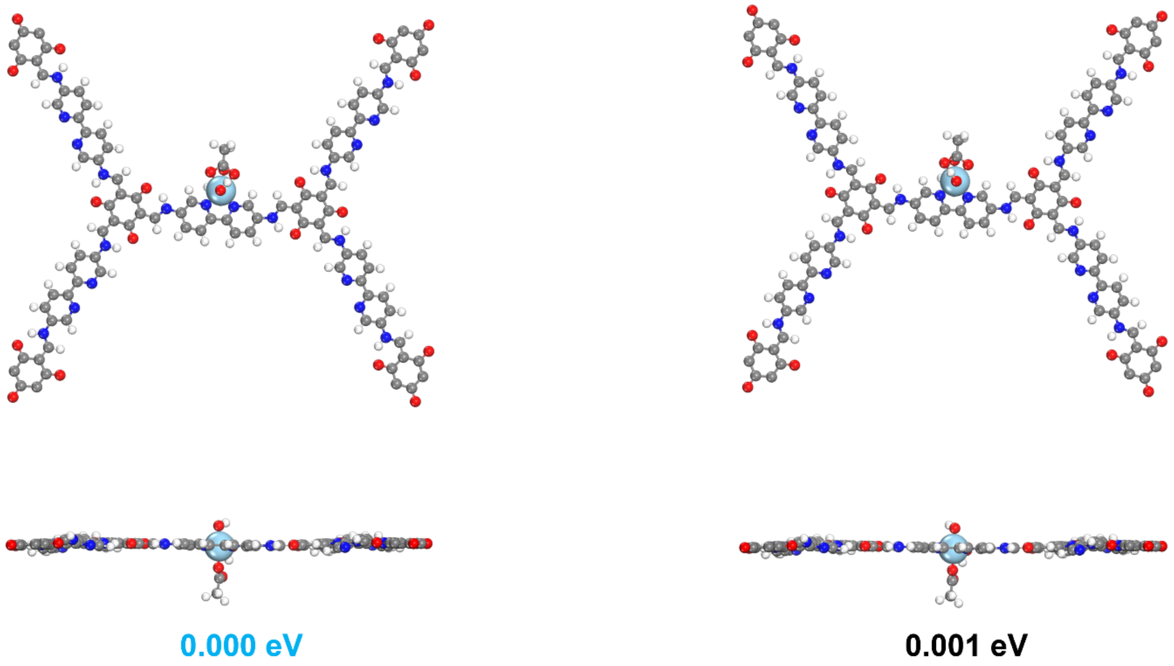

Supplementary Fig. 30. Optimized adsorption configurations of *OH on S-COP-Co surface and their relative energies.

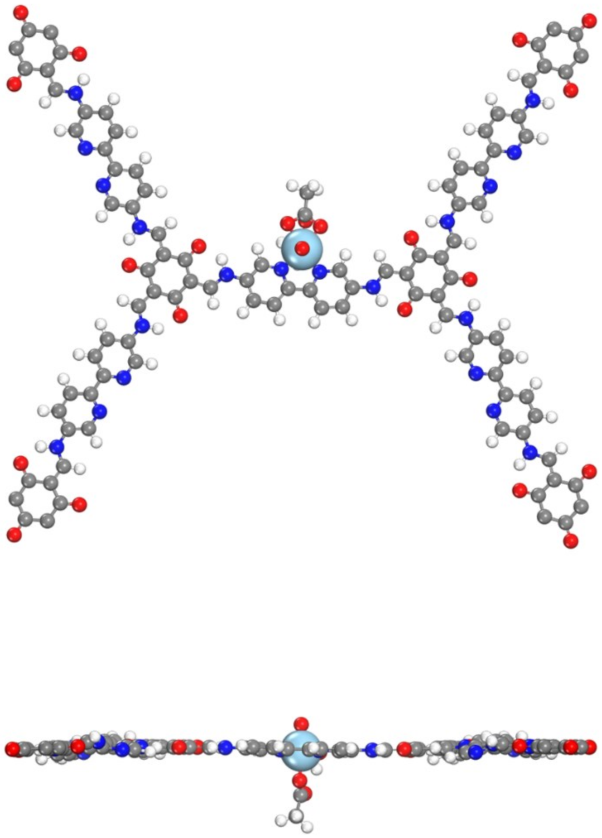

Supplementary Fig. 31. The optimized adsorption configuration of *O on Co-SAC surface.

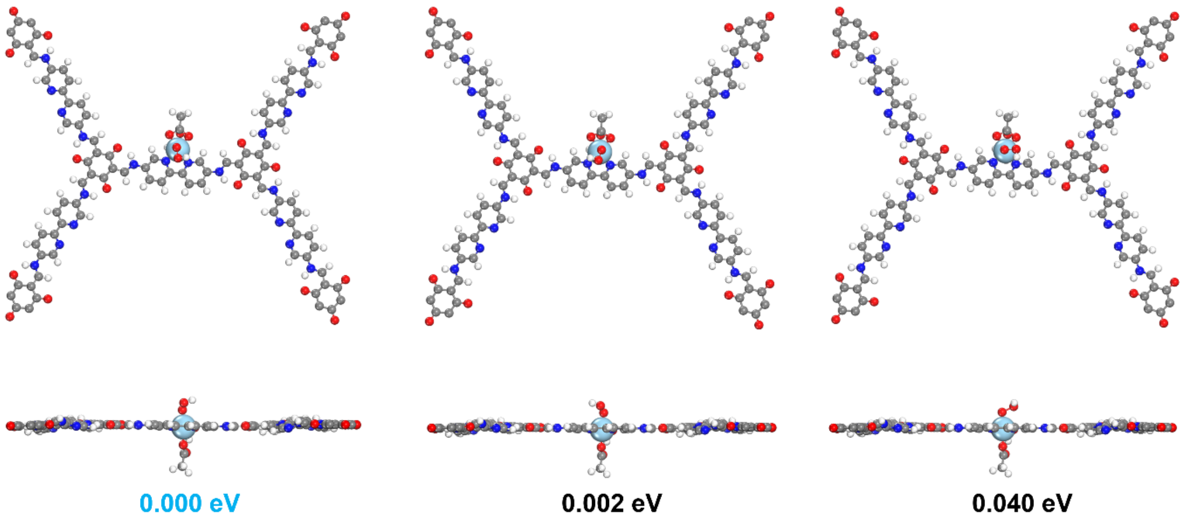

Supplementary Fig. 32. Optimized adsorption configurations of *OOH on Co-SAC surface and their relative energies.

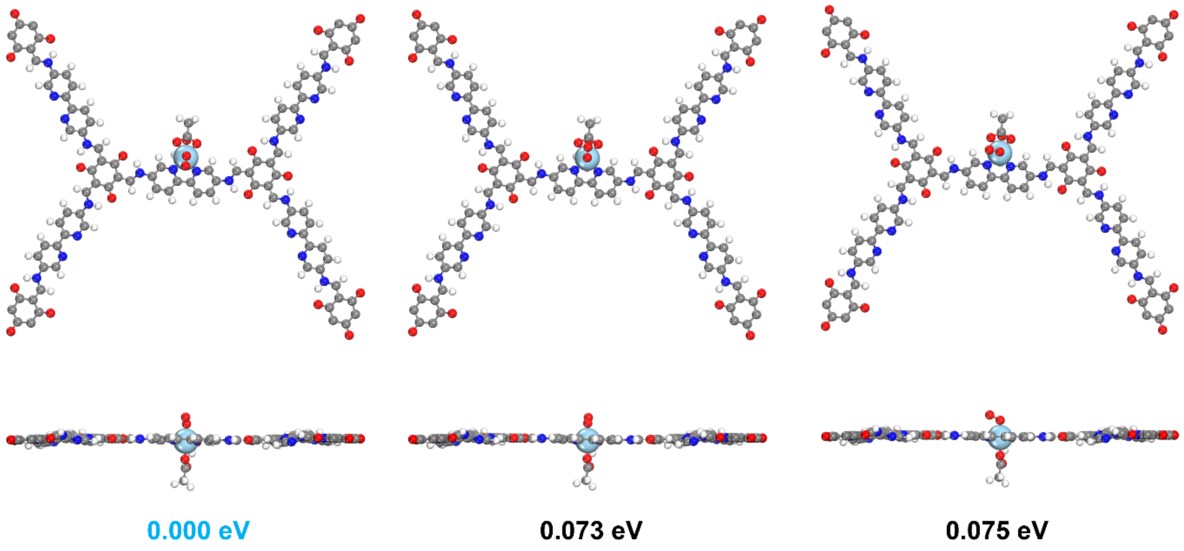

Supplementary Fig. 33. Optimized adsorption configurations of $*O_2$ on Co-SAC surface and their relative energies.

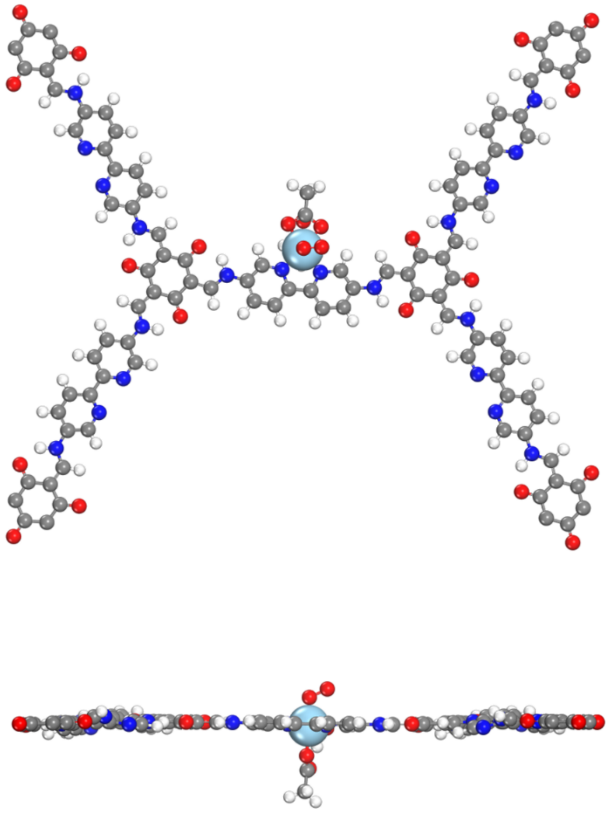

0.077 eV

Supplementary Fig. 34. Optimized adsorption configurations of $*O_2$ on S-COP-Co surface and their relative energies.

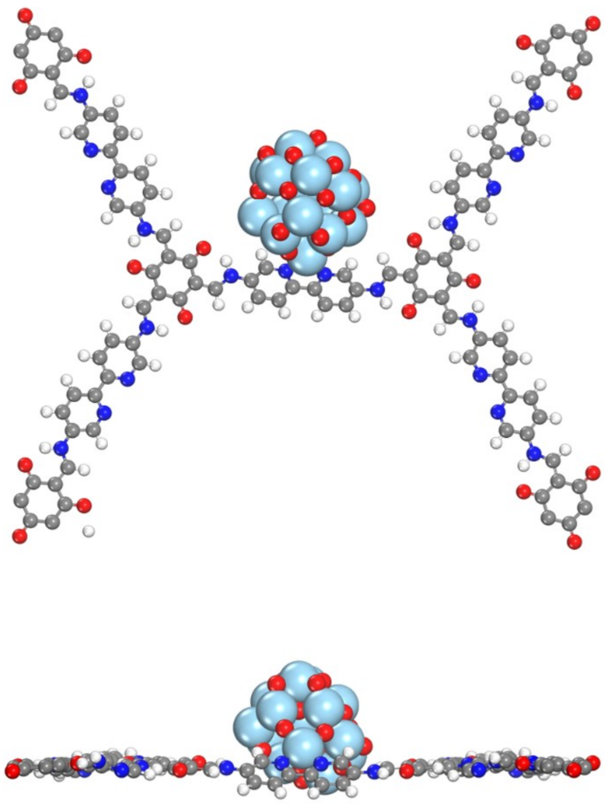

Supplementary Fig. 35. The optimized configuration of H-COP-Co.

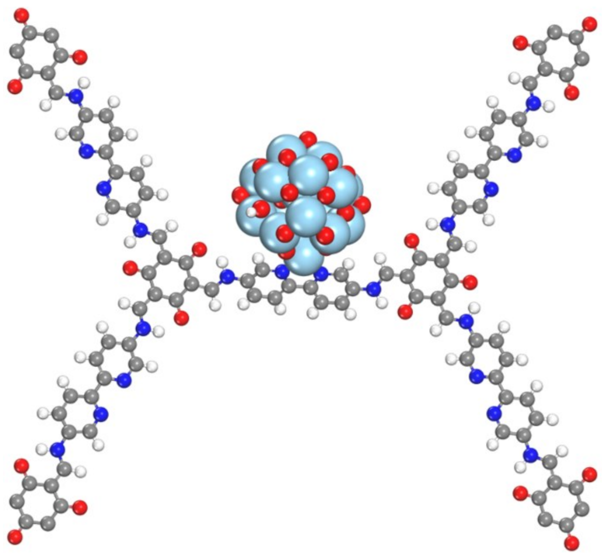

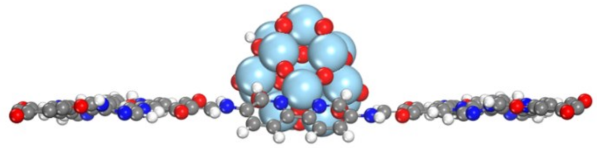

Supplementary Fig. 36. The optimized adsorption configuration of *OH on H-COP-Co surface.

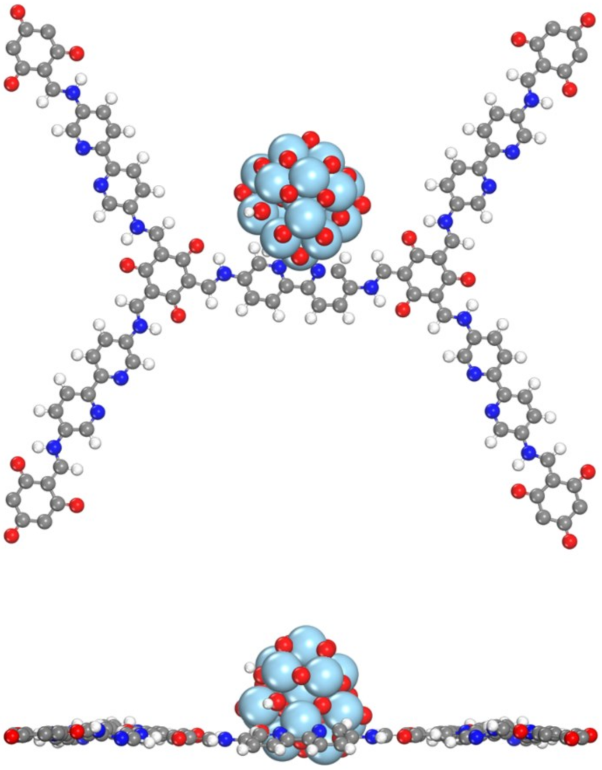

Supplementary Fig. 37. The optimized adsorption configuration of *OH-*OH on H-COP-Co surface.

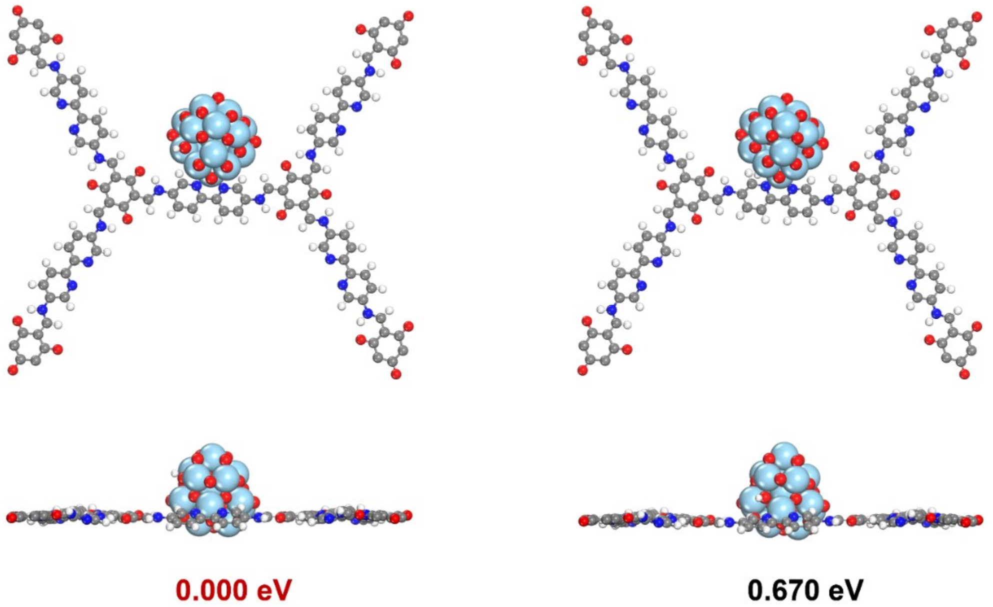

Supplementary Fig. 38. Optimized adsorption configurations of *O-*OH on H-COP-Co surface and their relative energies.

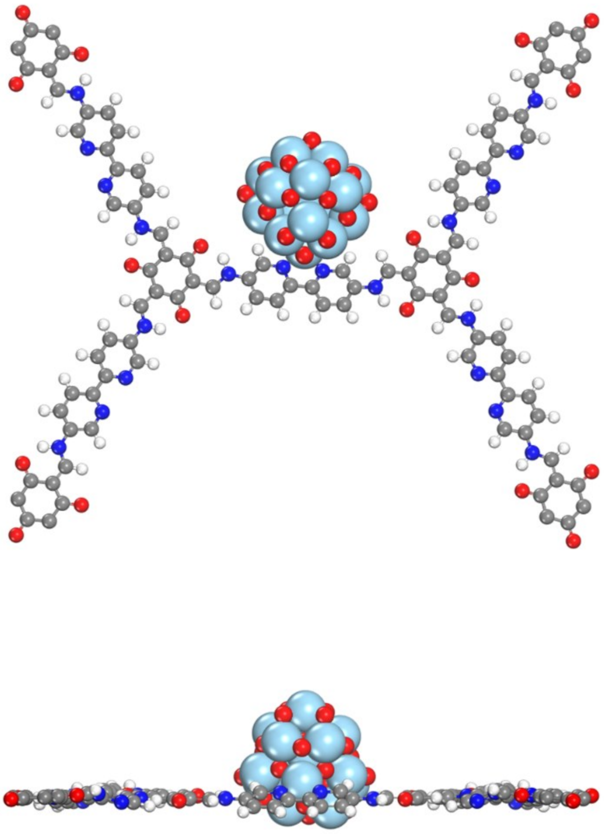

Supplementary Fig. 39. The optimized adsorption configuration of *O-*O on H-COP-Co surface.

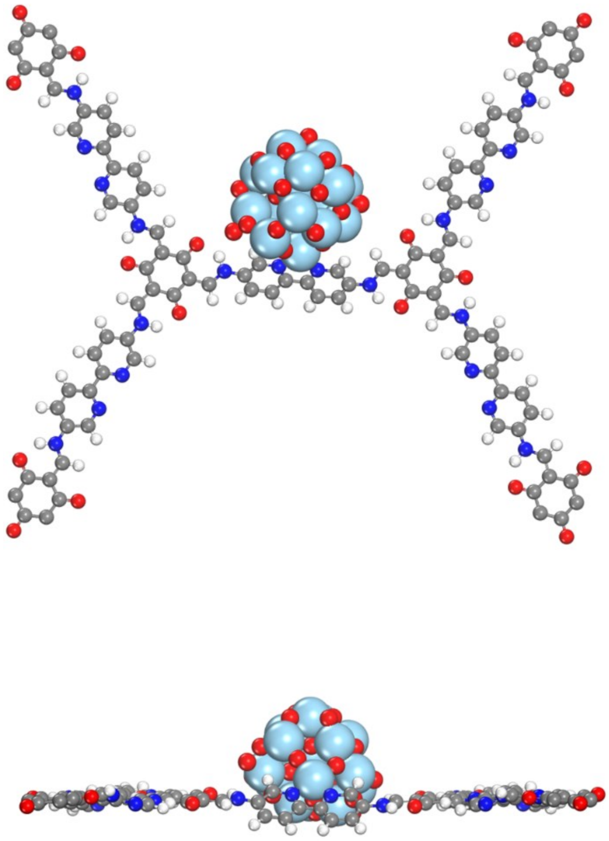

Supplementary Fig. 40. The optimized adsorption configuration of *O_2 on H-COP-Co surface.

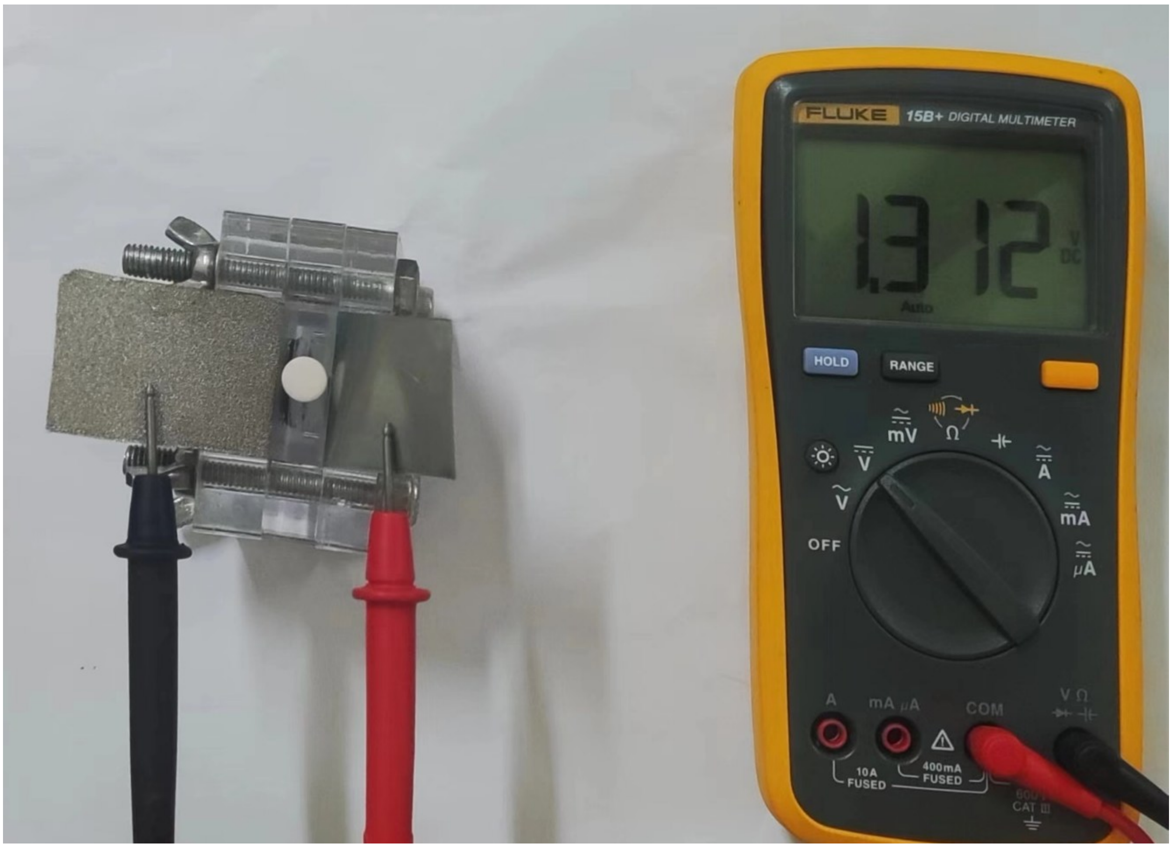

Supplementary Fig. 41. Photograph showing the open-circuit voltage being measured with a multimeter.

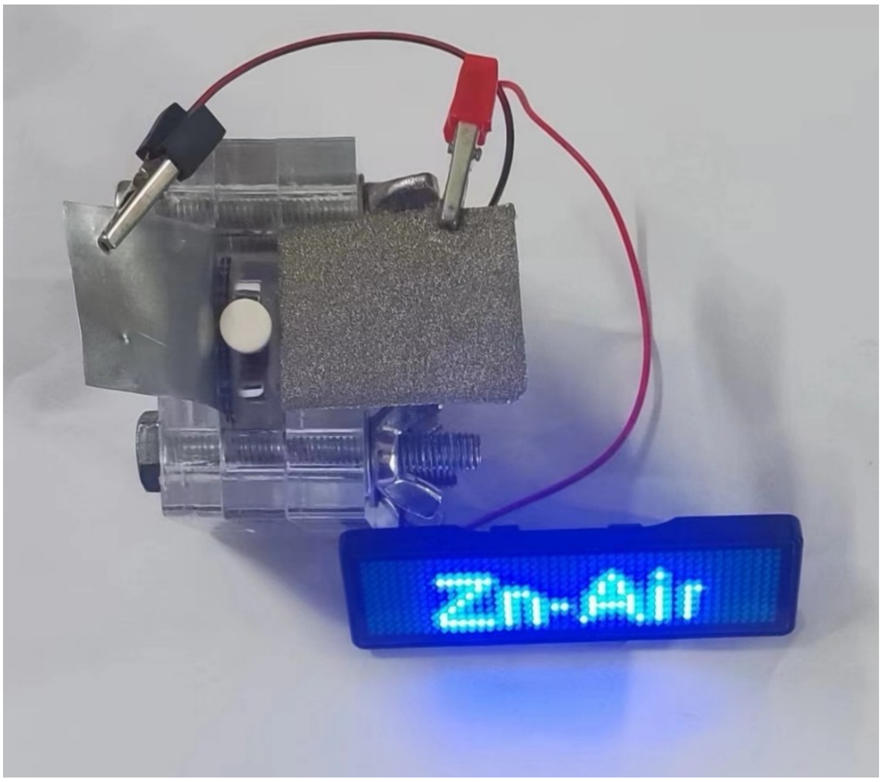

Supplementary Fig. 42. Optical image of an LED powered by H-COP-Co-based ZABs.

Supplementary Table 1. The approximating report of XPS test.

Sample	Elements	Weight (wt%)
COP@ZIF	C	55.1
	N	13.21
	O	15.1
	Co	16.59
H-COP-Co	C	15.56
	N	1.84
	O	32.96
	Co	49.64
S-COP-Co	C	68.04
	N	8.86
	O	16.76
	Co	6.34

Supplementary Table 2. The report of ICP test.

Sample	Elements	Weight (wt%)
COP@ZIF	Co	25.43
H-COP-Co	Co	56.86
S-COP-Co	Co	2.96
After reaction H-COP-Co	Co	29.94

Supplementary Table 3. Co K-edge fitting parameters using the ARTEMIS module of IFEFFIT.

Sample	Shell	C.N.[a]	R (Å) [b]	σ^2 ($\times10^{-3}$Å^2) [c]	ΔE_0 (eV) [d]	R-factor
Co foil	Co-Co	12	2.50±0.00	6.3±0.2	8.8±0.3	0.0008
ZIF-67	Co-N	4.0±0.3	2.01±0.07	3.0	5.2±1.1	0.008
COP@ZIF	Co-N	3.9±0.4	1.98±0.01	3.0	1.7	0.008
	Co-N/O_1	2.8±0.3	2.06±0.03	3.5		
H-COP-Co	Co-N/O_2	1.2±0.2	2.12±0.02	3.5	1.7	0.02
	Co-Co	1.0±0.2	2.85±0.01	3.5		
	Co-N/O_1	2.5±0.1	1.97±0.01	6.1±1.0		
S-COP-Co					2.8±0.9	0.007
	Co-N/O_2	2.0	2.13±0.01	3.0		

[a)], coordination numbers; [b)], the internal atomic distance; [c)], Debye-Waller factor; [d)], the edge-energy shift.

Supplementary Table 4. Summary of recently reported OER and ORR performances of other polymer catalysts under alkaline conditions.

Sample	Electrode	OER		ORR		Reference
		Electrolyte (KOH)	E_{J10} (V vs RHE)	Electrolyte (KOH)	$E_{1/2}$ (V vs RHE)	
H-COP-Co	GC	1 M	1.530	0.1 M	0.753	This work
COP@ZIF	GC	1 M	1.642	0.1 M	0.712	This work
S-COP-Co	GC	1 M	1.828	0.1 M	0.655	This work
CoNi−SAs/NC	GC	1M	1.57	0.1M	0.76	[1]
Co TPP/CNT	GC	1 M	1.637	0.1 M	0.81	[2]
Fe_1Co_1-CNF	GC	0.1 M	1.73	0.1 M	0.87	[3]
Co-POP/C	GC	0.1 M	1.570	0.1 M	0.87	[4]
CoTAPP-PATA-COF	GC	0.1 M	1.65	0.1 M	0.801	[5]
COP_{BTC}-Co	CF	1 M	1.627	1 M	0.864	[6]
CNT-1-Co	GC	1 M	1.67	-	-	[7]
Vo-Cubic-Co_3O_4	CP	1 M	1.605	-	-	[8]
Co_4-Co-MOF	GC	1 M	1.587	0.1 M	0.83	[9]
NiBDC-5	GC	1 M	1.646	-	-	[10]

Supplementary Table 5. Co K-edge of H-COP-Co in OER fitting parameters using the ARTEMIS module of IFEFFIT.

Sample state	Shell	C.N.[a]	R (Å) [b]	σ^2 ($\times10^{-3}$Å^2) [c]	ΔE_0 (eV) [d]	R-factor
OCP	Co-N/O_1	4.3 ± 0.5	1.98 ± 0.01	3.0	-1.2± 0.9	0.01
	Co-N/O_2	1.5 ± 0.5	2.20 ± 0.02	3.0		
	Co-Co	1.5 ± 0.3	2.86 ± 0.01	3.0		
1.2 V	Co-N/O_1	4.2 ± 0.3	1.97± 0.01	3.0		
	Co-N/O_2	1.1 ± 0.3	2.17 ± 0.02	3.0		
	Co-Co	2.2± 0.2	2.86± 0.01	3.0		
1.5 V	Co-N/O_1	4.0 ± 0.5	1.96 ± 0.01	3.0		
	Co-N/O_2	1.1 ± 0.5	2.14 ± 0.02	3.0		
	Co-Co	2.3 ± 0.3	2.86 ± 0.01	3.0		
1.8 V	Co-N/O_1	4.4 ± 0.5	1.96 ± 0.01	3.0		
	Co-N/O_2	1.0 ± 0.5	2.16 ± 0.03	3.0		
	Co-Co	2.5 ± 0.3	2.86± 0.01	3.0		
2.1 V	Co-N/O_1	4.8± 0.4	1.95 ± 0.01	3.0		
	Co-N/O_2	0.8 ± 0.4	2.16 ± 0.03	3.0		
	Co-Co	2.9 ± 0.2	2.86± 0.01	3.0		

[a)], coordination numbers; [b)], the internal atomic distance; [c)], Debye-Waller factor; [d)], the edge-energy shift.

Supplementary Table 6. Co K-edge of H-COP-Co in ORR fitting parameters using the ARTEMIS module of IFEFFIT.

Sample state	Shell	C.N.[a]	R (Å) [b]	σ^2 ($\times10^{-3}$Å^2) [c]	ΔE_0 (eV) [d]	R-factor
OCP	Co-N/O_1	3.0 ± 0.5	1.96 ± 0.01	3.0	-2.9±1.3	0.017
	Co-N/O_2	1.7± 0.4	2.12 ± 0.01	3.0		
	Co-Co	0.8 ± 0.2	2.83 ± 0.01	3.0		
1.0 V	Co-N/O_1	3.1± 0.5	1.94± 0.01	3.0		
	Co-N/O_2	1.5 ± 0.4	2.12 ± 0.01	3.0		
	Co-Co	1.1± 0.3	2.81± 0.01	3.0		
0.7 V	Co-N/O_1	3.3 ± 0.5	1.93 ± 0.01	3.0		
	Co-N/O_2	1.2 ± 0.4	2.09 ± 0.01	3.0		
	Co-Co	1.5± 0.2	2.83 ± 0.01	3.0		
0.4 V	Co-N/O_1	3.5± 0.4	1.93 ± 0.01	3.0		
	Co-N/O_2	1.1 ± 0.3	2.08 ± 0.01	3.0		
	Co-Co	1.8 ± 0.2	2.84± 0.01	3.0		

[a]), coordination numbers; [b]), the internal atomic distance; [c]), Debye-Waller factor; [d]), the edge-energy shift.

Supplementary Table 7. XPS content estimation of H-COP-Co loaded on carbon paper before and after reaction.

Elements	Before (wt.%)	After (wt.%)
C	48.70	57.49
N	3.98	0.92
O	24.29	24.61
Co	23.02	16.99

Supplementary Table 8. Summary of recently reported rechargeable Zn-air batteries performances of other advanced polymer catalysts.

Sample	Peak power density (mW cm^{-2})	Specific capacity (mAh g^{-1})	Reference
H-COP-Co	139.5	787	This work
$PcCuO_8$-Co/CNT	94	700	[11]
PCN-226(Co)	133	724	[12]
BHZ-48	148	-	[13]
CC-3	85	714	[14]
Azo-COF	134	~550	[15]
JUC-650	101.5	722.6	[16]
PD-COF-OH	111.6	676	[17]
BDA-COF	101.8	-	[18]
Co-MOF-74-HATP@EC300J	96.6	-	[19]
OH-COF	119.5	854	[20]

Reference

1. Han, X. et al. Atomically Dispersed binary Co-Ni sites in nitrogen-doped hollow carbon Nanocubes for Reversible Oxygen Reduction and Evolution. *Adv. Mater.* **31**, 1905622 (2019).
2. Qin, H. et al. Cobalt porphyrins supported on carbon nanotubes as model catalysts of metal-N4/C sites for oxygen electrocatalysis. *J. Energy Chem.* **53**, 77–81 (2021).
3. Wang, Y. et al. Flexible carbon nanofiber film with diatomic Fe-Co sites for efficient oxygen reduction and evolution reactions in wearable zinc-air batteries. *Nano Energy* **87**, 106147 (2021).
4. Lei, H. et al. Metal-corrole-based porous organic polymers for electrocatalytic oxygen reduction and evolution reactions. *Angew. Chem., Int. Ed.* **61**, e202201104 (2022).
5. Liu, M. et al. Construction of catalytic covalent organic frameworks with redox-active sites for the oxygen reduction and the oxygen evolution reaction. *Angew. Chem., Int. Ed.* **61**, e202213522 (2022).
6. Mi, C. et al. Symmetric electronic structures of active sites to boost bifunctional oxygen electrocatalysis by MN4+4 sites directly from initial covalent organic polymers. *Adv. Funct. Mater.* **33**, 2303235 (2023).
7. Li, X. et al. A one-pot three-in-one synthetic strategy to immobilize cobalt corroles on carbon nanotubes for oxygen electrocatalysis. *Adv. Funct. Mater.* **34**, 2310820 (2024).
8. Chen, X. et al. Facet-dependent lattice oxygen activation on oxygen-defective co3o4 for electrocatalytic oxygen evolution reaction. *ACS Energy Lett.* **9**, 2182–2192 (2024).
9. Liang, Z. et al. Constructing $Co_4(SO_4)_4$ clusters within metal–organic frameworks for efficient oxygen electrocatalysis. *Adv. Mater.* **36**, 2408094 (2024).
10. Wu, P. et al. Exfoliation of metal–organic frameworks to give 2D MOF nanosheets for the electrocatalytic oxygen evolution reaction. *Angew. Chem., Int. Ed.* **63**, e202402969 (2024).

11. Zhong, H. et al. A phthalocyanine-based layered two-dimensional conjugated metal–organic framework as a highly efficient electrocatalyst for the oxygen reduction reaction. *Angew Chem Int Ed.* **58**, 10677–10682 (2019).
12. Cichocka, M. O. et al. A porphyrinic zirconium metal-organic framework for oxygen reduction reaction: tailoring the spacing between active-sites through chain-based inorganic building units. *J. Am. Chem. Soc.* **142**, 15386–15395 (2020).
13. Jiang, Y. et al. d-Orbital steered active sites through ligand editing on heterometal imidazole frameworks for rechargeable zinc-air battery. *Nat Commun.* **11**, 5858 (2020).
14. Liu, C. et al. One-dimensional van der Waals heterostructures as efficient metal-free oxygen electrocatalysts. *ACS Nano* **15**, 3309–3319 (2021).
15. Yan, X., Wang, B., Ren, J., Long, X. & Yang, D. An unsaturated bond strategy to regulate active centers of metal-free covalent organic frameworks for efficient oxygen reduction. *Angew Chem Int Ed.* **61**, e202209583 (2022).
16. Liu, J. et al. Precise modulation of carbon activity sites in metal-free covalent organic frameworks for enhanced oxygen reduction electrocatalysis. *Small* **20**, 2305759 (2024).
17. Zheng, S., Fu, Y., Xu, X., Xu, Q. & Zeng, G. Integrating electronic-storage piperazine into covalent organic frameworks for promoting oxygen reduction reaction. *Angew Chem Int Ed.* **64**, e202503434 (2025).
18. Zhao, G., Zou, X., Ma, H., Wang, L. & Guo, H. Linkage-engineered covalent organic frameworks regulating the electron transfer for promoting oxygen reduction. *J. Energy Chem.* **108**, 83–91 (2025).
19. Liu, W. et al. Ligand engineering of Co-MOF-74 with hexaaminotriphenylene for enhanced oxygen reduction reaction in zinc-air batteries. *Nano Research* **18**, 94907195 (2025).

20. Chen, M. et al. Amplification effect of side group regulation via imidazolate linkages of covalent organic frameworks for efficient oxygen reduction. *Chem. Sci.* **16**, 11669-11677 (2025).
