# Supplementary figures and images for: Constructing high-density active sites on hollow covalent organic polymers for efficient oxygen electrocatalysis
Source: Nat Commun. 2026 Jun 9;17:7346. doi: 10.1038/s41467-026-73508-z (PMC13402350; doi:10.1038/s41467-026-73508-z)

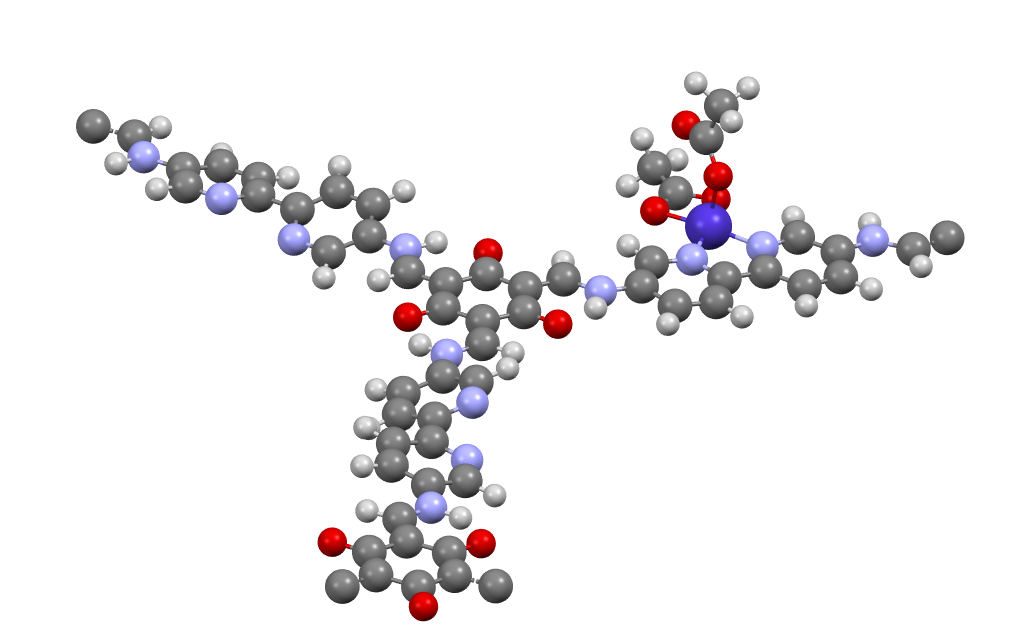


1. Established single Co site model.


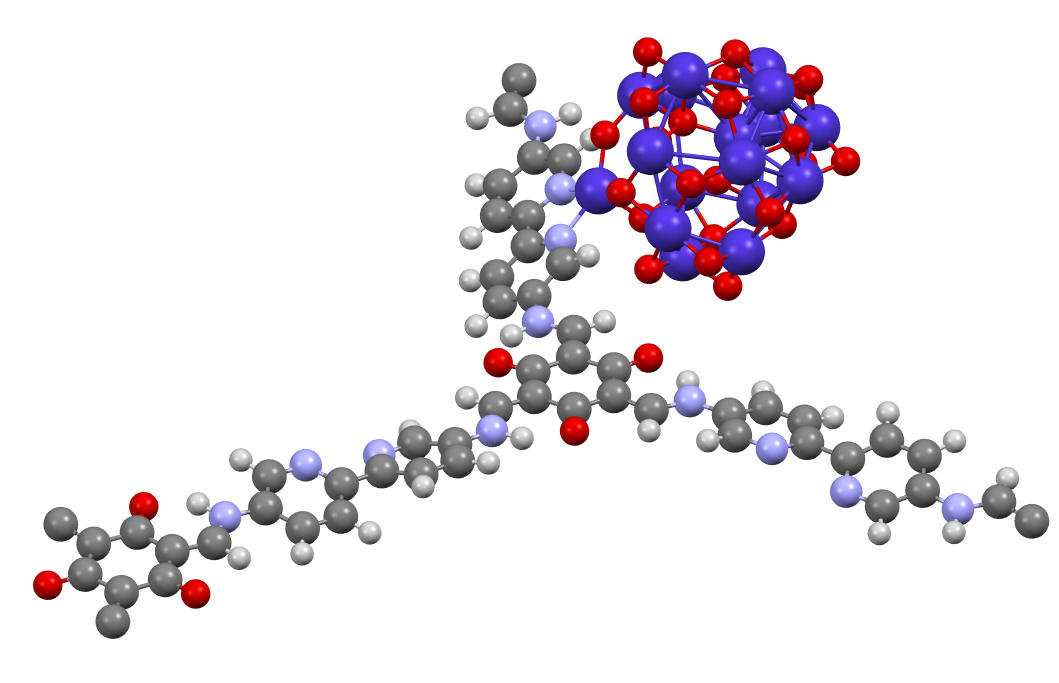


1. Established Co oxide cluster model.

Supplement: Supplementary file 2 — Description of Additional Supplementary Files [file 41467_2026_73508_MOESM2_ESM.docx]
