# Supplementary material for: Constructing high-density active sites on hollow covalent organic polymers for efficient oxygen electrocatalysis
Source: Nat Commun. 2026 Jun 9;17:7346. doi: 10.1038/s41467-026-73508-z (PMC13402350; doi:10.1038/s41467-026-73508-z)
Supplement: Supplementary file 4 — Transparent Peer Review file [file 41467_2026_73508_MOESM4_ESM.pdf]

nature portfolio

Peer Review File

Constructing High-Density Active Sites on Hollow Covalent Organic Polymers for Efficient Oxygen Electrocatalysis

Corresponding Author: Professor Zheng Jiang

This file contains all reviewer reports in order by version, followed by all author rebuttals in order by version.

Attachments originally included by the reviewers as part of their assessment can be found at the end of this file.

Version 0:

Reviewer comments:

Reviewer #1

(Remarks to the Author)
Please find attached.

Reviewer #2

(Remarks to the Author)
The authors reported high-density active sites on hollow covalent organic polymers for oxygen electrocatalysis. Generally, there are much more improvements and clarifications both in the technical execution and in the overall presentation. It does not reach the high standards of Nature Communications, and thus is not suitable to publish in the journal.
1. The H-COP-Co catalyst synthesized by the authors exhibits an OER overpotential of 270 mV and an ORR E1/2 of 0.753 V. However, it is noteworthy that numerous Co-based SACs have already demonstrated OER overpotentials lower than 250 mV and ORR E1/2 higher than 0.90 V. In light of these advancements, could the authors elaborate on the practical significance of oxygen electrocatalysis for the H-COP-Co? Specifically, what unique advantages or improvements do this method offer compared to existing high-performance catalysts?
2. They declared that H-COP-Co demonstrated an increase in active metal content, from 2.96% to 56.86%, however, from the HAADF-STEM images, the density of bright dots is very sparse, even less than those with 1% metal content reported previously (e.g. Nat. Catal. 2020, 3, 1044–1054). Why?
3. The ZAB performance is very poor, which lacks application promising. Could the authors compare it with other SAC-based ZABs with so poor performance?

Reviewer #3

(Remarks to the Author)
Comments
COPs were one of the most promising functional materials in energy fields. How to introduce active species and improve their densities is most desired to develop. This report first demonstrated the novel construction strategy of composited COPs that exceed the traditional post-modification strategies on active species density and performances. The theoretical calculation and in situ spectroscopies were well conducted to reveal the novel efficient OPM pathway on the H-COP-Co. This is significant work in the field of physics and chemistry of polymer materials, and the comprehensive in situ technology and characterization analysis showed a positive influence on the cross-fields. Minor revision is recommended before publication on Nature Communications.

Q1. What is the product of pure ZIF-67 after the hot water disposal?
Q2. The previous understanding of the different mechanisms of oxygen electrocatalysis pathway and chemical equations should be listed and explained.
Q3. The authors discussed the symmetry by XANES pre-edge analysis, and a detailed analysis is suggested.
Q4. In Fig. 3D, the plotting of long-term tests seems not standard, a percentage description is better. The authors should adjust this.

Q5. The TGA tests of the S-COP-Co should be measured to illustrate the difference in the metal contents of the two construction strategies.
Q6. The authors lacked the FTIR spectra data of COPs that were essential to identify whether the polymers were successfully synthesized.
Q7. The stability of COP should be assessed at the hot water conditions.

Reviewer #4

(Remarks to the Author)
Fei et al. demonstrated the template-source construction strategy to synthesize the hollow COPs with highly active Co-O species. Many COPs studies focused on the single metal site design that optimizes traditional AEM pathways, this work first offers novel sights to design polymers and other energy materials through bottom-up methods to modulate the oxygen evolution reaction mechanism. Importantly, this study well implemented the in situ X-ray and infrared spectroscopies to explore the dynamic changes of catalytic sites, which gives us a new inspiration to study the catalytic mechanism. I recommend publication in Nat. Commun. after addressing the following comments:

1) According to the procedure, the Co content of COP@MOF was high. However, the intensity from the XPS signal of COP@MOF is evidently lower than that from the H-COP-Co. The authors should explain the reason.
2) This work highlighted the improvement from the post-modification strategy. Was the S-COP-Co commonly reported in any fields? If it was, the authors should add a discussion.
3) The authors used abbreviations many times, please give the full name when you first use them, such as “FT”, ”CV” and others.
4) It seems the intermediate text in Fig. 5F is wrong. The authors should correct them.
5) The authors used many in situ technology to characterize the catalysts in the reaction. the in-situ instruments information is suggested to be provided.
6) Some descriptions in the introduction are suggested for optimization.
7) Some recent reports for OER catalysts are missing in the references.

Version 1:

Reviewer comments:

Reviewer #1

(Remarks to the Author)
I carefully reviewed the revised version of the manuscript and the authors' response to my concerns. The authors have addressed all my concerns and implemented suggested changes. The manuscript can be accepted for publication in its current format.

Reviewer #2

(Remarks to the Author)
The revised manuscript can be accepted.

Reviewer #3

(Remarks to the Author)
The revised version of this manuscript has shown clear improvement, and the authors have satisfactorily addressed all the comments and suggestions. This manuscript is now at the level suitable for publication in NC.

Reviewer #4

(Remarks to the Author)
The authors have addressed all of my questions and comments, so I recommend publishing the manuscript as it is.

Open Access This Peer Review File is licensed under a Creative Commons Attribution 4.0 International License, which permits use, sharing, adaptation, distribution and reproduction in any medium or format, as long as you give appropriate credit to the original author(s) and the source, provide a link to the Creative Commons license, and indicate if changes were made.
In cases where reviewers are anonymous, credit should be given to 'Anonymous Referee' and the source.
The images or other third party material in this Peer Review File are included in the article's Creative Commons license, unless indicated otherwise in a credit line to the material. If material is not included in the article's Creative Commons license and your intended use is not permitted by statutory regulation or exceeds the permitted use, you will need to obtain permission directly from the copyright holder.
To view a copy of this license, visit https://creativecommons.org/licenses/by/4.0/

Reviewers' comments:

==

Reviewer #1 (Remarks to the Author):

The present manuscript demonstrates the synthesis of CoNPs embedded in a Covalent Organic Polymer (COP) framework, which is used in the polymerization of COPs on ZIF-67, followed by the hydrothermal degradation of ZIF-67. The obtained materials were characterized using various techniques, including XAS, which demonstrated the presence of Co-Co coordination and Co-N/C coordination. Based on linear combination fitting, EXAFS, and pre-edge feature area ratio analysis, the authors proposed the presence of Co-O nanoparticles and a relatively small amount of Co single-atom sites in the catalyst. When tested for the OER and ORR, the catalysts exhibited reduced overpotential and Tafel slopes, comparable to standard Pt and RuO_2 catalysts. DFT analysis revealed that OPM was the dominant pathway for the reaction, while S-COP-Co followed the AEM pathway. The catalyst also demonstrated improved activity in ZABs. Despite the appealing findings presented in the manuscript, the exact chemical and electronic properties of the catalysts are not well understood. Additionally, many control experiments are required to probe the role of Co-O sites in the observed activity. Several critical questions need to be addressed before the manuscript can be considered for publication. The key comments are as follows:

==

Thanks for your encouragement and positive comments.

==

1. While the authors have designed an interesting core-centered H-COP-Co assembly, the motivation for why CoNPs deposited on any surface or a non-core-shell assembly would not exhibit similar catalytic activity is unclear. A control sample without a core-shell structure on COP and other surfaces should be prepared, and its catalytic activity should be compared.

==

We appreciate this comment.

We used the typical carbon substrate (carbon black (CB), Vulcan XC-72R) with ZIF-67 to replace the COP@MOF treated in the same condition, and prepared the contrast sample (named ZIF-67+CB).

The physical mixture of COP and ZIF-67 was also replaced with the COP@MOF and treated under the same conditions to prepare the contrast sample (named ZIF-67+COP).

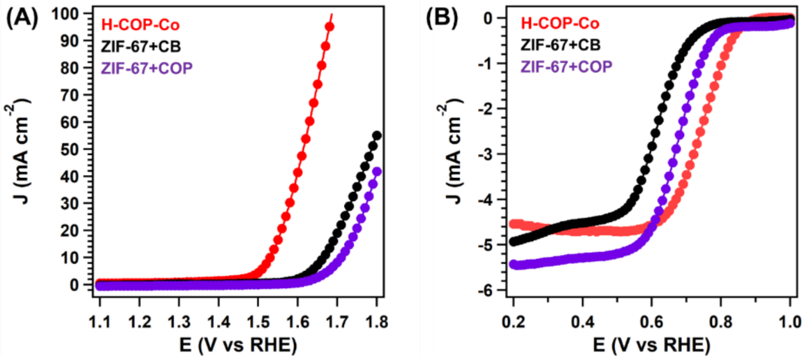

Fig. R1. (A) OER and (B) ORR LSV curves of H-COP-Co (red curve), ZIF-67+CB (black curve), and ZIF-67+COP (purple curve).

As the LSV curves shown, the half-wave potentials for ZIF-67+CB and ZIF-67+COP were 0.61 and 0.68 V, which were 140 and 70 mV negative than that from H-COP-Co (0.75 V). In addition, the H-COP-Co displayed a low overpotential of 300 mV at a current density of 10 mA cm^{-2} for the OER process, which was 135 mV and 180 mV lower than that from ZIF-67+CB and ZIF-67+COP, respectively. Thus, the obtained core-shell catalyst from the template-source construction strategy achieved higher catalytic performance for ORR and OER than other substrate-based catalysts.

==

2. The evidence for the presence of COPs in COP@ZIF is not very clear. Additionally, it is unclear whether COPs completely covered ZIF-67. To address this, TGA data for each component should be provided, along with derivative TGA analysis to highlight the temperatures at which major mass loss occurs. Further, FTIR or Raman spectra of ZIF, COP, and ZIF@COP should be included to confirm the presence of ZIF inside the COP structure.

==

We appreciate this comment.
We have added the TGA and DSC measurements and analysis to describe the interaction between MOF and COP in the synthesized COP@MOF. The retained weight at 1000 °C result showed the value of COP@MOF between COP and ZIF, close to that of ZIF-67, since ZIF-67 occupied the main component.

It is noticed that the DSC curves displayed the obvious peaks that reflect the absorption of heat and the phase evolution of the materials. The ZIF-67 and COP@MOF displayed a similar peak outline since ZIF-67 occupied the main component. The downward peak A was shifted from 560 °C to 576 °C, while the peak B was increased since the interaction between the ZIF-67 and COP. The peaks were sensitive to the materials' phase, which can be reasonably attributed to the COP coating of the ZIF.
We have added the sentences and Supplementary Fig. 3 and 4.

"In addition, the Fourier transform infrared spectroscopy (FTIR) measurements also demonstrated the COP coating on the ZIF-67 (Supplementary Fig. 3). Thermogravimetric analysis (TGA) displayed the retain weight at 1000 °C result showed the value of COP@MOF between COP and ZIF, close to that of ZIF-67 since ZIF-67 occupied the main component (Supplementary Fig. 4A). And the differential scanning calorimetry (DSC) curves also demonstrated the COP coating on the ZIF-67 in COP@MOF (Supplementary Fig. 4B)."

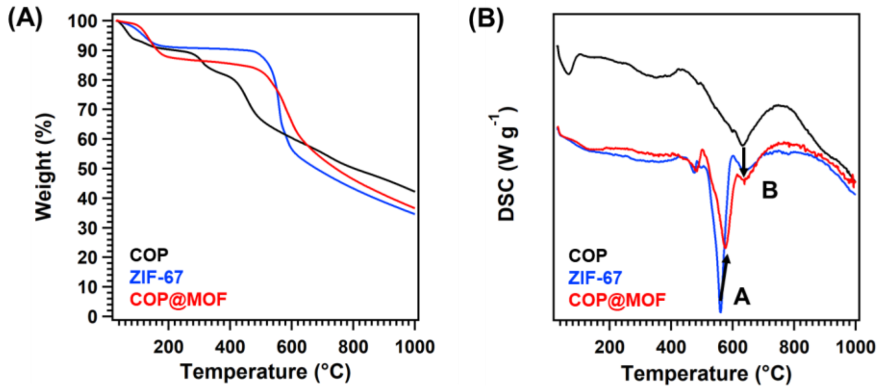

Supplementary Fig. 4. (A) TGA profiles for COP (black curve), ZIF-67 (blue curve), and

COP@MOF (red curve) from room temperature to 1000 °C under N_2. (B) Corresponding DSC curve to (A), the COP curve is up-shifted to compare easily.

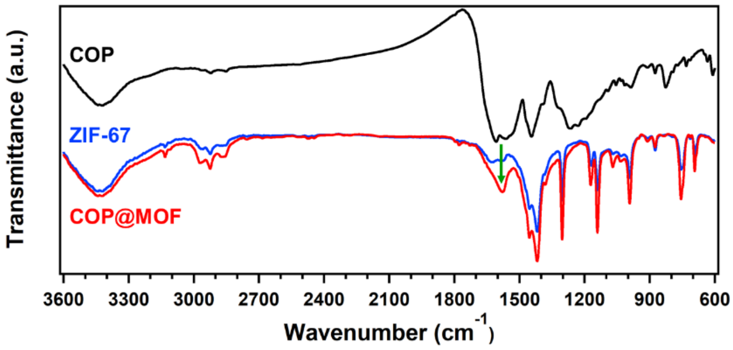

Supplementary Fig. 3. FTIR curves of the COP (black curve), ZIF-67 (blue curve), and COP@MOF (red curve).

==

3. The high energy of the electron beam in TEM can alter the sample morphology. Therefore, SEM images and elemental mapping should be provided. What is the size of the Co-O nanoparticles? High-magnification TEM images should be included with d-spacing measurements. In Figure 1c, TEM images of materials should be presented instead of HAADF.

==

We appreciate these comments.

We have tested the SEM and elemental mapping images and added them to the manuscript. The SEM images of H-COP-Co were shown as the cubic outline and wrinkled surfaces, which implied the COPs had inherited the shape of the ZIF. And the EDS images showed Co elements mainly dispersed at the shell of the COP cubes.

We have added the sentences and Supplementary Fig. 5-7.

"We also find the lattice fringe belongs to Co oxides in the high resolution TEM images (Fig. 1D, Supplementary Fig. 5). Moreover, the scanning electron microscopy (SEM) images also displayed the H-COP-Co, also supporting the material evolution from COP@MOF (Supplementary Fig. 6 and Fig. 7)."

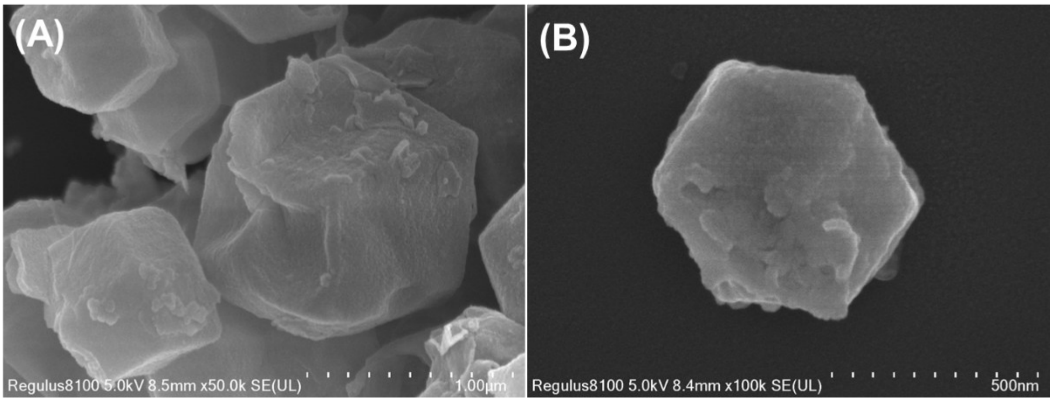

Supplementary Fig. 6. SEM images of the H-COP-Co.

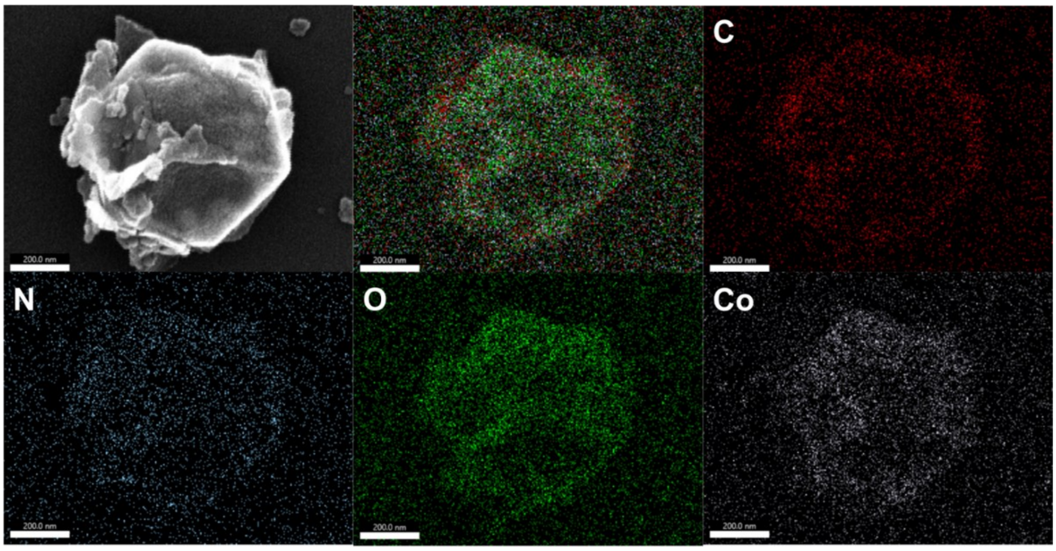

Supplementary Fig. 7. SEM energy dispersive X-ray spectroscopy (EDS) mapping of H-COP-Co.

We also collect the statistics of the CoO_x particle size, the average size is 32.2 nm (Supplementary Fig. 5. (B)). In addition, we have added the HR-TEM images and measured the lattice distances. The lattice fringe was measured as 0.244 nm, which can be attributed to the (111) facets from the CoO phase. (*Nat. Commun.* 2024, 15, 1012)

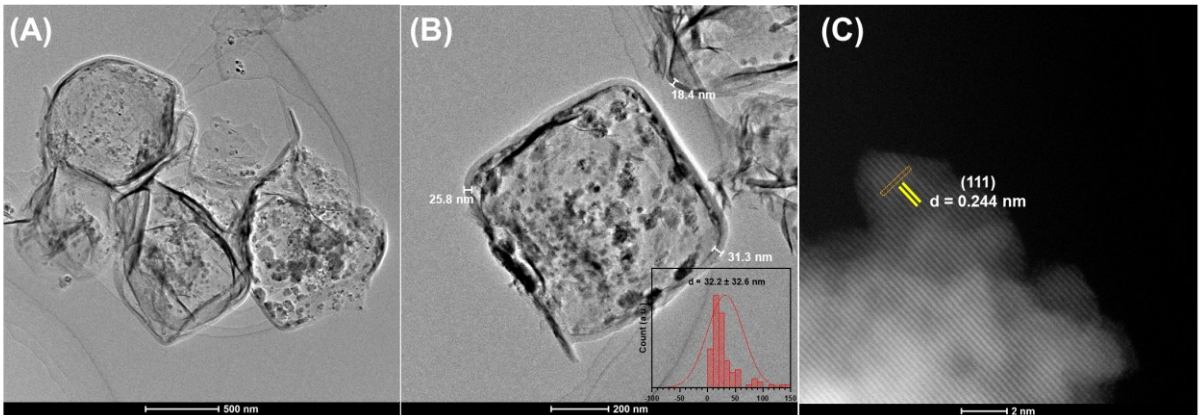

Supplementary Fig. 5. (A) and (B) The TEM images of H-COP-Co. Insert pattern in (B): the statistics of the cobalt oxide size. (C) The high resolution TEM image of the H-COP-Co.

As you suggested, we also adjusted Fig. 1 as follows:

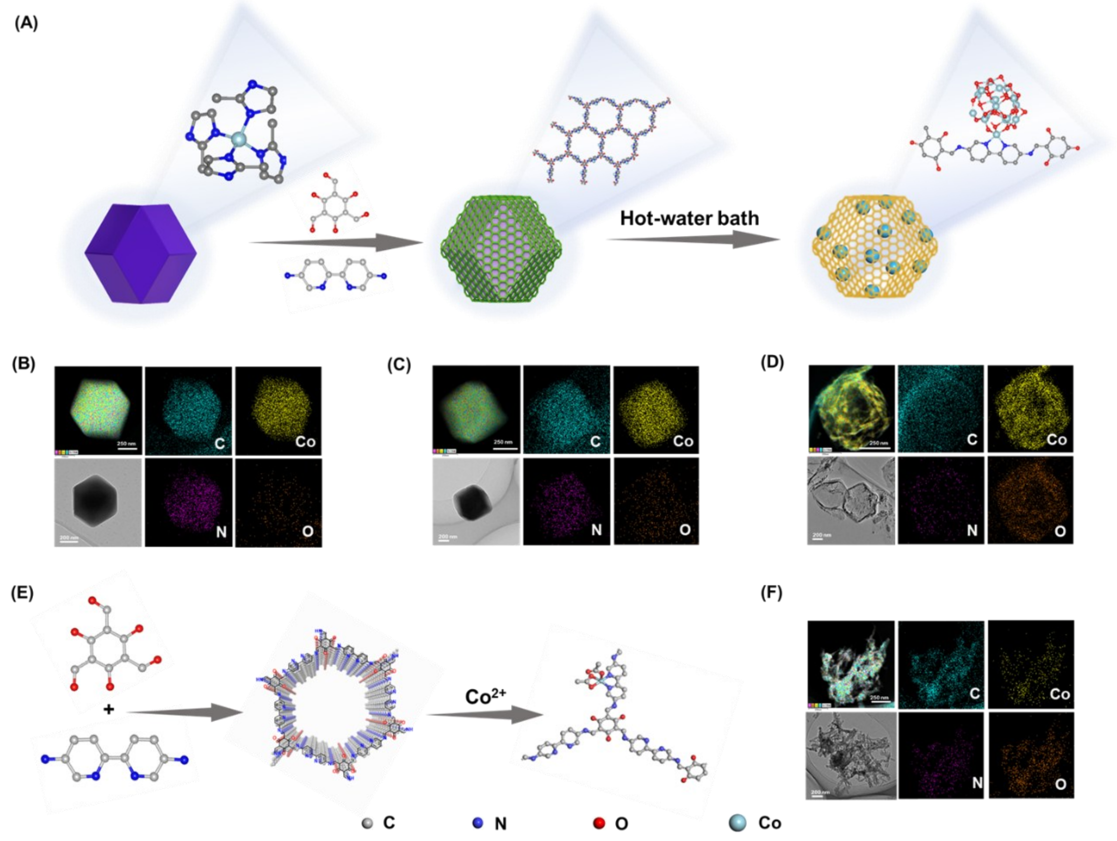

==

4. HAADF-STEM images of the materials at high magnification should be included to clearly show the nanoparticles and single atoms.

==

We appreciate this comment. We have updated the HAADF-STEM images (Fig. 4K and 4L) exhibition to comprehensively exhibit the single atom sites and nanoparticles.

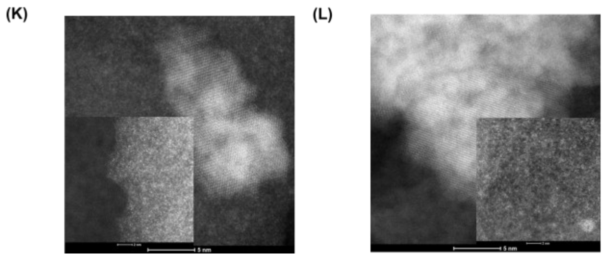

Fig 4. High-angle annular dark-field scanning transmission electron microscopy (HAADF-STEM) images of H-COP-Co (K) before and (L) after reaction, showing the single atom sites (insert image) and nanoparticle areas.

==

5. It remains unclear whether the observed catalytic activity is due to CoNPs or the high concentration of Co atoms in the catalyst. To distinguish their roles, the authors should remove the nanoparticles via dilute acid washing and test the remaining CoSA-COP-Co for OER and ORR activity.

==

We appreciate this comment.

We have added the comparison of LSV curves with H_2SO_4 washed. It can be evidently observed that the performance decreases after the treatments, which was caused by the destruction of the active sites in the materials.

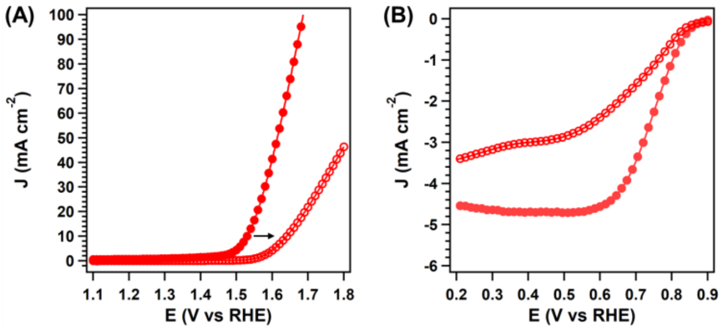

Fig. R2. (A) OER and (B) ORR LSV curves of H-COP-Co before (solid-dot curve) and after (circle curve) H_2SO_4 solution treatment.

==

6. The authors claim that Co is present as CoO nanoparticles based on XANES and EXAFS data. However, the EXAFS spectra of H-COP-Co appear to resemble those of Co nanoparticles rather than Co-O. Additionally, the comparison between Co NP and Co SACs was calculated using these peaks, which may be a misinterpretation. These discrepancies should be clarified.

==

We appreciate this comment.

The second shell of the Co atoms' identification may be confused. Herein, we extract the Co foil, CoO, and H-COP-Co in Fig. R3 to exhibit and explain the species' identification. In fig. R3 (A), it looks like the H-COP-Co peak at 2.4 Å is close to that of the main peak (2.16 Å) of the Co foil. Actually, the Fourier transformed EXAFS curves reflect the atomic distance in real space, Co foil means the bulk Co metals, with the typical Co-Co atomic distance corresponding to the peak at 2.16 Å. Usually, the prepared catalysts were the nanometer size of metallic Co particles, since the nanometer size effect the bond length usually shorter than that of bulk materials (*J. Phys. Chem. B* 1997, 101, 14, 2691–2701; *Catal. Sci. Technol.*, 2024, 14, 2580-2592). Similarly, the nanometer size effect of metal oxides also commonly exists, so the reduced atomic distance of Co-(O)-Co should be displayed in the nanometer size CoO_x. Therefore, the second shell identification should be the Co-O-Co path rather than the Co-Co path.

Moreover, considering the E space is the initial data of the sample without the mathematical treatment, which includes the most characteristic of the samples. We compared the E space curve of the outline and the absorption vibration of the H-COP-Co with the Co foil and CoO, which exhibited the similarity between H-COP-Co and CoO rather than the Co foil (Fig. R3B). We excute the linear combination fitting of H-COP-Co by using the Co foil and CoO curves, the fitting result tends to the 100% content of the CoO and 0% content of the Co foil (Fig. R3C).

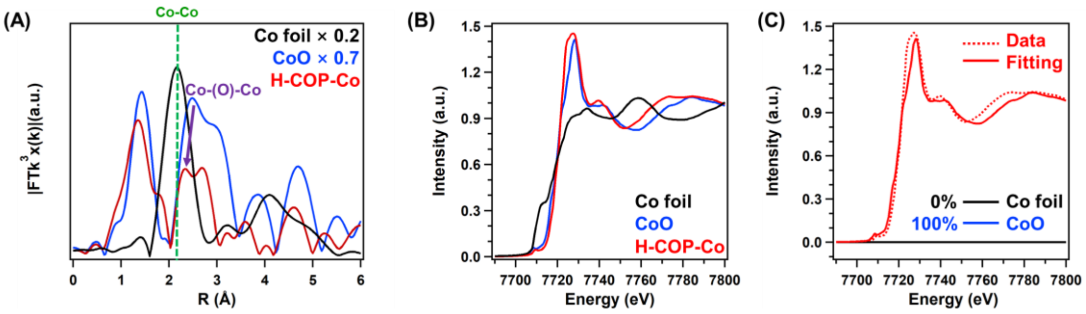

Fig. R3. (A) The k^3-weighted FT-EXAFS and (B) XANES spectra of Co foil (black curve), CoO (blue curve), and H-COP-Co (red curve), respectively. (C) LCF analysis of the H-COP-Co.

==

7. To confirm that Co-O sites, rather than metallic Co sites, are responsible for the main oxidation activity, the H-COP-Co catalyst should be treated with weak H_2O_2 to oxidize metallic Co to Co-O, and its catalytic activity should be reassessed.

==

We appreciate this comment.

We have tried to use the H_2O_2 to treat H-COP-Co, but the sample dissolved. We deduced it may be attributed to the low content of substrate, so we used the double content of COP to construct the COP@MOF and synthesized 2-H-COP-Co. The stability of 2-H-COP-Co is enhanced and better than that of H-COP-Co under the H_2O_2 solution. We have compared the electrocatalysis performance of 2-H-COP-Co before and after H_2O_2 treatment. According to the results, the OER performance is evidently improved after reacting with H_2O_2 solution, while the ORR performance is maintained and with a slight improvement. It may be attributed to the oxidation potential at the OER range promotes a high valence state of Co, reserving and reaction. Under the reduced potential for ORR, the oxidized Co elements tend to reduce to their original states.

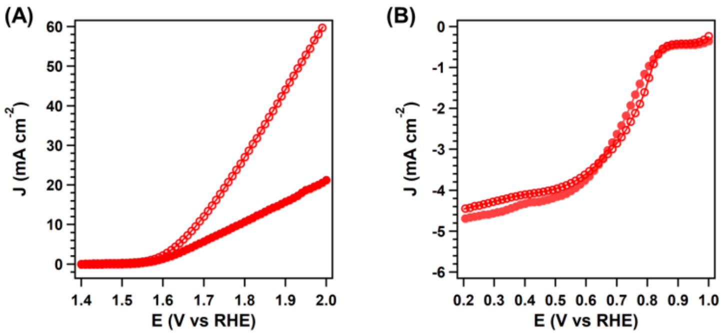

Fig. R4. (A) OER and (B) ORR LSV curves of 2-H-COP-Co before (solid-dot curve) and after (circle curve) H_2O_2 solution treatment.

==

8. The degradation of ZIF-67 under hydrothermal conditions should be confirmed using other support materials. A reference should be provided to support this claim.

==

We appreciate this comment. We also used the carbon black as support for the hydrolyzed ZIF-67, and the PXRD result revealed retained ZIF peaks and new peaks from the product were assigned to $Co(OH)_2$ phase.

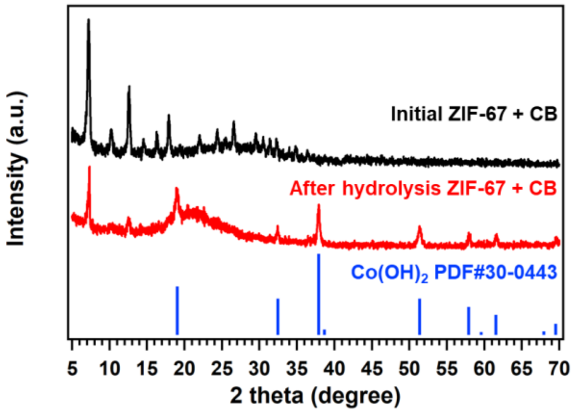

Fig. R5. PXRD patterns of the initial and treated ZIF-67+CB.

==

9. The structural model presented suggests that only one Co atom is coordinated to two nitrogen atoms, while another Co is present as Co-O. In this case, the average coordination number for Co-N should be much lower than for Co-O. However, the authors report a Co-N: Co-O ratio of approximately 1:2. Similarly, the Co-Co coordination number of ~4 does not support the presence of Co in nanoparticle form. These inconsistencies should be addressed.

==

We appreciate this comment. We agreed with your viewpoint that the Co-N coordination number is greatly lower than the Co-O coordination number in the H-COP-Co materials.

However, the EXAFS technology to distinguish the metal-coordinated N or O atoms is commonly believed to be difficult under most conditions, since the similar contribution of their backscattering parameters (*Coord. Chem. Rev.* 2020, 423, 213466). The analysis of the XAFS data in this manuscript is with respect to this principle, and this is necessary. The Co-N component with a similar bond length and a small proportion compared to Co-O in the material. Therefore, the Co-N/O_1 and Co-N/O_2 mostly describe the two different bond lengths of Co-O that also include the Co-N of the coordination to the frameworks of COPs.

Regarding the Co-Co coordination number is the value of ca.4 and not as large as the standard cobalt oxides, which is caused by the nanometer size effect of the cobalt oxides. Different from ideal crystals and bulk materials, the nano-sized metal or metal oxides have many edges and defects that cannot be ignored. The nano size effects on the coordination number are already demonstrated (*Phys. Chem. Chem. Phys.*, 2010, 12, 5562–5574).

We have revised sentences and added citations to the manuscript:

“Since the similar atomic distances of N and O, we have merged the Co-N and Co-O as the Co-N/O paths [54].

54. Zimmermann, P. et al. Modern X-ray spectroscopy: XAS and XES in the laboratory. *Coord. Chem. Rev.* **423**, 213466 (2020).”

“Combined with other characterization results, the two Co-N/O paths contain the Co-N, and Co-O from the single Co sites, and most of them are contributed by the CoO_x clusters[55]. The low average Co-Co C.N. is attributed to the nanometer size effects, as C.N. is not as large as normal bulk Co oxides, and the single Co sites in H-COP-Co also decrease the average C.N. of Co-Co.

55. Beale, A. M. & Weckhuysen, B. M. EXAFS as a tool to interrogate the size and shape of mono and bimetallic catalyst nanoparticles. *Phys. Chem. Chem. Phys.* **12**, 5562–5574 (2010).”

==

10. Why does the XRD pattern of H-COP-Co not exhibit any peaks related to CoO? Is this due to the thickness of the COP shell or the small nanoparticle size? The shell thickness should be reported.

==

Thank you for the reviewer's keen insight.

We agreed with your points. The Co oxides were dispersed on the thick amorphous phase COPs, which may decrease their diffraction signals.

We have added the COP shell thickness measurements in the microscopic image. And we also added the sentences to the manuscript:

"Subsequent hydrolysis transformed COP@MOF into H-COP-Co, which displayed several hollow cubic structures that could be identified as COP shells with 10~30 nm thickness (Fig. 1D, Supplementary Fig. 5)."

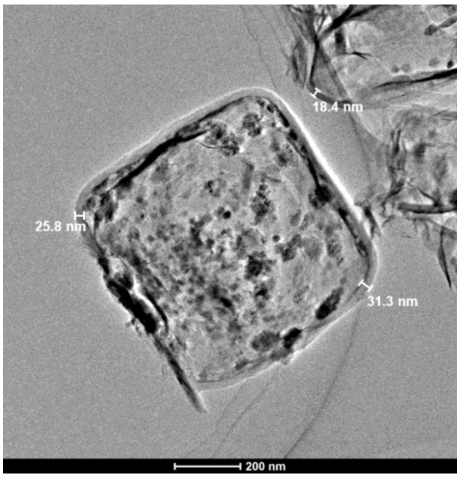

Supplementary Fig. 5. (B) TEM image of the H-COP-Co.

==

11. What was the oxidation state of Co in ZIF-67? The origin of the satellite peak in the XPS spectra of ZIF-67 should be clarified.

==

We appreciate this comment. We have executed the XPS peaks deconvolutions again.

The binding energy of the Co elements is special; the Co (+3) peaks' location is lower than Co (+2) peaks (*J. Electron Spectrosc.* 2009, 173, 88–95; *J Mater Sci-Mater El.* 2015, 26, 9941–9948). Therefore, we referred to the previous literature to deconvolute the peaks, the Co oxidation states are between +2 and +3. This result also agrees with the previous XPS report about ZIF-67 (*Small* 2018, 14, 1803576).

The origin of satellite peaks is usually considered as the complicated materials' surface chemistry environments that contain the multiple-splitting effect, spin coupling of the ionic states and electrons, the ligand electrons transfer, and other effects. Many reports commonly believe it is unable to analyze clearly. (*J. Electron Spectrosc.* 1975, 7, 197-213; *Chem. Phys. Lett.* 1974, 26, 163–166).

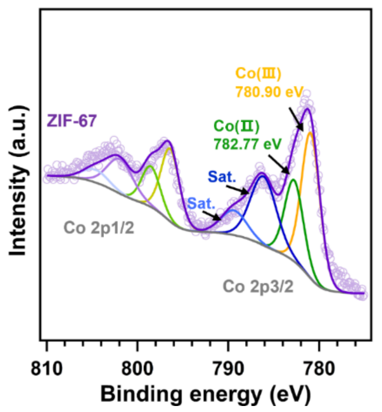

Supplementary Fig. 9. XPS spectra of Co 2p for H-COP-Co.

We have revised Fig. 2B and the sentences as follows:

"Furthermore, the high-resolution Co spectra of COP@MOF, H-COP-Co, and S-COP-Co displayed a similar outline, which could be deconvoluted into peaks corresponding to the composition of +2 and +3 oxidation states' peaks and other satellite features[46,47] (Fig. 2B and Supplementary Fig. 9).

46. Zhu, R. et al. π-Conjugated molecule boosts metal–organic frameworks as efficient oxygen evolution reaction catalysts. *Small* 14, 1803576 (2018).

47. Jiang, L. et al. In situ pyrolysis of ZIF-67 to construct $Co_2N_{0.67}$@ZIF-67 for photocatalytic CO_2 cycloaddition reaction. *Inorg. Chem.* 63, 14761–14769 (2024)."

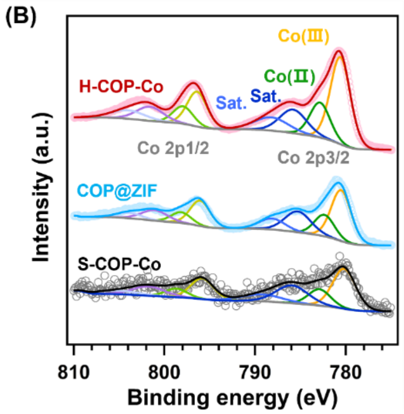

Fig. 2 (B) Co 2p spectra of S-COP-Co, COP@ZIF, and H-COP-Co.

==

12. The white line intensity of S-COP-Co is higher than that of H-COP-Co, yet the oxidation state determination does not reflect this trend. The methodology used to calculate the oxidation state of Co in each material based on the XANES white line intensity should be clearly explained. Did the authors use the center point of the linear region of the highest peak intensity for the calculation?

==

We appreciate this comment.

The K-edge Co XAFS measurement corresponds to the core 1s→4p unoccupied orbital transition of the electrons. The K-edge white line peak intensity depends on multiple factors like coordination environments, electronic states, and so on. The oxidation states evaluation is usually through the

energy of the absorption edge of the samples (*Nat. Catal.* 2019, 2, 134–141; *Nat. Chem.* 2020, 12, 1060–1066). In this manuscript, we collect the half peak K-edge energy of samples and standard samples to estimate the oxidation states.

Regarding the analysis of the XANES, we used two methods to offer two views of oxidation states and phase, which try to more comprehensively reveal the states of Co sites. On the one hand, as above, we have explained that the absorption energy location reflects the oxidation states. On the other hand, linear combination fitting (LCF) analysis is based on the XANES, which extracts the structural features of the sample, reflecting the phase and structural information.

==

13. Figures 2d and 2i-j appear significantly different in terms of fitting. The fitting parameters used should be described in the supporting information. Additionally, details about the XANES and EXAFS measurement conditions, beamline parameters, and the in-situ measurement cell setup should be included. A picture of the inset FTIR cell should also be provided.

==

We appreciate this comment. We have revised the supporting information and added the Supplementary Fig. 19 and 24:

3. XAFS measurements and data processing

XAFS measurements. The main XAFS measurements were performed at the BL20U1 station in Shanghai Synchrotron Radiation Facility (SSRF)[1]. The electron storage ring of SSRF was operated at 3.5 GeV, with a maximum current of 200 mA. Metals K-edge XAFS data were collected using a fixed-exit Si (111) double-crystal monochromator. A Lytle detector was used to collect the fluorescence signal, and the energy was calibrated using a metal foil. In situ Co K-edge XAFS measurements were conducted with catalyst-coated carbon paper as the working electrode, Ag/AgCl as the reference electrode, and a carbon rod as the counter electrode in an electrochemistry cell (Supplementary Fig. 19).

XAFS analysis. Utilizing the ATHENA module of the IFEFFIT software packages, the obtained EXAFS data were analyzed according to the standard procedures[2]. To determine the oxidation states of the Co atoms in the samples, reference Co oxides were used to extract standard absorption edge energies. A calibration curve was constructed by linear fitting of the normalized edge jumps of the reference compounds at an absorbance of 0.5. The EXAFS contributions were separated from different coordination shells by using a Hanning window (dk=1.0 $Å^{-1}$). Subsequently, the quantitative curve-fittings were carried out in the R-space with a Fourier transform k-space range using the module ARTEMIS of IFEFFIT. During the curve-fitting, the overall amplitude reduction factor S_0^2 was fixed to the best-fit value determined from fitting the data of the metal foil. For the sample, the structural parameters, such as the coordination number N, interatomic distance R, the Debye-Waller factor σ^2, and the edge-energy shift ΔE_0, were allowed to vary during the fitting process.

1. Hijazi, I. et al. Carbon nanotube-templated synthesis of covalent porphyrin network for oxygen reduction reaction. *J. Am. Chem. Soc.* **136**, 6348–6354 (2014).
2. Beuerle, F. & Gole, B. Covalent organic frameworks and cage compounds: design and applications of polymeric and discrete organic scaffolds. *Angew. Chem., Int. Ed.* **57**, 4850–4878 (2018).

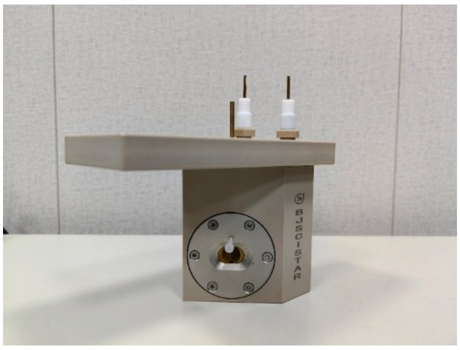

Supplementary Fig. 19. The picture of the in situ XAFS electrochemistry cell.

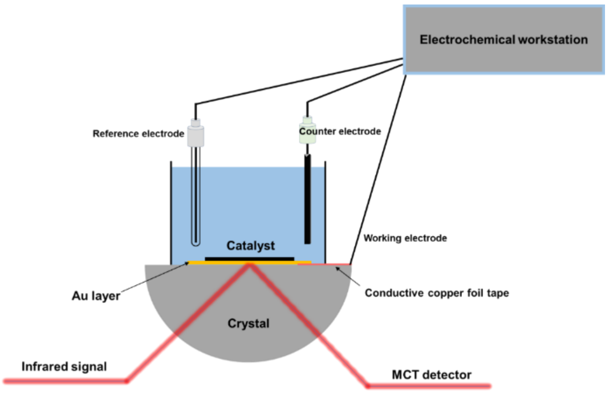

Supplementary Fig. 24. The schematic graph of the in situ electrochemical ATR-FTIR.

==

14. EIS spectra of the catalyst should be included. The onset potential and fitting parameters should be provided to assess charge transport and charge transfer resistance.

==

We appreciate this comment. We have added the electrochemical impedance spectroscopy (EIS) measurements at the OER onset potential of three samples and estimated their charge transfer resistance. It is noticed that the H-COP-Co displayed the smallest charge transfer resistance of these samples, which implies its fastest charge transfer.

We also added the sentences and Supplementary Fig. 14:

“As shown in electrochemical impedance spectroscopy (EIS) Nyquist plotting, the H-COP-Co displayed the smallest charge transfer resistance of these samples, which implied its fastest charge transfer (Supplementary Fig. 14).”

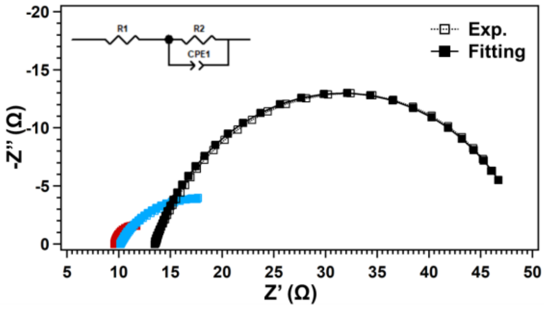

Supplementary Fig. 14. Electrochemical impedance spectroscopy of the H-COP-Co, COP@MOF,

and S-COP-Co catalysts (Inset: equivalent fitted circuit diagram).

==

15. The change in Co-Co peak intensity upon applying a high potential suggests nanoparticle formation. This could be due to either the agglomeration of atoms, as the authors suggest, or the degradation of COPs at this potential. The authors should confirm whether the support structure remains chemically and physically intact after the reaction.

==

Thank you for the reviewer's keen insight.

We have measured a series of characterizations of the H-COP-Co after reaction. The morphology of COPs was changed, and fractures of the hollow cubes were observed. We deduced it was attributed to the local structural evolution of Co sites. Moreover, we can see that the oxides were aggregated and still loaded on the COPs. The XPS described the chemical information tests displayed similar curves compared to the initial samples.

We also updated related sentences as follows:

"The PXRD and XPS of after-reacted H-COP-Co demonstrated that the phase and chemical properties of H-COP-Co are maintained, while the morphology is not kept as the initial may be caused by the local Co sites reconstruction and migration, inducing the evolution of bulk COP substrates (Supplementary Fig. 21-23)."

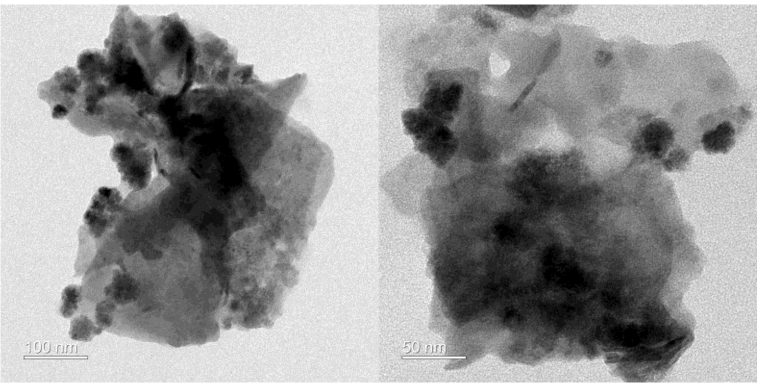

Supplementary Fig. 21. TEM images of the after reaction H-COP-Co.

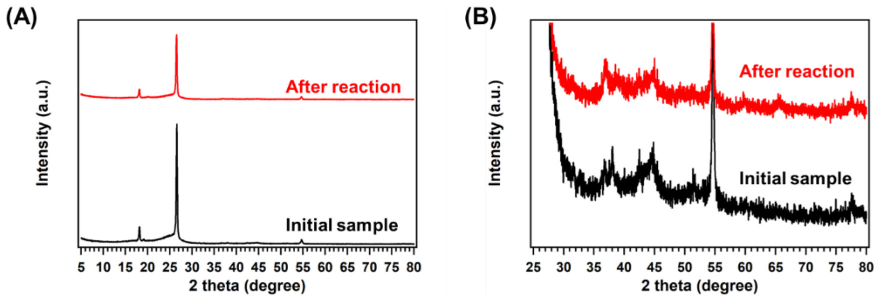

Supplementary Fig. 22. (A) PXRD patterns of the initial (black curve) and after reaction (red curve) sample loaded on the carbon paper. (B) Local enlarged pattern of the (A).

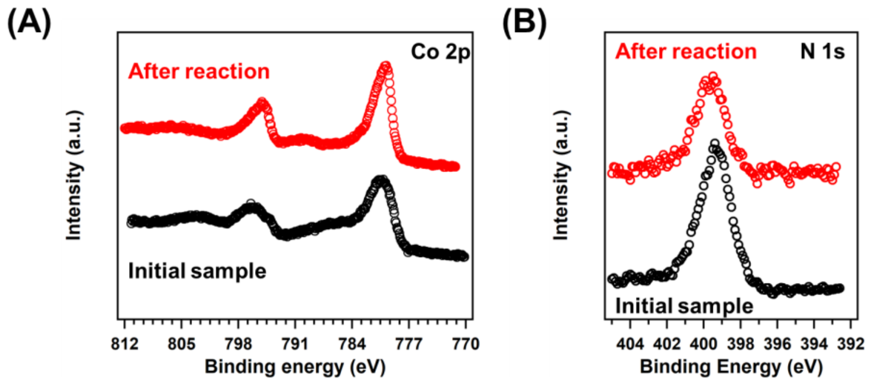

Supplementary Fig. 23. (A) Co 2p XPS spectra of the initial (black curve) and after reaction (red curve) sample. (B) N 1s XPS spectra of the initial (black curve) and after reaction (red curve) sample.

==

16. The chemical structure of COP suggests that it is not conductive. However, the material still delivers a significant current density, as shown in Figure 3a. Furthermore, long-term stability was tested at a very low current density. In industry-standard testing, stability is typically demonstrated at a current density of 10 mA cm^{-2}.

==

Thank you for the reviewer's keen insight.

We agreed that good electron conductivities can promote electrocatalysis, since the designed H-COP-Co contained many cobalt oxide phases that promoted the electron conductivities. As shown in Fig. 3A, the S-COP-Co only comprises the COP and single Co sites that deliver a low current density with a large overpotential.

Initially, we tested the current density around 10 mA cm^{-2}, but we used the I/I_0 to normalize the current density so it looks close to 1. To avoid this misunderstanding for readers, we have replaced this figure using the original current density values.

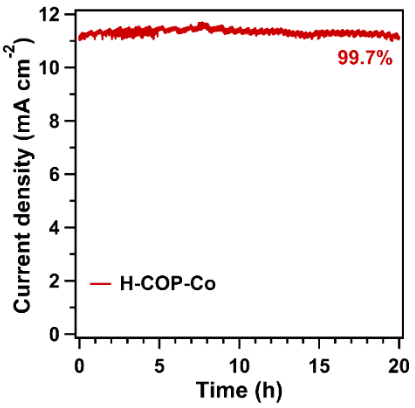

Fig. 3 (D). Durability test of chronoamperometry curves for H-COP-Co.

==

17. Bader charge analysis, electron density distribution plots, and density of state calculations should be added to the DFT analysis.

==

We appreciate this comment.

We have added the corresponding calculations and added the sentences: "The Bader charge and charge difference density result showed the CoO_x model comprises many accessible Co atoms for

the OER (Supplementary Fig. 25-27). The projected density of states (PDOS) displayed the different d band of the single Co site model and the CoO_x model. The higher d band center for single Co sites indicated the stronger adsorption to intermediates, which indicated the high energy barrier for intermediates oxidation and desorption (Supplementary Fig. 28)."

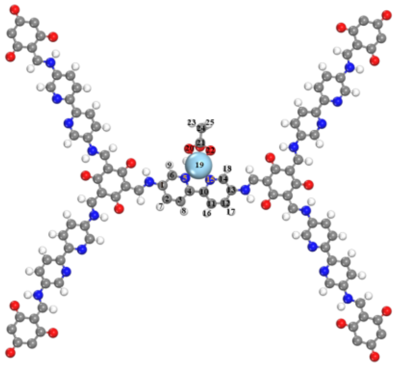

Atomic number	Bader |e|	Atomic number	Bader |e|
1	0.396	14	0.493
2	-0.103	15	-1.131
3	-0.006	16	0.040
4	0.424	17	0.072
5	-1.141	18	0.151
6	0.488	19	1.093
7	0.144	20	-1.073
8	0.105	21	1.484
9	0.139	22	-1.077
10	0.381	23	0.063
11	0.065	24	-0.062
12	0.059	25	0.049
13	0.367		

Supplementary Fig. 25. Charge distribution by Bader charge analysis of the catalytic models for S-COP-Co.

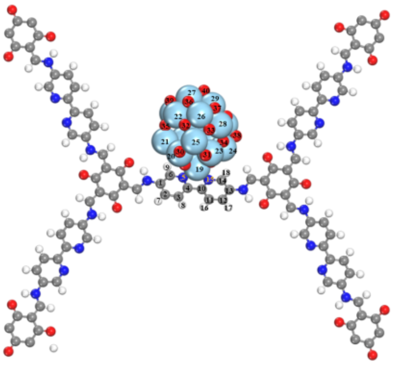

Atomic number	Bader |e|	Atomic number	Bader |e|	Atomic number	Bader |e|
1	0.354	17	0.100	33	-0.846
2	0.045	18	0.177	34	-0.820
3	-0.067	19	1.229	35	-0.874
4	0.428	20	0.992	36	-0.795
5	-1.149	21	0.802	37	-0.797
6	0.504	22	0.986	38	-0.657
7	0.124	23	1.208	39	-0.787
8	0.090	24	1.064	40	-0.653
9	0.209	25	1.299		
10	0.454	26	1.054		
11	0.021	27	1.047		
12	-0.052	28	1.076		
13	0.435	29	1.058		
14	0.508	30	-0.728		
15	-1.121	31	-0.711		
16	0.075	32	-0.878		

Supplementary Fig. 26. Charge distribution by Bader charge analysis of the catalytic models for H-COP-Co.

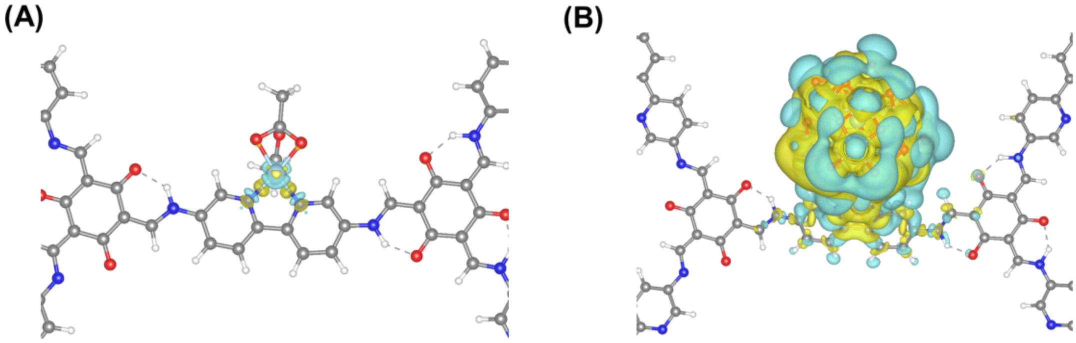

Supplementary Fig. 27. The charge difference density of the catalytic models for (A) S-COP-Co and (B) H-COP-Co, respectively. Pink and green isosurfaces (0.013 $|e|/Å^3$) denote regions of electron accumulation and depletion, respectively. Yellow and blue isosurfaces (0.013 $|e|/Å^3$) denote regions of electron accumulation and depletion, respectively.

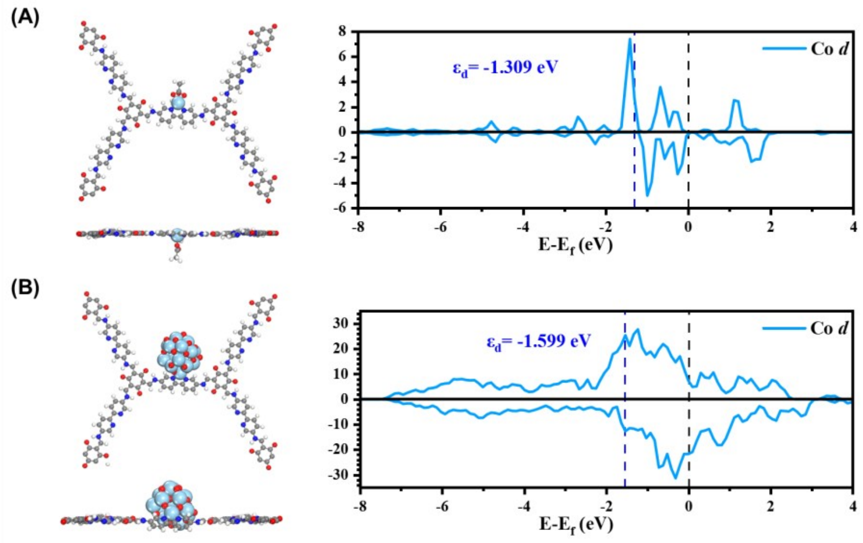

Supplementary Fig. 28. Projected density of states (PDOS) of d orbitals for Co in the catalytic models for (A) S-COP-Co and (B) H-COP-Co, respectively. The Fermi level (E_f) is aligned at 0.00 eV. The d-band center positions are marked by the vertical dashed line labeled by ε_d.

==

18. The C, N, O, and Co content reported by XPS quantification should be specified as either weight percent (wt%) or atomic percent (at%). Both values should be provided in the supplementary information. Additionally, the elemental composition of materials after the reaction should be reported. The metal content after the reaction should also be analyzed using ICP.

==

We appreciate this comment. We have added the content estimation of the after reaction H-COP-Co by the ICP and XPS.

The catalyst is scraped from the carbon paper to test the ICP, which may contain the scrap chipping of carbon papers may cause the result to be on the low side. In summary, the cobalt content is decreased after the reaction, which may relate to the dynamic behavior of Co atoms and solvent-ligand competition under reaction conditions.

We also added the sentences to the manuscript:

"The content of the Co atoms in H-COP-Co is decreased (Supplementary Table 2 and 7)."

Supplementary Table 2. The report of the ICP test.

Sample	Elements	Weight (wt%)
COP@ZIF	Co	25.43
H-COP-Co	Co	56.86
S-COP-Co	Co	2.96
After reaction H-COP-Co	Co	29.94

Supplementary Table 7. XPS content estimation of H-COP-Co loaded on carbon paper before and after reaction.

Elements	Before (wt.%)	After (wt.%)
C	48.70	57.49
N	3.98	0.92
O	24.29	24.61
Co	23.02	16.99

==

19. Several grammatical and formatting errors need to be corrected. The purity of each chemical should be included in the supplementary experimental section. Instrument operating conditions and sample preparation details should be clearly defined. In Supplementary Figure 7D, the Y-axis should be corrected.

==

Thank you for the reviewer's keen insight. We appreciate this comment and have carefully read the manuscript and revised the faults.

We also updated the supporting part as follows:

1. Materials & Characterization

2-Methylimidazole (≥99.5%, Aladdin), 1,3,5-Benzenetricarboxaldehyde,2,4,6-trihydroxy (TP) (>98%, Tensos Biotech), [2,2'- bipyridine]-5,5'-diamine (BPY) (>98%, Tensos Biotech), cobalt nitrate hexahydrate (AR), methanol (MeOH, AR), tetrahydrofuran (THF, AR), cobalt acetate (≥99.5%), ethanol (EtOH, AR), 5 wt.% Nafion, and potassium hydroxide (KOH, ACS) were from Tansoole.

2. Catalyst preparation

Synthesis of ZIF-67. A mixture of $Co(NO_3)_2 \cdot 6H_2O$ (1.0275 g, 3.5 mmol), 2-Methylimidazole (4.59 g, 0.056 mol), and 50 mL methanol was added and stirred. Subject the mixture to a high-pressure reactor at 110 °C for 24 hours. The ZIF-67 solid was washed with MeOH and THF in sequence and then dried in a vacuum.

Synthesis of COP@ZIF. Fetch 100 mg of ZIF-67 and 5.759 mg of BPY (0.03 mmol), and dissolve them in 67 mL of THF. Prepare 4.289 mg of TP (0.02 mmol)and 5 mL of THF mixture to stir with the previous solution for 12 h. The COP@ZIF was obtained after vacuum filtration and drying.

Synthesis of H-COP-Co. 30 mg COP@ZIF dissolving in 40 mL deionized water, react for 30 min in an 80 °C hot water bath, approximately 10 mg of H-COP-Co can be obtained after filtration and drying.

Synthesis of S-COP-Co. The BPY (57.59 mg, 0.3 mmol) and TP (42.89 mg, 0.2 mmol) were stirred in THF solution for 12 h to obtain Tpbpy. Fetch 90 mg of Tpbpy and 60 mg of cobalt acetate, and dissolve them in 60 mL of MeOH for 4 h. The S-COP-Co solid was washed with MeOH and THF in sequence and then dried in a vacuum.

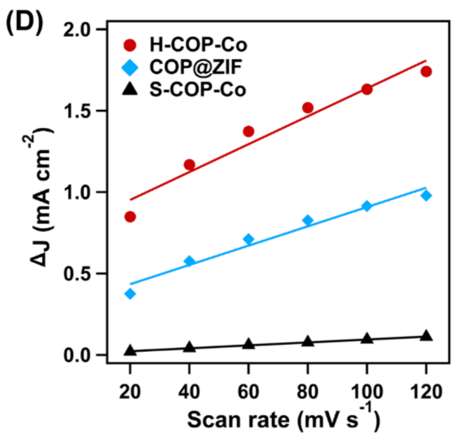

Supplementary Fig. 15. (D) C_{dl} values for H-COP-Co, COP@ZIF, and S-COP-Co.
(Previous Supplementary Fig. 7 is the Supplementary Fig. 15 in this version.)

==

Additional suggestions:

1) Avoid using hyperbolic terminology, such as "exceptional."
2) Define "reversible covalent reactions" clearly.
3) The sentence "In contrast, S-COP-Co and the COP exhibited similar weak diffraction···" is incorrect. Single atoms do not exhibit diffraction peaks, and the absence of diffraction peaks is not direct evidence of the presence of single atoms.
4) The phrase "smooth cubic structures" is not appropriate.
5) Reference formatting should be consistent with journal guidelines—titles should be in either sentence case or title case.

==

We appreciate this comment and have carefully read the manuscript and optimized the expression. We also updated related sentences as follows:

1. We have used another word to replace the "exceptional".
2. A reversible covalent reaction is a dynamic covalent chemical reaction based on thermodynamic equilibrium, which achieves dynamics within molecules through reversible formation and cleavage of covalent bonds. To avoid making readers misunderstand, we have used "dynamic covalent bonds" to replace the "reversible covalent reactions"(*Chem. Soc. Rev.*, 2013,42, 6634-6654).
3. The covalent organic polymers displayed the diffraction peaks at low-angle range, so we optimized the description as "In contrast, S-COP-Co and the COP exhibited similar peaks from crystalline polymers, indicating the single Co sites on the S-COP-Co."
4. We have revised as "typical cubic structures".
5. We have amended reference formatting.

==

We appreciate this reviewer very much for the above valuable comments that greatly improved the quality of the manuscript.

==

==

Reviewer #2 (Remarks to the Author):

The authors reported high-density active sites on hollow covalent organic polymers for oxygen electrocatalysis. Generally, there are much more improvements and clarifications both in the technical execution and in the overall presentation. It does not reach the high standards of Nature Communications, and thus is not suitable to publish in the journal.

==

Thanks for your comment.

==

1. The H-COP-Co catalyst synthesized by the authors exhibits an OER overpotential of 270 mV and an ORR E1/2 of 0.753 V. However, it is noteworthy that numerous Co-based SACs have already demonstrated OER overpotentials lower than 250 mV and ORR E1/2 higher than 0.90 V. In light of these advancements, could the authors elaborate on the practical significance of oxygen electrocatalysis for the H-COP-Co? Specifically, what unique advantages or improvements do this method offer compared to existing high-performance catalysts?

==

Thanks for your comment.

This report focused on the structural strategy of composed COPs, which contributed the enlightenment to extending the COPs to various applications with novel design strategies. The next generation of composite COPs was commonly believed as a promising material for frontiers in many fields. However, the morphology controlling, active species type, and content increase of COPs are still a significant challenge and important for application. In this work, we proposed the template-source construction strategy and applied it to the oxygen electrocatalysis, achieving the key points:

(1) Creatively construct the hollow nano cubes of COPs through the template-source construction strategy, compared to the traditional post-modification strategies to introduce the single metal sites, this strategy revolutionarily increased the active metal content from 2.96 wt.% to 56.86 wt.%.

(2) Compared to the traditional post-modification strategy for catalytic COP, the turnover frequency of template-source constructed COPs for the OER by over 10.4 times, exhibiting a low overpotential of 300 mV.

(3) Comprehensive in situ spectroscopies and theoretical calculations revealed the template-source constructed COPs reconstruction of the active sites in oxygen electrocatalysis and demonstrated an effective oxide path mechanism rather than the traditional adsorbate evolution mechanism on traditional single-atom sites.

The main significance of this work is not about the performances. Moreover, we have compared it with many advanced COP-based and other advanced materials, and found that the H-COP-Co was excellent among them.

==

2. They declared that H-COP-Co demonstrated an increase in active metal content, from 2.96% to 56.86%, however, from the HAADF-STEM images, the density of bright dots is very sparse, even less than those with 1% metal content reported previously (e.g. Nat. Catal. 2020, 3, 1044–1054). Why?

==

We cannot agree with this point.
We have extracted the pictures from your listed report. And their metal sites density looks not much than our exhibited areas. More importantly, the listed report used ZIF-8 as support for the Co sites, as we all know, the single dispersed Zn was also retained much in the carbon supports, and they would also be exhibited in the HADDF-STEM images.

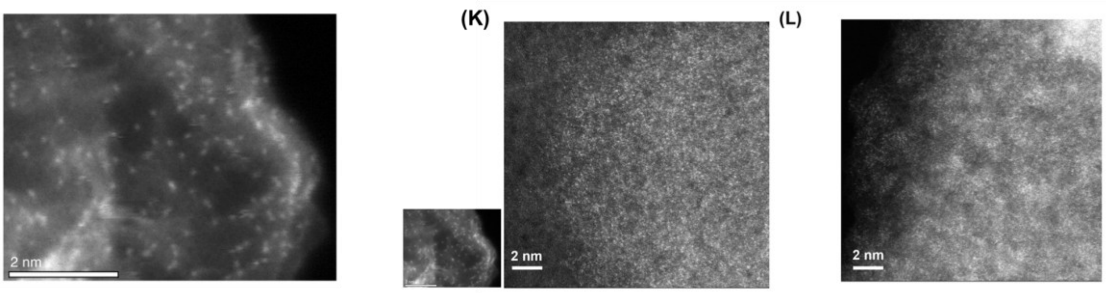

Nat. Catal. 2020, 3, 1044–1054

Initial version in Fig. 4

To avoid being misunderstood by readers, we also added the cobalt oxides area images of HAADF-STEM to the manuscript.

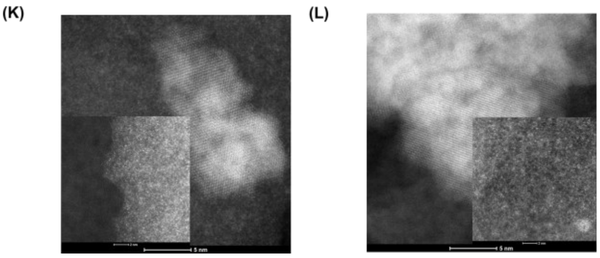

Fig 4. High-angle annular dark-field scanning transmission electron microscopy (HAADF-STEM) images of H-COP-Co (K) before and (L) after reaction, showing the single atom sites (insert image) and nanoparticle areas.

==

3. The ZAB performance is very poor, which lacks application promising. Could the authors compare it with other SAC-based ZABs with so poor performance?

==

Thanks for your comment. As we mentioned above, the main significance of this work is not about the performances. This report focused on the structural strategy of composed COPs, which contributed the enlightenment to extending the COPs to various applications with novel design strategies. The next generation of composite COPs was commonly believed as a promising material for frontiers in many fields. However, the morphology controlling, active species type, and content increase of COPs are still a significant challenge and important for application.
Moreover, we have compared with many advanced polymer-based ZAB performances and found that the H-COP-Co was excellent in them.

Supplementary Table 8. Summary of recently reported rechargeable Zn-air battery performances of other advanced polymer catalysts.

Sample	Peak power density (mW cm^{-2})	Specific capacity (mAh g^{-1})	Reference
H-COP-Co	139.5	787	This work
PcCuO$_8$-Co/CNT	94	700	*Angew Chem Int Ed.* 2019, 58, 10677–10682.
PCN-226(Co)	133	724	*J. Am. Chem. Soc.* 2020, 142, 15386–15395.
BHZ-48	148	-	*Nat Commun.* 2020, 11, 5858.
CC-3	85	714	*ACS Nano* 2021, 15, 3309–3319.
Azo-COF	134	~550	*Angew Chem Int Ed.* 2022, 61, e202209583.
JUC-650	101.5	722.6	*Small* 2024, 20, 2305759.
PD-COF-OH	111.6	676	*Angew Chem Int Ed.* 2025, 64, e202503434.
BDA-COF	101.8	-	*J. Energy Chem.* 2025, 108, 83–91.
Co-MOF-74-HATP@EC300J	96.6	-	*Nano Research.* 2025, 18, 94907195.
OH-COF	119.5	854	*Chem. Sci.* 2025. doi:10.1039/D5SC02145F.

==

We appreciate this reviewer very much for the above valuable comments that greatly improved the quality of the manuscript.

==

==

Reviewer #3 (Remarks to the Author):

COPs were one of the most promising functional materials in the energy fields. How to introduce active species and improve their densities is most desired to develop. This report first demonstrated the novel construction strategy of composited COPs that exceed the traditional post-modification strategies on active species density and performances. The theoretical calculation and in situ spectroscopies were well conducted to reveal the novel efficient OPM pathway on the H-COP-Co. This is significant work in the field of physics and chemistry of polymer materials, and the comprehensive in situ technology and characterization analysis showed a positive influence on the cross-fields. Minor revision is recommended before publication on Nature Communications.

==

Thanks for your encouragement and positive comments.

==

Q1. What is the product of pure ZIF-67 after the hot water disposal?

==

We appreciate this comment.

It is noticed that the ZIF-67 was unstable under the room temperature water condition (*ChemistrySelect* 2018, 3, 657; *Mater. Today Adv.* 2023, 20, 100448). We also used the carbon black as support for the hydrolyzed ZIF-67, and the PXRD result revealed retained ZIF peaks and new peaks from the product were assigned to the $Co(OH)_2$ phase.

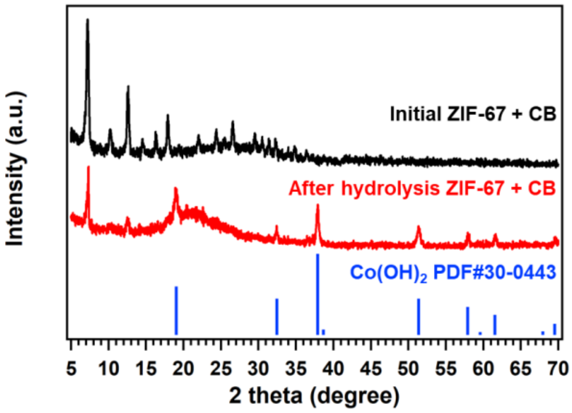

Fig. R5. PXRD patterns of the initial and treated ZIF67+CB.

==

Q2. The previous understanding of the different mechanisms of oxygen electrocatalysis pathway and chemical equations should be listed and explained.

==

We appreciate this comment.

We have added the different OER mechanisms to the manuscript.

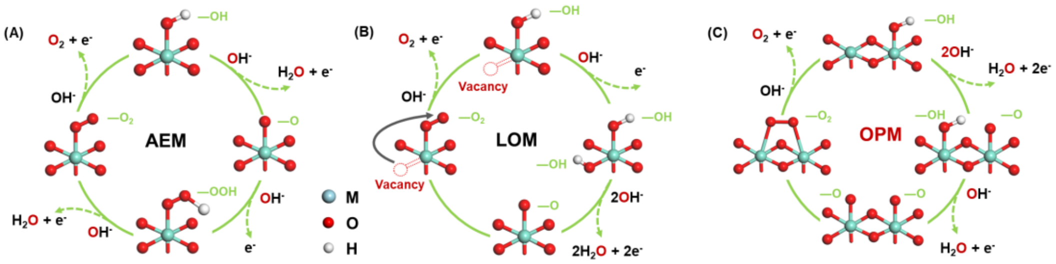

Supplementary Fig. 1. Schematic illustration of simplified OER mechanisms for (A) the AEM, (B) the LOM, and (C) the OPM.

==

Q3. The authors discussed the symmetry by XANES pre-edge analysis, and a detailed analysis is suggested.

==

We appreciate this comment. The pre-edge peaks are commonly believed to be attributed to the transition of 1s to the p component in d-p hybridized orbital, which depends on the coordination symmetry of the center metal (*X-Ray Spectrom*. 2008, 37: 572–584). From the octahedron to the tetrahedron of Co atoms, the increased p component in the hybridized orbital enhanced the pre-edge intensity.

We have added the sentences to the manuscript:

"The pre-edge peaks are attributed to the transition of 1s to the 4p component in d-p hybridized orbital and depend on the coordination symmetry of the center metal [52,53].

52. de Groot, F., Vankó, G. & Glatzel, P. The 1s x-ray absorption pre-edge structures in transition metal oxides. *J. Phys.: Condens. Matter* 21, 104207 (2009).

53. Yamamoto, T. Assignment of pre-edge peaks in K-edge X-ray absorption spectra of 3d transition metal compounds: electric dipole or quadrupole? *X-Ray Spectrometry* 37, 572–584 (2008)."

==

Q4. In Fig. 3D, the plotting of long-term tests seems not standard, a percentage description is better. The authors should adjust this.

==

We appreciate this comment. We have replotted the fig. 3D and displayed it as follows:

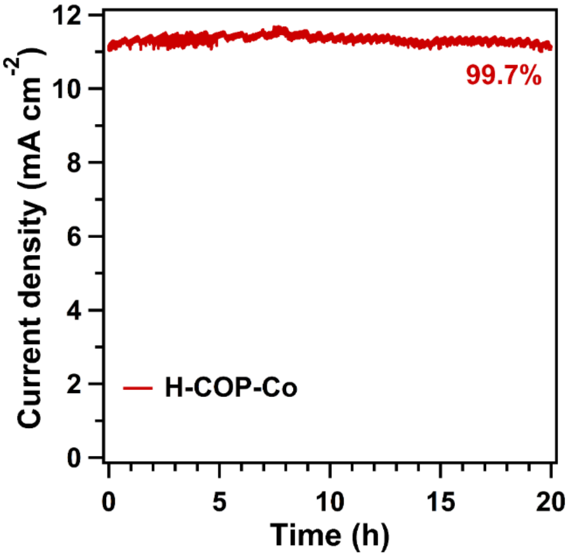

Fig. 3(D) Durability test of chronoamperometry curves for H-COP-Co.

==

Q5. The TGA tests of the S-COP-Co should be measured to illustrate the difference in the metal contents of the two construction strategies.

==

We appreciate this comment. We have added the TGA test of the S-COP-Co and plotted the TGA curve with that of H-COP-Co. The lower weight retention rate of S-COP-Co demonstrated its lower Co content compared to that of H-COP-Co.

We have added the sentence:

"This increase was consistent with the thermogravimetric curve of H-COP-Co, where high weight retention suggested higher Co content (Supplementary Fig. 8)."

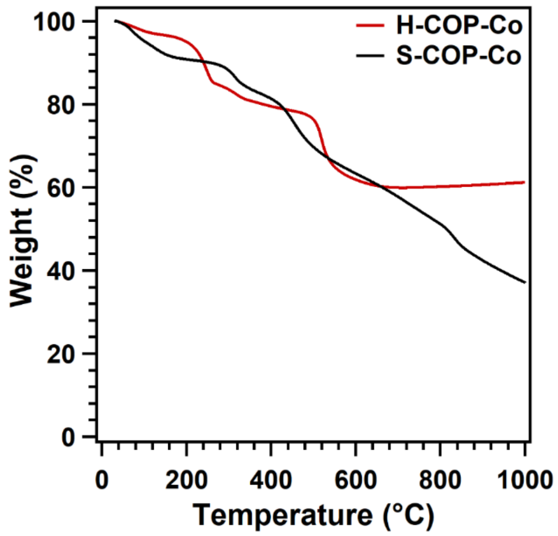

Supplementary Fig. 8. TGA profiles for H-COP-Co (red curve) and S-COP-Co (black curve) from room temperature to 1000 °C under N_2.

==

Q6. The authors lacked the FTIR spectra data of COPs that were essential to identify whether the polymers were successfully synthesized.

==

We appreciate this comment and have carefully read the manuscript and revised the faults.

We also updated related sentences as follows:

"In addition, the Fourier transform infrared spectroscopy (FTIR) measurements also demonstrated the COP coating on the ZIF-67 (Supplementary Fig. 3)."

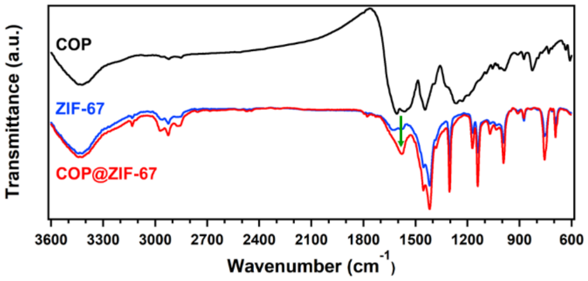

Supplementary Fig. 3. FTIR curves of the COP (black curve), ZIF-67 (blue curve), and COP@MOF (red curve).

==

Q7. The stability of COP should be assessed at the hot water conditions.

==

We appreciate this comment. We have used the same hot water according to the synthesis conditions of H-COP-Co, and immersed the COP for a longer period. The measured XRD displayed similar diffraction peaks that demonstrated the good stability of the COP part under the synthesis conditions of H-COP-Co.

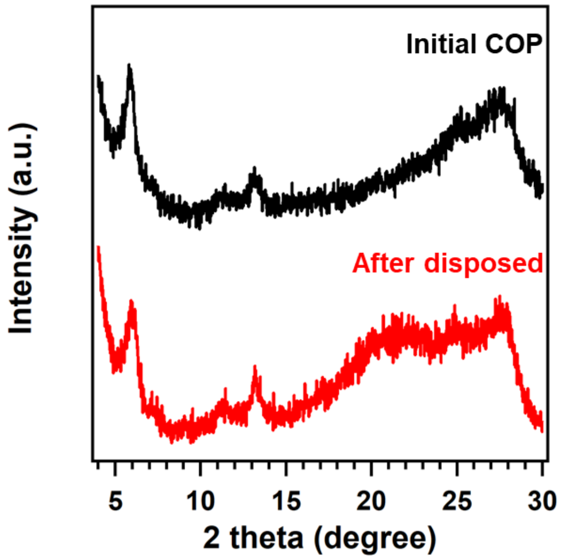

Fig. R6. PXRD patterns of the initial and after-treatment COP.

==

We appreciate this reviewer very much for the above valuable comments that greatly improved the quality of the manuscript.

==

==

Reviewer #4 (Remarks to the Author):

Fei et al. demonstrated the template-source construction strategy to synthesize the hollow COPs with highly active Co-O species. Many COPs studies focused on the single metal site design that optimizes traditional AEM pathways; this work first offers novel sights to design polymers and other energy materials through bottom-up methods to modulate the oxygen evolution reaction mechanism. Importantly, this study well implemented the in situ X-ray and infrared spectroscopies to explore the dynamic changes of catalytic sites, which gives us a new inspiration to study the catalytic mechanism. I recommend publication in Nat. Commun. after addressing the following comments:

==

Thanks for your encouragement and positive comments.

==

1) According to the procedure, the Co content of COP@MOF was high. However, the intensity from the XPS signal of COP@MOF is evidently lower than that from the H-COP-Co. The authors should explain the reason.

==

We appreciate this comment.

It is noticed that the XPS analysis is a typical surface probing method. As we know that the ZIF-67 is composed of many Co atoms. The COP@MOF was composed of the core of ZIF-67 and the shell of COP. Therefore, the surface signal to collect the Co element is difficult, since they were coated under the cobalt-free COPs. We also added the sentences to explain this phenomenon:

"It is noticed that the COP@MOF also contains a lot of Co, but displays a lower peak intensity in XPS, since the covered COP decreased the signal."

==

2) This work highlighted the improvement from the post-modification strategy. Was the S-COP-Co commonly reported in any fields? If it was, the authors should add a discussion.

==

We appreciate this comment. We have added the discussion about the S-COP-Co to the manuscript:

"The traditional post-synthetically modified COPs with single Co sites (collectively termed as S-COP-Co) have previously demonstrated high potential in the catalytic field [17,42].

17. Zhao, X. et al. Macro/microporous covalent organic frameworks for efficient electrocatalysis. *J. Am. Chem. Soc.* **141**, 6623–6630 (2019).
42. Aiyappa, H. B., Thote, J., Shinde, D. B., Banerjee, R. & Kurungot, S. Cobalt-modified covalent organic framework as a robust water oxidation electrocatalyst. *Chem. Mater.* **28**, 4375–4379 (2016)."

==

3) The authors used abbreviations many times, please give the full name when you first use them, such as "FT", "CV" and others.

==

We appreciate this comment. We have added the full name of these words when we first use them.

==

4) It seems the intermediate text in Fig. 5F is wrong. The authors should correct them.

==

We appreciate this comment. We have revised Fig. 5F as follows.

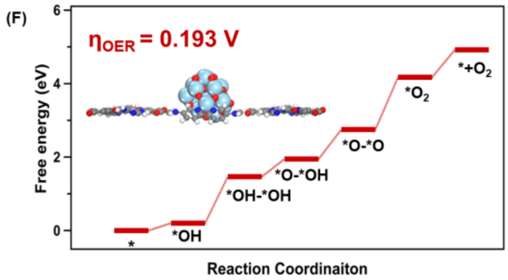

Fig. 5 (F) Free energy diagram of the OPM pathway at 1.23 V versus RHE.

==

5) The authors used many in situ technology to characterize the catalysts in the reaction. the in-situ instruments information is suggested to be provided.

==

We appreciate this comment. We have added the description and discussion to the manuscript:

We have revised the supporting information and added the Supplementary Fig. 19 and 24:

"**3. XAFS measurements and data processing**

XAFS measurements. The main XAFS measurements were performed at the BL20U1 station in Shanghai Synchrotron Radiation Facility (SSRF)[1]. The electron storage ring of SSRF was operated at 3.5 GeV, with a maximum current of 200 mA. Metals K-edge XAFS data were collected using a fixed-exit Si (111) double-crystal monochromator. A Lytle detector was used to collect the fluorescence signal, and the energy was calibrated using a metal foil. In situ Co K-edge XAFS measurements were conducted with catalyst-coated carbon paper as the working electrode, Ag/AgCl as the reference electrode, and a carbon rod as the counter electrode in an electrochemistry cell (Supplementary Fig. 19).

XAFS analysis. Utilizing the ATHENA module of the IFEFFIT software packages, the obtained EXAFS data were analyzed according to the standard procedures[2]. To determine the oxidation states of the Co atoms in the samples, reference Co oxides were used to extract standard absorption edge energies. A calibration curve was constructed by linear fitting of the normalized edge jumps of the reference compounds at an absorbance of 0.5. The EXAFS contributions were separated from different coordination shells by using a Hanning window (dk=1.0 $Å^{-1}$). Subsequently, the quantitative curve-fittings were carried out in the R-space with a Fourier transform k-space range using the module ARTEMIS of IFEFFIT. During the curve-fitting, the overall amplitude reduction factor S_0^2 was fixed to the best-fit value determined from fitting the data of the metal foil. For the sample, the structural parameters, such as the coordination number N, interatomic distance R, the Debye-Waller factor σ^2, and the edge-energy shift ΔE_0, were allowed to vary during the fitting process.

1. Mei, B.-B. et al. A seven-crystal spectrometer for high-energy resolution X-ray spectroscopy at Shanghai Synchrotron Radiation Facility. *Nucl. Sci. Tech.* **35**, 156 (2024).
2. Newville, M. IFEFFIT : interactive XAFS analysis and FEFF fitting. *J Synchrotron Rad.* **8**, 322–324 (2001)."

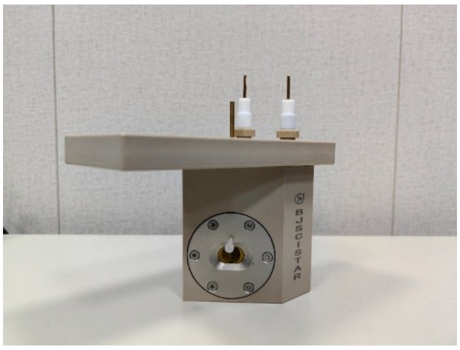

Supplementary Fig. 19. The picture of the in situ XAFS electrochemistry cell.

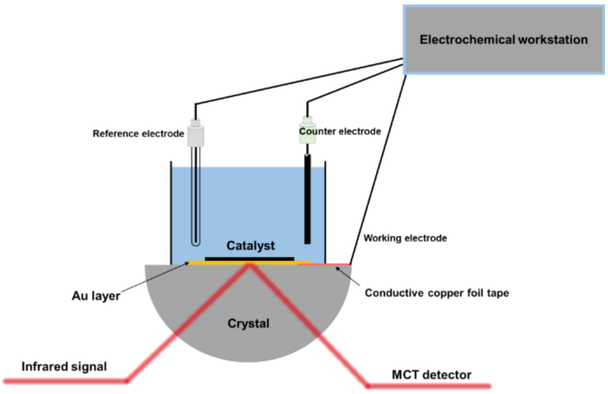

Supplementary Fig. 24. The schematic graph of the in situ electrochemical ATR-FTIR.

==

6) Some descriptions in the introduction are suggested for optimization.

==

We appreciate this comment and have carefully read the manuscript and revised the faults. Now the introduction part is revised as follows:

"Covalent organic polymers (COPs) are an emerging class of polymers created by assembling molecular organic building blocks via dynamic covalent bonds. By leveraging designable monomers, these polymers demonstrate porous structures, customizable structural configurations, thermal and chemical stability, and high surface areas [1–5]. These characteristics make COPs viable for several frontier applications, such as gas, liquid, and ion adsorption and separation; energy storage and conversion; photocatalysis; electrocatalysis; and life sciences [6–11]. Building upon these applications, composite COPs have gradually emerged as a new generation of polymers, offering significant advantages over single-site COPs owing to their extraordinary performance [12–16]. Despite these advancements, current composite COPs are typically modified post-synthetically to introduce catalytic sites, which are often limited in type and density[17,18]. Consequently, developing strategies to construct novel catalytic sites with high density remains a critical challenge and an area of particular interest.

The oxygen evolution reaction (OER) is a key process in water splitting and rechargeable metal-ion batteries, involving the coupling of multiple protons, electrons, and oxygen-containing intermediates [19–25]. Among the catalytic pathways for the OER, the adsorbate evolution mechanism (AEM) is widely acknowledged as an efficient route that proceeds through the adsorption of

intermediates, including *OH, *O, and *OOH [26–29]. This pathway is commonly facilitated by single-metal sites, which induce the monodentate adsorption of intermediates and thereby favor the AEM [30–33]. However, theoretical predictions indicate that the AEM is hindered by a high overpotential of approximately 370 ± 100 mV [34,35]. In contrast, the oxide path mechanism (OPM) represents an ideal catalytic pathway for the OER, as it prevents the generation of lattice defects and additional *OOH intermediates, resulting in a lower overpotential[36,37] (Supplementary Fig. 1). Despite these advantages, the application of the OPM remains challenging. COPs, known for their modifiable groups and ability to capture metal ions, are often employed to construct single-atom catalysts favoring single-site adsorption through the AEM pathway [38–41]. Establishing the COPs with high-density sites for a suitable adsorption structure induced the OPM pathway, which was anticipated to change the status quo in catalytic OER functional COPs design.

In this study, we utilized ZIF-67, a typical metal–organic framework (MOF), as a substrate to construct a core–shell (COP@MOF) structure via a mild covalent organic reaction of monomers. Subsequent warm hydrolysis of the ZIF-67 core while maintaining the COP shell intact led to Co capture by the constituent functional groups, resulting in the formation of cobalt oxide aggregates (collectively termed as H-COP-Co). The traditional post-synthetically modified COPs with single Co sites (collectively termed as S-COP-Co) have previously demonstrated high potential in the catalytic field [17,42]. Differently, the H-COP-Co exhibited a controlled morphology and high density of CoO_x clusters, which served as reactive sites for the OER. Furthermore, comprehensive in situ observations revealed that H-COP-Co tends to aggregate into cobalt oxides and facilitates OER via the OPM pathway, demonstrating superior performance compared to the AEM pathway adopted by S-COP-Co."

==

7) Some recent reports for OER catalysts are missing in the references.

==

We appreciate this comment.

We have added the OER reference, and now the OER part references are updated as follows:

19. Gao, Y. et al. Engineering spatial interface in supported molecular complexes for ampere-level electrocatalytic oxygen evolution. *J. Am. Chem. Soc.* **147**, 18760–18769 (2025).
20. Gu, P. et al. Crystalline-amorphous interface-triggered electron redistribution on copper(II) sulfide@metal (Ni, Co, and Fe) oxyhydroxides for ultra-efficient overall water/seawater splitting. *Adv. Energy Mater.* **15**, 2403657 (2025).
21. Yao, B. et al. Iron-induced localized oxide path mechanism enables efficient and stable water oxidation. *Angew. Chem., Int. Ed.* **64**, e202416141 (2025).
22. Wen, J. et al. Activating inert palmeirite through Co local-environment modulation and spin electrons rearrangement for superior oxygen evolution. *Adv. Energy Mater.* **15**, 2405555 (2025).
23. Feng, Z. et al. Seven mechanisms of oxygen evolution reaction proposed recently: A mini review. *Chem. Eng. J.* **485**, 149992 (2024).
24. Wang, Z., Goddard, W. A. & Xiao, H. Potential-dependent transition of reaction mechanisms for oxygen evolution on layered double hydroxides. *Nat Commun.* **14**, 4228 (2023).
25. Sun, X. et al. Recent advances in perovskite oxides for oxygen evolution reaction: structures, mechanisms, and strategies for performance enhancement. *Adv. Funct. Mater.* 2416705 (2024).

==

We appreciate this reviewer very much for the above valuable comments that greatly improved the quality of the manuscript.

==

Title: Constructing High-Density Active Sites on Hollow Covalent Organic Polymers for Efficient Oxygen Electrocatalysis

The present manuscript demonstrates the synthesis of CoNPs embedded in a Covalent Organic Polymer (COP) framework, which is used in the polymerization of COPs on ZIF-67, followed by the hydrothermal degradation of ZIF-67. The obtained materials were characterized using various techniques, including XAS, which demonstrated the presence of Co-Co coordination and Co-N/C coordination. Based on linear combination fitting, EXAFS, and pre-edge feature area ratio analysis, the authors proposed the presence of Co-O nanoparticles and a relatively small amount of Co single-atom sites in the catalyst. When tested for the OER and ORR, the catalysts exhibited reduced overpotential and Tafel slopes, comparable to standard Pt and RuO_2 catalysts. DFT analysis revealed that OPM was the dominant pathway for the reaction, while S-COP-Co followed the AEM pathway. The catalyst also demonstrated improved activity in ZABs. Despite the appealing findings presented in the manuscript, the exact chemical and electronic properties of the catalysts are not well understood. Additionally, many control experiments are required to probe the role of Co-O sites in the observed activity. Several critical questions need to be addressed before the manuscript can be considered for publication. The key comments are as follows:

1. While the authors have designed an interesting core-centered H-COP-Co assembly, the motivation for why CoNPs deposited on any surface or a non-core-shell assembly would not exhibit similar catalytic activity is unclear. A control sample without a core-shell structure on COP and other surfaces should be prepared, and its catalytic activity should be compared.
2. The evidence for the presence of COPs in COP@ZIF is not very clear. Additionally, it is unclear whether COPs completely covered ZIF-67. To address this, TGA data for each component should be provided, along with derivative TGA analysis to highlight the temperatures at which major mass loss occurs. Further, FTIR or Raman spectra of ZIF, COP, and ZIF@COP should be included to confirm the presence of ZIF inside the COP structure.
3. The high energy of the electron beam in TEM can alter the sample morphology. Therefore, SEM images and elemental mapping should be provided. What is the size of the Co-O nanoparticles? High-magnification TEM images should be included with d-spacing measurements. In Figure 1c, TEM images of materials should be presented instead of HAADF.
4. HAADF-STEM images of the materials at high magnification should be included to clearly show the nanoparticles and single atoms.
5. It remains unclear whether the observed catalytic activity is due to CoNPs or the high concentration of Co atoms in the catalyst. To distinguish their roles, the authors should remove the nanoparticles via dilute acid washing and test the remaining CoSA-COP-Co for OER and ORR activity.
6. The authors claim that Co is present as CoO nanoparticles based on XANES and EXAFS data. However, the EXAFS spectra of H-COP-Co appear

to resemble those of Co nanoparticles rather than Co-O. Additionally, the comparison between Co NP and Co SACs was calculated using these peaks, which may be a misinterpretation. These discrepancies should be clarified.

7. To confirm that Co-O sites, rather than metallic Co sites, are responsible for the main oxidation activity, the H-COP-Co catalyst should be treated with weak H_2O_2 to oxidize metallic Co to Co-O, and its catalytic activity should be reassessed.
8. The degradation of ZIF-67 under hydrothermal conditions should be confirmed using other support materials. A reference should be provided to support this claim.
9. The structural model presented suggests that only one Co atom is coordinated to two nitrogen atoms, while another Co is present as Co-O. In this case, the average coordination number for Co-N should be much lower than for Co-O. However, the authors report a Co-N:Co-O ratio of approximately 1:2. Similarly, the Co-Co coordination number of ~4 does not support the presence of Co in nanoparticle form. These inconsistencies should be addressed.
10. Why does the XRD pattern of H-COP-Co not exhibit any peaks related to CoO? Is this due to the thickness of the COP shell or the small nanoparticle size? The shell thickness should be reported.
11. What was the oxidation state of Co in ZIF-67? The origin of the satellite peak in the XPS spectra of ZIF-67 should be clarified.
12. The white line intensity of S-COP-Co is higher than that of H-COP-Co, yet the oxidation state determination does not reflect this trend. The methodology used to calculate the oxidation state of Co in each material based on XANES white line intensity should be clearly explained. Did the authors use the center point of the linear region of the highest peak intensity for the calculation?
13. Figures 2d and 2i-j appear significantly different in terms of fitting. The fitting parameters used should be described in the supporting information. Additionally, details about the XANES and EXAFS measurement conditions, beamline parameters, and the in-situ measurement cell setup should be included. A picture of the inset FTIR cell should also be provided.
14. EIS spectra of the catalyst should be included. The onset potential and fitting parameters should be provided to assess charge transport and charge transfer resistance.
15. The change in Co-Co peak intensity upon applying a high potential suggests nanoparticle formation. This could be due to either the agglomeration of atoms, as the authors suggest, or the degradation of COPs at this potential. The authors should confirm whether the support structure remains chemically and physically intact after the reaction.
16. The chemical structure of COP suggests that it is not conductive. However, the material still delivers a significant current density, as shown in Figure 3a. Furthermore, long-term stability was tested at a very low current density. In industry-standard testing, stability is typically demonstrated at a current density of 10 mA cm^{-2}.

17. Bader charge analysis, electron density distribution plots, and density of state calculations should be added to the DFT analysis.
18. The C, N, O, and Co content reported by XPS quantification should be specified as either weight percent (wt%) or atomic percent (at%). Both values should be provided in the supplementary information. Additionally, the elemental composition of materials after the reaction should be reported. The metal content after the reaction should also be analyzed using ICP.
19. Several grammatical and formatting errors need to be corrected. The purity of each chemical should be included in the supplementary experimental section. Instrument operating conditions and sample preparation details should be clearly defined. In Supplementary Figure 7b, the Y-axis should be corrected.

Additional suggestions:

- Avoid using hyperbolic terminology, such as "exceptional."
- Define "reversible covalent reactions" clearly.
- The sentence "In contrast, S-COP-Co and the COP exhibited similar weak diffraction…" is incorrect. Single atoms do not exhibit diffraction peaks, and the absence of diffraction peaks is not direct evidence of the presence of single atoms.
- The phrase "smooth cubic structures" is not appropriate.
- Reference formatting should be consistent with journal guidelines—titles should be in either sentence case or title case.
